# Mechanisms of Spica Prunellae against thyroid-associated Ophthalmopathy based on network pharmacology and molecular docking

**DOI:** 10.1186/s12906-020-03022-2

**Published:** 2020-07-20

**Authors:** Yuhan Zhang, Xianzhi Li, Congcong Guo, Jianjun Dong, Lin Liao

**Affiliations:** 1 Department of Endocrinology and Metabology, Shandong Provincial Qianfoshan Hospital, Cheeloo College of Medicine, Shandong University, Jinan, 250014 China; 2 Laboratory of Endocrinology, Medical Research Center, Shandong Provincial Qianfoshan Hospital, the First Affiliated Hospital of Shandong First Medical University, Jinan, 250014 Shandong China; 3 Department of Endocrinology, Affiliated Hospital of Shandong University of Traditional Chinese Medicine, Jinan, 250011 China; 4 Division of Endocrinology, Department of Internal Medicine, Qilu Hospital of Shandong University, Jinan, 250012 China; 5 Department of Endocrinology and Metabology, the First Affiliated Hospital of Shandong First Medical University, Ji-nan, 250014 China

**Keywords:** Spica Prunellae, Thyroid-associated ophthalmopathy, TAO, Network pharmacology, Molecular docking

## Abstract

**Background:**

Thyroid-associated ophthalmopathy (TAO) is an autoimmune inflammatory disorder, which lacks effective treatment currently. Spica Prunellae (SP) is popularly used for its anti-inflammatory and immune-regulating properties, indicating SP may have potential therapeutic value in TAO. Therefore, the purpose of this study is to identify the efficiency and potential mechanism of SP in treating TAO.

**Methods:**

A network pharmacology integrated molecular docking strategy was used to predict the underlying molecular mechanism of treating TAO. Firstly, the active compounds of SP were obtained from TCMSP database and literature research. Then we collected the putative targets of SP and TAO based on multi-sources databases to generate networks. Network topology analysis, GO and KEGG pathway enrichment analysis were performed to screen the key targets and mechanism. Furthermore, molecular docking simulation provided an assessment tool for verifying drug and target binding.

**Results:**

Our results showed that 8 targets (PTGS2, MAPK3, AKT1, TNF, MAPK1, CASP3, IL6, MMP9) were recognized as key therapeutic targets with excellent binding affinity after network analysis and molecular docking-based virtual screening. The results of enrichment analysis suggested that the underlying mechanism was mainly focused on the biological processes and pathways associated with immune inflammation, proliferation, and apoptosis. Notably, the key pathway was considered as the PI3K-AKT signaling pathway.

**Conclusion:**

In summary, the present study elucidates that SP may suppress inflammation and proliferation and promote apoptosis through the PI3K-AKT pathway, which makes SP a potential treatment against TAO. And this study offers new reference points for future experimental research and provides a scientific basis for more widespread clinical application.

## Background

Thyroid associated ophthalmopathy (TAO), an inflammatory disorder affecting the orbit and ocular adnexa, is occurred in autoimmune thyroid disease, mainly Graves’ disease [1]. A prospective study determined that the overall incidence rate of TAO was 10 per 10,000 persons in Europe [2]. And it is reported to occur in 20–30% of patients with Graves’ disease and 2% of patients with Hashimoto’s thyroiditis [3–5]. The pathogenesis of TAO is related to many factors, including genetic factors, environmental factors and autoimmunity, in which autoimmune disorder plays an essential role. An intricate interplay between the potential pathogenic autoantigens and the autoantibodies leads to the activation of autoimmune response and releasing related inflammatory factors, resulting in inflammation of the orbital tissues, abnormal fibroblast proliferation, and extraocular muscle thickening [5–7]. Clinically, it is characterized by proptosis, upper eyelid retraction, restrictive strabismus, exposure keratopathy and other presentation. As a result, its impact on vision and appearance in daily living is significantly associated with the quality of life and psychosocial problem [8–11]. However, current treatments are inadequate due to the incompletely understood pathophysiology of TAO. For mild disease, most patients experience spontaneous recovery within about 6 months [12]. Although helping with symptom management, 13.5% of patients had a progressively clinical deterioration [13]. For active severe phase, corticosteroids remain the mainstay treatment and orbital radiotherapy commonly serves as an adjuvant therapy [14–16]. However, these therapeutic approaches are often accompanied with limited efficacy and safety concerns, such as hormone resistance and complications [5]. Therefore, there is an urgent demand for novel effective therapy to improve the progression of TAO.

Spica Prunellae (SP), the fruit-spikes of the *Prunella vulgaris L.*, is a traditional antipyretic Chinese herb with a wide distribution in Northeast Asia. According to the theory of traditional Chinese medicine (TCM), SP has long been thought to have benefits involving clearing the fire and eyesight, relieving edema and removing stasis. Moreover, SP has been extensively used in food additives and pharmaceuticals [17]. Previous pharmacologic studies indicated that the SP has multiple biological functions such as anti-inflammatory [18], anti-cancer [19, 20] and immunomodulatory activities [21]. Nowadays, SP has a wide range of applications in thyroid disease, for instance, thyroid gland swell and subacute thyroiditis [22, 23]. Indeed, some Chinese herbal formulations applied to TAO therapy employ SP as an important ingredient [24]. However, the underlying mechanism is yet to be completely elucidated.

Based on the development of bioinformatics, network biology and pharmacology analysis, network pharmacology becomes a novel strategy to understand the law of interactions between multicomponent and multitarget systematically and comprehensively [25, 26]. In the latest years, network pharmacology has been successfully used to predict the mechanism of TCM in a variety of diseases treatment. Therefore, we adopted the network pharmacology approach to investigate the potential therapeutic targets and pathways of SP in TAO treating, and performed molecular docking studies to further predict the recognition and interaction modes between SP and its predicted targets.

## Methods

### Screening of active compounds of SP

To collect the pharmacologically active compounds of SP, we searched Traditional Chinese Medicine System Pharmacology Database [27] (TCMSP, http://lsp.nwu.edu.cn/tcmsp.php), a systematic pharmacology platform of Chinese herbal medicine. Based on their ADME (absorption, distribution, metabolism and excretion) features, Oral bioavailability (OB) and Drug-likeness (DL) were set as the key parameters [28]. OB refers to the percentage of which the pharmaceutical agents is used and unchanged throughout systemic circulation [29]. And DL is a concept of optimize pharmacokinetics and properties [30]. Then, based on the threshold values of OB 30% and DL 0.18 [31], eleven active compounds were selected. In addition, five researches [32–36] using high-performance liquid chromatography (HPLC) to identify active compounds of SP were screened through literature search. After integrating with the results obtained from TCMSP, total twenty active compounds of SP were used to the subsequent network pharmacology study.

### Prediction of putative targets of SP

The putative targets of active compounds were extracted from the TCMSP database [27] (http://lsp.nwu.edu.cn/tcmsp.php), the PubChem database [37] (http://pubchem.ncbi.nlm.nih.gov), the Swiss Target Prediction database [38] (http://www.swisstargetprediction.ch/), and the STITCH database [39] (http://stitch.embl.de/). Then, all the targets names were put into uniprot sites (http://www.uniprot.org/) and selected by Homo Saipan species to normalize the gene information. Detailed information of putative targets is provided in Table S1.

### Identification of TAO related targets

Related TAO targets were gathered from multi-sources databases using “Thyroid-associated ophthalmopathy” and “graves ophthalmopathy” as keywords. Five database are as follow: (1) the Therapeutic Target Database [40] (TTD, https://db.idrblab.org/ttd/); (2) the Online Mendelian Inheritance in Man database [41] (OMIM, http://omim.org/); (3) the Comparative Toxicogenomics Database [42] (CTD, http://ctdbase.org/); (4) the DrugBank database [43] (https://www.drugbank.ca/); (5) the GeneCards database [44] (https://www.genecards.org/). Detailed information is described in Table S2 and Table S3.

### Analysis of overlapping targets of SP in TAO

#### Target mapping

To obtain the candidate targets responsible for TAO therapy, the putative targets of SP mapped to the TAO related targets to obtain overlapping targets. For overlapping targets, numbers were visualized with a Venn diagram generated in R version 3.2.0, a freely available, open-source statistical programming language and environment for statistical computing [45], with VennDiagram package.

#### Protein-protein interaction (PPI) analysis

These related targets of SP in treating TAO were then put in STRING tools [46] (https://string-db.org/), an online platform that critically predict functional protein association networks, to generate protein interaction network. Network data were used for the topological properties analysis to identify important targets in anti-TAO system, and Table S4 for more information.

#### Gene ontology (GO) and KEGG pathway enrichment analysis

To further reveal the potential mechanism of SP in TAO treating, we imported overlapping targets into the Functional Annotation tool of Database for Annotation, Visualization and Integrated Discovery (DAVID) 6.8 [47] (https://david.ncifcrf.gov/) to perform GO functional analysis and KEGG pathways analysis. The enrichment *P* values of functional annotations were corrected by both Bonferroni (*P* < 0.05) and Benjamini (*P* < 0.05) correction. The plots of GO enrichment and KEGG pathway enrichment were then carried out with R version 3.2.0.

#### Network construction

The Cytoscape3.6.1 [48] (http://cytoscape.org/), a software environment for integrated models of biomolecular interaction networks, was utilized to construct the following networks:(1) Compound-target network; (2) PPI network of TAO targets; (3) PPI network of compound-TAO targets; (4) Compound-target-pathway network.

The Cytoscape plug-in Network Analyzer was applied to analyze the topological properties of the PPI network. Three indices were used to assess the topological properties of every node. (1) “Degree” refers to the number of nodes that directly interact with that node in a network [49], (2) “Betweenness Centrality”: the proportion of the number of nodes that have passed through the shortest path in a network [50], with important influence due to their control over information passing between other nosdes. Thus, the betweenness reflects its global importance, while the degree measures the local importance of a node; (3) “Closeness Centrality” reflects the closeness of a node to other nodes [51]. The level of these three paraments reflects the topological importance of the node in a network, which the higher the values are, the more important the nodes are. The targets with degree > twofold median were selected as the hub genes and used for further molecular docking.

### Molecular docking simulation

#### Target protein preparation

The crystal structures of hub genes were downloaded from RCSB Protein Data Bank [52] (http://www.pdb.org/). The downloaded complexes were embellished by PyMol2.3.0 (the PyMol Molecular Graphics System) to remove original ligand, water molecules and phosphates. Moreover, the AutoDock Tools1.5.6 [53] (http://mgltools.scripps.edu/documentation/links/autodock) was used to prepare receptors, including adding hydrogen and seting docking paraments. “Grid box” was set to maximum to perform the blind docking.

#### Ligand preparation

Before docking, the 2D structures of compounds were drawn using the ChemBioDraw13.0 software. Then the ChemBio3D13.0 software was used to transfer the 2D structure to 3D chemical structure and make energy minimizing for the further docking.

#### Molecular docking

All ligand and receptor files were saved as pdbqt format. Then we used Autodock Vina [54], a freely available open-source packages, to evaluate and verify the binding affinity of compound-target relationship, and the prediction results from network pharmacology. The binding models were visualized by PyMol2.3.0 software and Discovery Studio3.5 software.

## Results

### Compound-target network

**Fig. 1:**
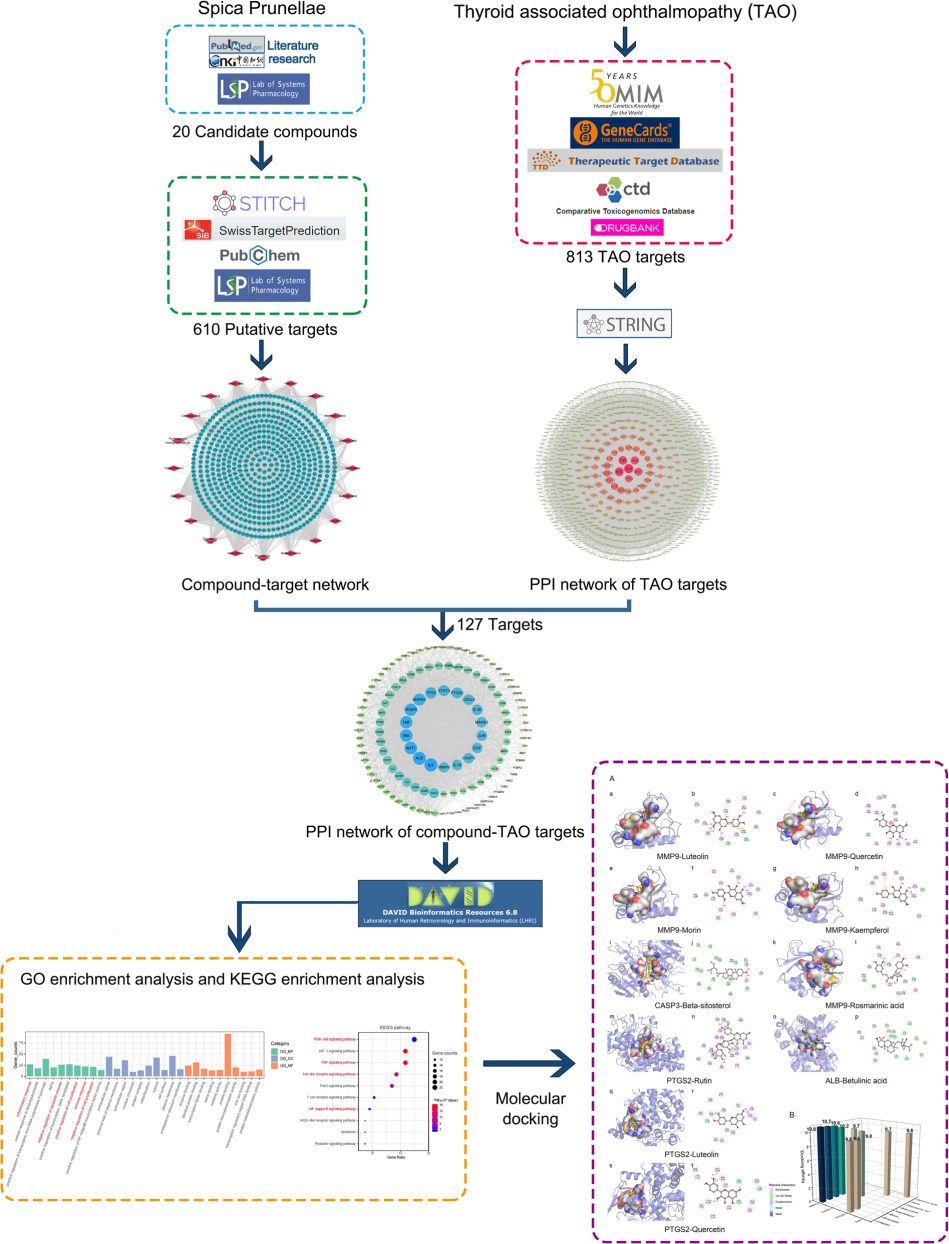
Flowchart of investigating the mechanism of SP in TAO treatment

**Fig. 2:**
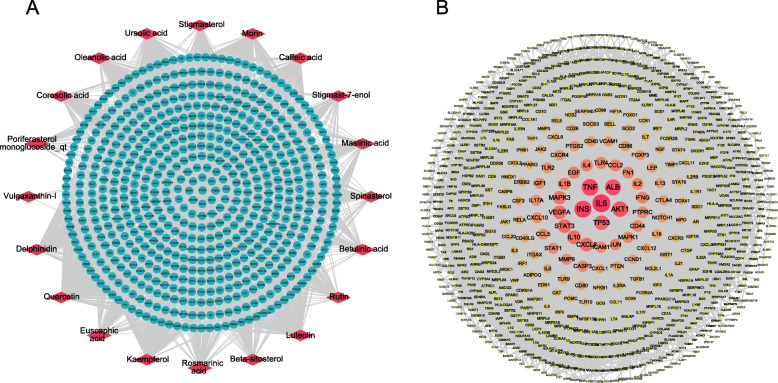
Compound-target network and PPI network of TAO targets. **a** Compound-target network. Red diamond nodes represent active compounds of SP and blue circular nodes represent corresponding targets. **b** PPI network of TAO targets. The sizes and colors of the nodes are illustrated from big to small and green to red in descending order of degree values

A flowchart of the network pharmacology study is summarized in Fig. 1. Based on TCMSP, 11 active compounds were selected by the screen criteria of OB and DL. Considering the large number of researches performed HPLC to identify the major active components of SP, we conducted the literature search in Pubmed and China National Knowledge Infrastructure (CNKI). And nine compounds were supplemented accounting for the relatively large proportion and proven active role in SP, including Betulinic acid, Caffeic acid, Corosolic acid, Euscaphic acid, Maslinic acid, Rosmarinic acid, Rutin, Oleanolic acid and Ursolic acid (Table 1). Then we identified 610 putative targets from TCMSP, Pubchem, STITCH and SwissTargetPrediction, which the detailed information is described in Table S1. To obtain further understanding of the relationship between the 20 compounds and their corresponding targets on a system level, we conducted the compound-target network (Fig. 2a). In a network topology analysis, we identified top six compounds adopting the average degree (103.94) as the threshold value: Quercetin (degree = 298), Ursolic acid (degree = 155), Luteolin (degree = 149), Kaempferol (degree = 146), Morin (degree = 135) and Delphinidin (degree = 113).

**Table 1:**
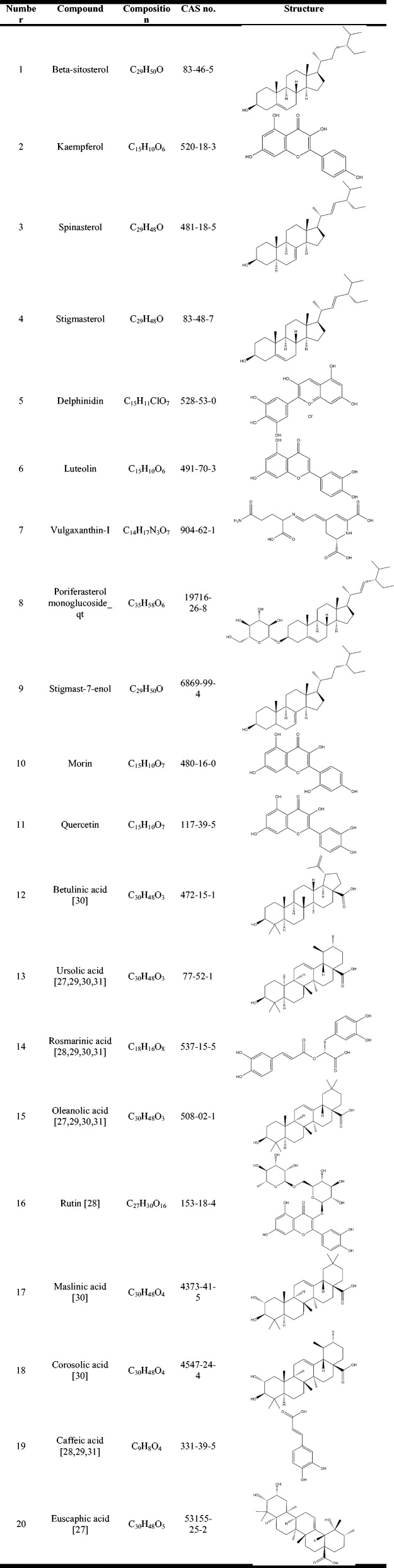
Chemical information for the active compounds of SP

### TAO target network

In the present study, a total of 813 proteins were documented as the disease potential targets of TAO (Table S2). As shown in Fig. 2b, the PPI network reflected the interaction between these targets. Six targets highly related to the pathological process of TAO, which were sorted according to the node degree, including IL6 (interleukin-6, degree = 262), INS (insulin, degree = 247), TNF (tumor necrosis factor, degree = 242), ALB (serum albumin, degree = 229), AKT1 (RAC-alpha serine/threonine-protein kinase, degree = 221) and TP53 (tumor protein p53, degree = 205) (Table S3).

### PPI network of compound-TAO targets

**Fig. 3:**
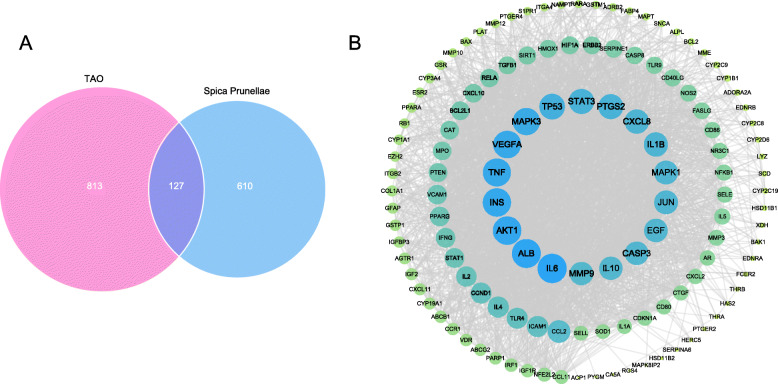
Venn diagram and PPI network of compound-TAO targets. **a** Venn diagram of intersecting targets of SP and TAO. **b** PPI network of compound-TAO targets. The sizes and colors of the nodes are illustrated from big to small and green to blue in descending order of degree values

Based on the above results, the 610 putative targets of SP mapped to the 813 TAO related targets to obtain overlapping targets. Consequently, 127 targets were identified as the candidate targets responsible for TAO therapy (Fig. 3a). A PPI network was then constructed to evaluate the role of the targets in complex disease and discover the interactive effects. After analyzing the topological feature of the abovementioned PPI network, 127 genes were sorted in descending order by degree in Table S4 with an average degree of 36.74. As shown in Fig. 3b, nodes arranged in a concentric circle according to the degree and the innermost center circle is composed of 18 hub nodes, including AKT1, ALB, CASP3 (caspase-3), CXCL8 (interleukin-8), EGF (epidermal growth factor), IL10 (interleukin-10), IL1B (interleukin-1 beta), IL6, INS, JUN (transcription factor AP-1), MAPK1 (mitogen-activated protein kinase 1), MAPK3 (mitogen-activated protein kinase 3), MMP9 (matrix metallopeptidase 9), PTGS2 (prostaglandin-endoperoxide synthase 2), STAT3 (signal transducer and activator of transcription 3), TP53 and VEGFA (vascular endothelial growth factor A). These hub genes are of significance in the network and used for the following molecular docking study.

### GO and KEGG pathway enrichment analysis

**Fig. 4:**
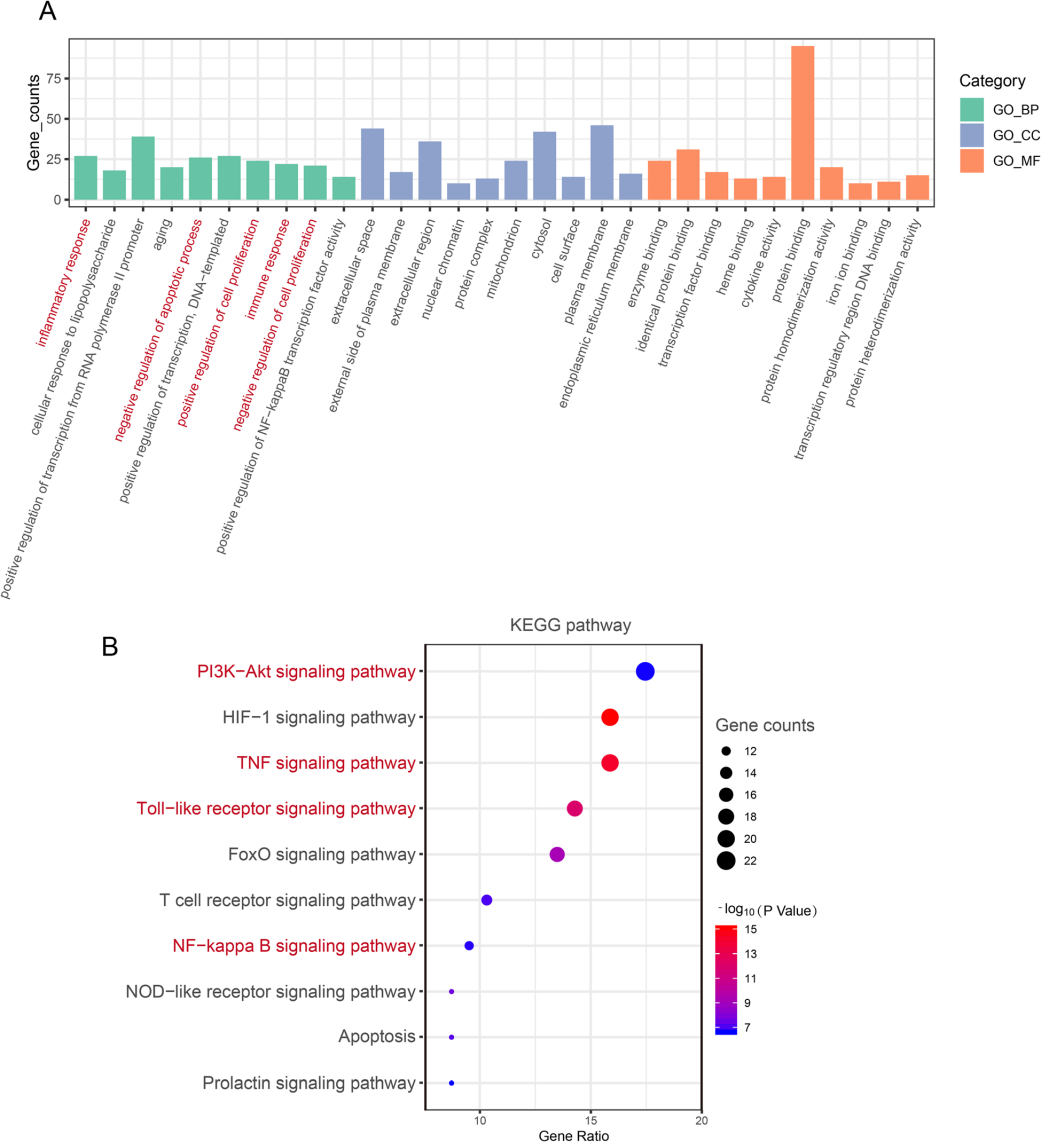
Enrichment analysis of potential targets. **a** GO enrichment analysis. The top 10 terms of each part are shown. BP: biological processes, CC: cell component, MF: molecular function. **b** KEGG pathway analysis. The sizes of the bubbles are illustrated from big to small in descending order of the number of the potential targets involved in the pathways

To further elucidate the mechanism of drug treatment systematically, the enrichment analysis was performed with DAVID on 127 genes. The top 10 GO items and KEGG pathways are selected based on counts of hit genes and *P* values (Fig. 4). For biological processes, it can be found that the targets were mainly enriched in inflammatory response (GO:0006954, GO:0071222, GO:0051092), cell proliferation (GO:0008284, GO:0008285), apoptosis (GO:0043066) and immune response (GO:0006955). Additionally, the other three functional annotations are associated with transcriptional regulation (GO:0045944, GO:0045893) and aging (GO:0007568). For pathway analysis, most involved in immune and inflammatory related pathways such as PI3K-AKT signaling pathway (hsa04151), NOD-like receptor signaling pathway (hsa04621), Toll-like receptor signaling pathway (hsa04620), NF-kappa B signaling pathway (hsa04064) and TNF signaling pathway (hsa04668) (Table 2). These results demonstrated that the main action mechanism underlying TAO treatment.

**Table 2:** Information for top 10 pathways

Number	Pathway name	Count	Corresponding hub genes	*P* Value
1	PI3K-Akt signaling pathway	22	AKT1, EGF, IL6, INS, MAPK1, MAPK3, TP53, VEGFA	2.25E-07
2	HIF-1 signaling pathway	20	AKT1, EGF, IL6, INS, MAPK1, MAPK3, STAT3, VEGFA	8.24E-16
3	TNF signaling pathway	20	AKT1, CASP3, IL1B, IL6, JUN, MAPK1, MAPK3, MMP9, PTGS2, TNF	7.25E-15
4	Toll-like receptor signaling pathway	18	AKT1, CXCL8, IL1B, IL6, JUN, MAPK1, MAPK3, TNF	1.23E-12
5	FoxO signaling pathway	17	AKT1, EGF, IL10, IL6, INS, MAPK1, MAPK3, STAT3	5.82E-10
6	T cell receptor signaling pathway	13	AKT1, IL10, JUN, MAPK1, MAPK3, TNF	8.99E-08
7	NF-κB signaling pathway	12	CXCL8, IL1B, PTGS2, TNF	1.85E-07
8	NOD-like receptor signaling pathway	11	CXCL8, IL1B, IL6, MAPK1, MAPK3, TNF	2.33E-08
9	Apoptosis	11	CASP3, TNF, TP53, AKT1	6.48E-08
10	Prolactin signaling pathway	11	AKT1, INS, MAPK1, MAPK3, STAT3	2.45E-07

### Molecular docking analysis

**Fig. 5:**
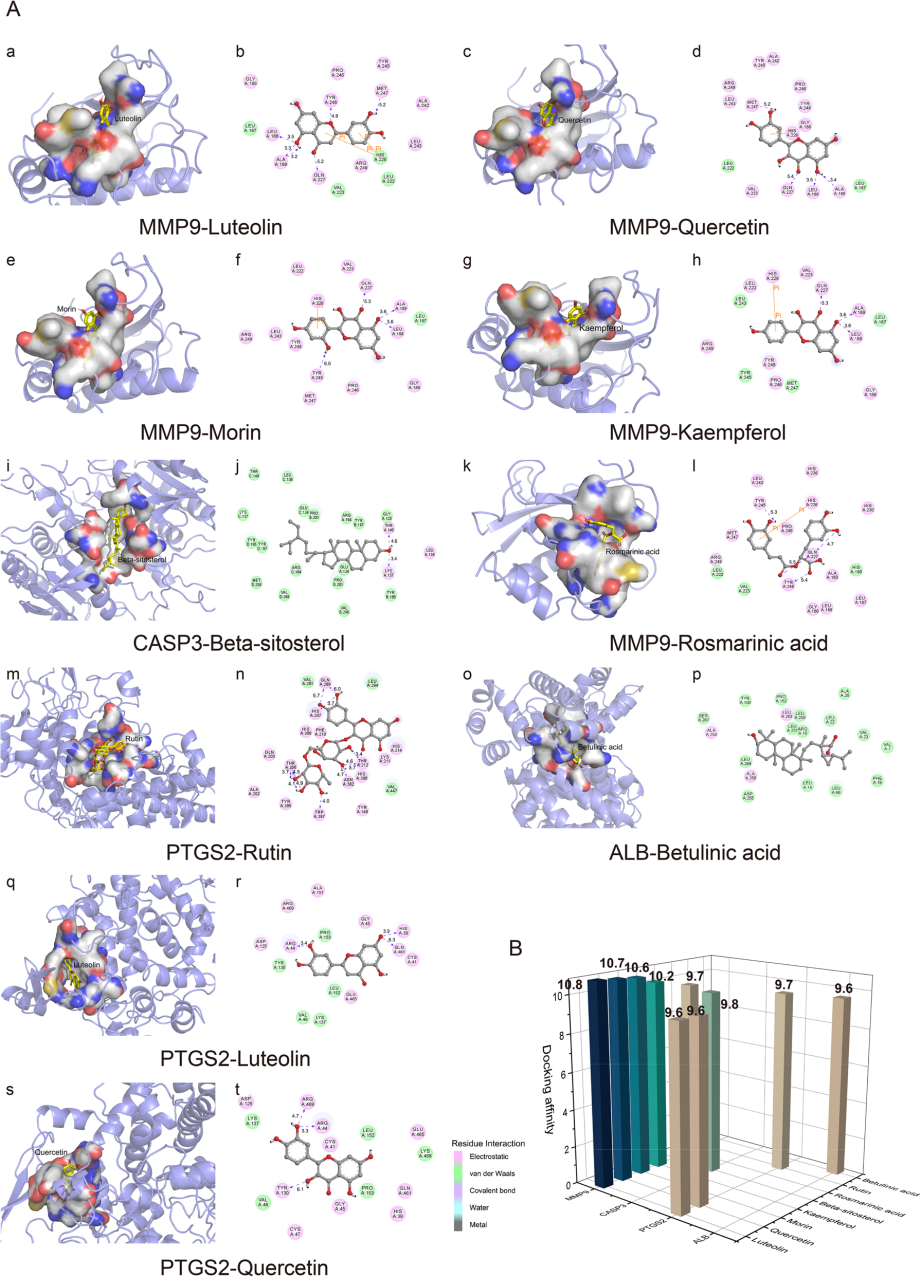
Molecular docking models of active compounds binding to potential targets. **a** The top 10 pairs of molecular docking simulation are shown. Schematics (3D) represent that molecular model of the compound is in the binding pocket of the protein. The compounds are shown as stick model with yellow colored. The amino acid residues surrounding are represented by surface. Schematics (2D) show the interactions between compounds and surrounding residues. The purple dashed lines represent hydrogen bonds and the interaction distances are indicated beside to the bonds. The orange lines demarcate pi-pi stacking interactions. **b** 3D column diagram shows the affinity of 10 pairs docking models. X-axis: protein names, Y-axis: active compounds, Z-axis: the absolute value of the docking affinity

In the present studies, the possible interaction activity between 18 hub genes and their corresponding compounds of SP was investigated with molecular docking verification. Meanwhlie, the retrieved targets and active compounds were further filtered by the docking affinity values reported by AutoDock Vina. There were total seventy-three pairs delivered into the docking simulation (Table 3). The greater the absolute value of the docking affinity, the stronger binding ability between the compounds and the active site of the targets. Among the docking results, most binding complexes possessed high binding affinity with an average of − 8.1 kcal/mol. The modes of top 10 binding complexes are displayed in Fig. 5, including MMP9-Luteolin docking (− 10.8 kcal/mol), MMP9-Quercetin docking (− 19.7 kcal/mol), MMP9-Morin docking (− 10.6 kcal/mol), MMP9-Kaempferol (− 10.2 kcal/mol), CASP3-Beta-sitosterol docking (− 9.8 kcal/mol), MMP9- Rosmarinic acid docking (− 9.7 kcal/mol), PTGS2-Rutin docking(− 9.7 kcal/mol), ALB-Betulinic acid docking (− 9.6 kcal/mol), PTGS2-Luteolin (− 9.6 kcal/mol), PTGS2-Quercetin (− 9.6 kcal/mol). For concreteness, taking the MMP9-Luteolin docking as an example, small molecule ligand Luteolin could potentially fit into the interface pocket formed by interaction amino acid residues in protein (Fig. 5A (a)). It showed that six hydrogen bond formation between ligand and residues invloved in LEU 188, ALA 189, GLN 227, TYR 248 and MET 247. Three hydrogen bond interactions were considered as strong interaction with the distance of 3.2, 3.3, 3.5 Å respectively. Moreover, HIS 226 residue contributed to form two pi-pi stacking interactions with two benzene rings of ligand. The other essential residues (GLY 186, LEU 187, VAL 223, ARG 249, LEU 222, LEU 243, ALA 242, TYR 245 and PRO 246) interacted with Luteolin through electrostatic forces, van der Waals forces, etc. (Fig. 5A(b)). Consequently, Luteolin stably binded to MMP9 protein bonds through mutiple interaction forces. Overall, we found that hydrogen bond and electrostatic forces were the main forms of interactions of the top 10 docking complexes, and the diverse of the interaction forms determined the ability of binding affinity.

**Table 3:** Results of 18 hub genes and compounds of SP molecular docking

Number	Hub genes	PDB ID	Compound	Docking affinity (kcal/mol)
1	AKT1	4EKL	Luteolin	-8.6
			Delphinidin	-8.4
			Quercetin	-8.2
			Kaempferol	-8.0
			Morin	-7.9
2	ALB	1E7E	Betulinic acid	-9.6
3	CASP3	3GJQ	Beta-sitosterol	-9.8
			Ursolic acid	-9.1
			Oleanolic acid	-8.8
			Rutin	-8.8
			Rosmarinic acid	-8.2
			Quercetin	-7.7
			Morin	-7.6
4	CXCL8	5WDZ	Rutin	-6.9
			Quercetin	-6.4
5	EGF	2KV4	Quercetin	-6.9
6	IL10	1Y6K	Quercetin	-6.9
7	IL1B	5BVP	Ursolic acid	-7.6
			Rutin	-7.3
			Quercetin	-7.0
8	IL6	4O9H	Rutin	-7.3
			Ursolic acid	-7.2
			Quercetin	-7.1
9	INS	5USV	Rutin	-4.8
10	JUN	1JUN	Ursolic acid	-6.2
			Rosmarinic acid	-5.9
			Beta-sitosterol	-5.7
			Quercetin	-5.5
11	MAPK1	6RQ4	Quercetin	-9.2
			Caffeic acid	-6.5
12	MAPK3	2ZOQ	Maslinic acid	-9.4
			Corosolic acid	-9.1
			Ursolic acid	-9.0
			Spinasterol	-8.7
			Oleanolic acid	-8.6
			Stigmast-7-enol	-7.8
13	MMP9	6ESM	Luteolin	-10.8
			Quercetin	-10.7
			Morin	-10.6
			Kaempferol	-10.2
			Rosmarinic acid	-9.7
			Delphinidin	-8.9
			Caffeic acid	-8.0
			Ursolic acid	-7.8
14	PTGS2	5F19	Rutin	-9.7
			Luteolin	-9.6
			Quercetin	-9.6
			Kaempferol	-9.4
			Maslinic acid	-9.3
			Corosolic acid	-9.2
			Delphinidin	-9.2
			Oleanolic acid	-9.1
			Betulinic acid	-8.6
			Stigmasterol	-8.5
			Vulgaxanthin-I	-8.5
			Ursolic acid	-8.4
			Beta-sitosterol	-8.3
			Euscaphic acid	-8.3
			Caffeic acid	-7.3
15	STAT3	6NJS	Poriferasterol monoglucoside_qt	-8.1
			Ursolic acid	-7.9
			Caffeic acid	-6.1
16	TNF	5M2J	Rutin	-8.1
			Euscaphic acid	-8.0
			Quercetin	-8.0
			Ursolic acid	-7.9
			Rosmarinic acid	-7.5
			Caffeic acid	-6.2
17	TP53	6SI3	Quercetin	-8.0
			Ursolic acid	-7.7
18	VEGFA	1VPF	Ursolic acid	-8.2
			Delphinidin	-7.6
			Quercetin	-7.4

### Compound-target -pathway network

**Fig. 6:**
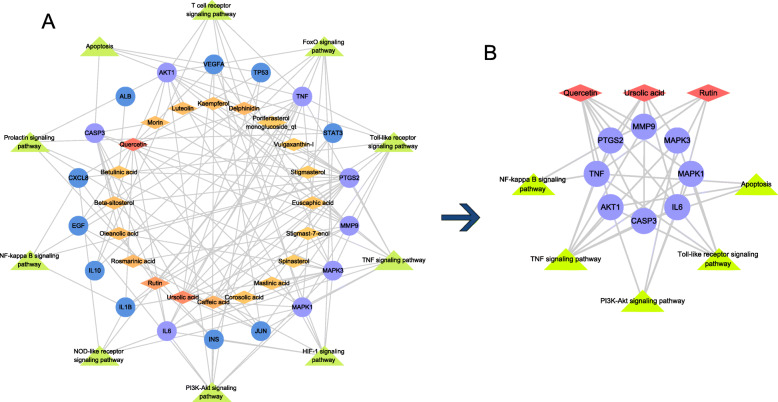
Compound-target-pathway network. **a** Compound-hub gene-pathway network. Diamond nodes represent compounds, the colors of which are proportional to the degree of nodes. Circular nodes represent hub genes, and 8 key targets are distinguished using lavender color. Triangle nodes represent pathways. **b** The sub-network is derived from (**a**) consisting of 3 compounds, 8 key targets and 4 pathways after network analysis

As shown in Fig. 6A, a compound-hub gene-pathway network was established by connecting potential pathways, compounds and hub genes. To find the major nodes, we took the average of respective degree as the threshold values to identify the significant compounds and targets. Three compounds and 8 targets were selected as core nodes in anti-TAO system: Quercetin (degree = 14), Ursolic acid (degree = 11) and Rutin (degree = 8), PTGS2 (degree = 17), MAPK3 (degree = 14), AKT1 (degree = 13), TNF (degree = 13), MAPK1 (degree = 10), CASP3 (degree = 9), IL6 (degree = 9) and MMP9 (degree = 9). Subsequently, for a more detailed explanation of the associations described above, we extracted a subnetwork to exhibit the core nodes and key mapping pathways (Fig. 6b).

## Discussion

In recent years, there has been growing researches devoted to drug discovery and combined treatment with TCM against complex disease such as TAO, in which the network pharmacology approach plays an essential role. In the present study, we constructed the following network to reveal the potential targets and pathways of SP in TAO treatment: compound-target network, PPI network of TAO targets, PPI network of compound-TAO targets and compound-target -pathway network.

After integrating and consolidating information from diverse sources of available databases, 20 active compounds of SP acted on 127 different targets associated with TAO. According to the compound-target network, six compounds and putative targets are highly connected and can be defined as vital compounds in SP. Previous studies have indicated the anti-inflammatory, pro-apoptosis and immunomodulation effects of Quercetin [55, 56], Ursolic acid [57–59], Luteolin [60–62], Kaempferol [63–65], Morin [66, 67] and Delphinidin [68, 69] in various diseases, suggesting SP may exhibit beneficial effects in TAO which is characterized by immunoinflammatory response. Actually, the PPI network of TAO targets did reflect the underlying pathogenesis of TAO, which are consistent with prior researches. Commonly reported mechanism [6] is the promotion and mutual interaction of these including that cellular immunity and humoral immunity mainly caused by massive activation of T lymphocytes, inflammatory response and proliferation of orbital fibroblasts. This manifests as an uncontrolled secretion of inflammatory cytokines and growth factors by activated orbital fibroblasts, leading to orbital tissue remodeling and enlargement. Further, to better elucidate the mechanism of SP in treating TAO, the 127 targets were screened for 18 hub genes in the PPI network of compound-TAO targets. Coincidentally, there are 6 targets considered as key pathogenic gene in the PPI network of TAO targets, which are involved in the 18 hub genes, namely TNF, ALB, IL6, INS, AKT1 and TP53, reconfirming SP might possess good effects on TAO.

According to the compound-hub gene-pathway network, Quercetin, Ursolic acid and Rutin interacted with the large number of targets, indicating the important roles in the anti-TAO system. Quercetin is a flavonoid phytoestrogen exhibiting antioxidant and anti-inflammatory properties and reducing proliferation in orbital fibroblasts [70–72]. In addition to the above effects, Ursolic acid [57–59] and Rutin [73, 74] are reported to promote apoptosis and regulate immune in other cell systems and animal models. Eight important therapeutic targets with the degree > 7.83 are PTGS2, MAPK3, AKT1, TNF, MAPK1, CASP3, IL6 and MMP9. Meanwhile, we performed molecular docking simulation between 18 hub genes and 20 active compounds to complement key targets. The results suggested that all of the target-compound pairs possess good docking affinity, especially, MMP9, PTGS2, CASP3 and ALB. ALB is the most abundant protein in human plasma, to which drugs usually bind, playing a crucial importance to understand the pharmacodynamics and pharmacokinetics [75]. Beyond that, the drop in ALB level reflects the oxidative stress damage [76], albeit not confirmed in TAO pathogenesis. We therefore speculate that the good affinity of ALB and Betulinic acid may refer to the general drug response rather than the treatment mechanism of SP. Consequently, SP plays a beneficial role in TAO, at least in part, through modulating PTGS2, MAPK3, AKT1, TNF, MAPK1, CASP3, IL6 and MMP9. GO functional annotation and pathway enrichment analysis further supported the potential therapeutic mechanism, the results of which was predominated by immune inflammation, proliferation and apoptosis.

In terms of immune inflammation, proinflammatory cytokine PTGS2, IL6 and TNF were validated the participation in TAO onset. PTGS2 is involved in prostaglandin biosynthesis, which have been ascribed key role in inflammation [77]. Prior studies have noted that PTGS2 diminished with a decrease in clinical activity score in TAO [78, 79], is currently believed to be pivotal to inflammatory process in TAO patients. IL6 has been implicated in the pathogenesis of autoimmune disease [80]. It has been reported that AKT1/NF-κB signaling pathway contributed to the production of IL6 in the retrobulbar space in active phase of TAO [81]. Likewise, research showed that the elevation levels of TNF in serum is mediated by AKT1/NF-κB signaling pathway in inflammatory response of TAO [82]. At present, there has been reported some inhibitors of TNF obtained promising results in patients with TAO, irrespective of rare adverse effects [83]. Then SP with TNF as a key therapeutic target may have potential to be an effective agent for TAO. Hence, it is possible that SP could suppress the inflammatory cytokines secretion of T lymphocytes and orbital fibroblasts through AKT1/NF-κB signaling pathway. Regarding whether SP could modulate immune like corticosteroids exerts [83], for instance, decrease antigen presentation to T lymphocytes in TAO, it requires further laboratory investigation.

**Fig. 7:**
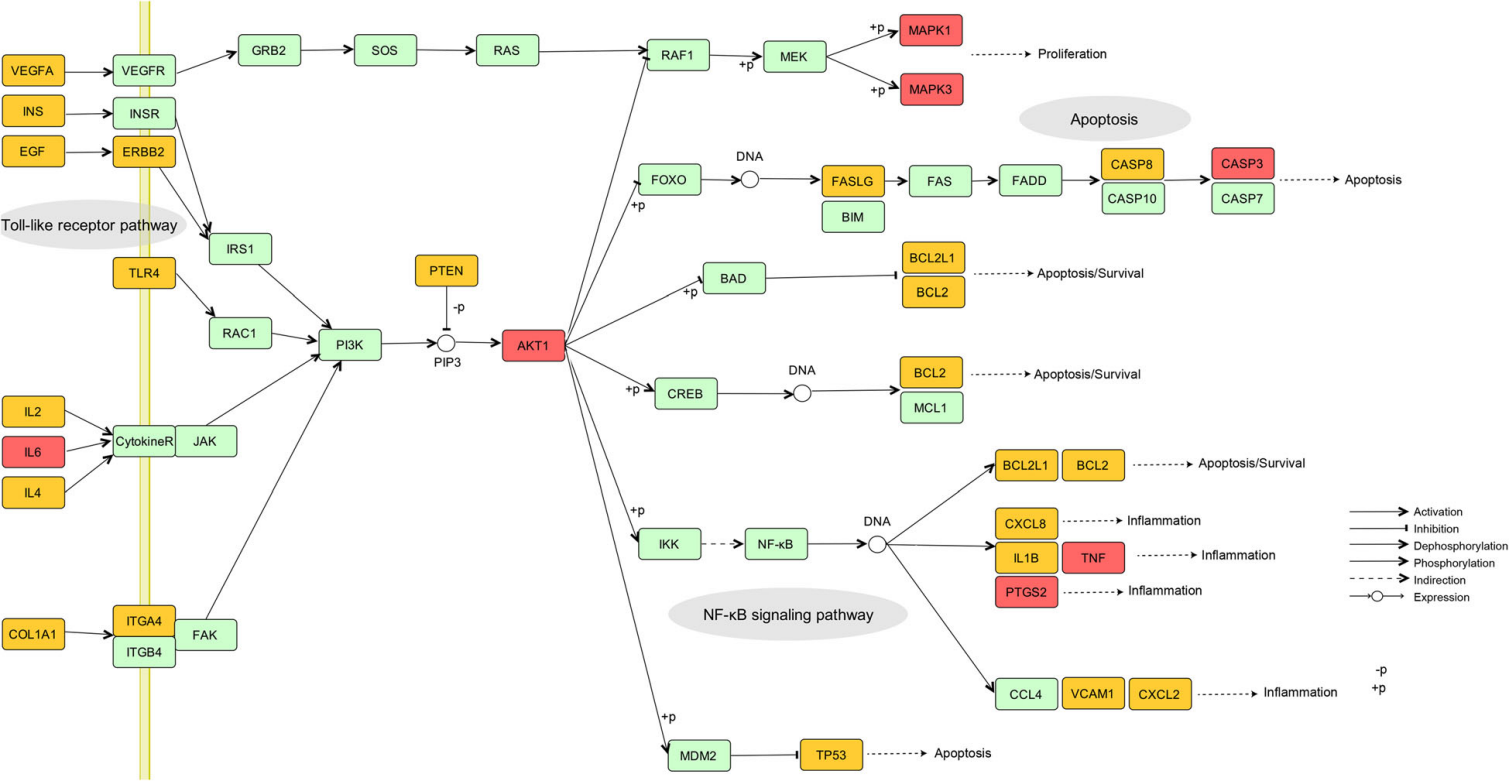
The PI3K-AKT signaling pathway plays a central role in anti-TAO system of SP. The red nodes represent key targets, the yellow nodes represent overlapping targets of SP and TAO targets, and the green nodes represent the other targets in PI3K-AKT signaling pathway. Grey ellipses represent important pathways associated with TAO and mediated by PI3K-AKT pathway as well

For proliferation and apoptosis, PI3K-AKT signaling pathway seems to be significant in mediating cell growth and death in TAO. Recently studies demonstrated that orbital fibrocytes express thyrotropin receptor (TSHR) [84] and activation of TSHR leads to increased inflammatory gene expression and proliferation through PI3K-AKT pathway [85–87]. Besides this, the increased cell proliferation of orbital preadipocytes is also contributed in TAO progression [88]. CASP3 activation is one of the final steps in the execution of apoptosis, which is activated in orbital preadipocytes by some compounds in TAO [88]. And SP exhibits pro-apoptotic actions via activation of CASP3 in various cancer [89]. Although there is a lack of relevant studies of MAPK1, MAPK3 in TAO, they act in a signaling cascade that regulates various cellular processes such as proliferation and apoptosis [90]. MMP9 as a kind of matrix metalloproteinases has been proved to participate in inflammation of multiple tumors through TNF signaling pathway [91]. However, it still requires further researches to elucidate the role of MMP9 in TAO pathogenesis. Therefore, it can be summarized from the results that whether in immune inflammation or proliferation and apoptosis, the PI3K-AKT signaling pathway plays a key role in TAO, and inhibition of this process could be an effective therapeutic target for SP against TAO (Fig. 7).

## Conclusion

In conclusion, up till now, TAO treatments have been an intractable problem, but TCM may be a potential alternative treatment to a certain extent. In the present study, we predicted 8 key targets from complex networks through the integration of network pharmacology and molecular docking, and performed comprehensive explanation of the therapeutic mechanism of SP in TAO, which is likely to focus on three aspects: immunoinflammatory suppression and regulation of proliferation and apoptosis. In addition, we found that PI3K-AKT signaling pathway may occupy core status in anti-TAO system. Our research would be enlightening for developing therapeutic methods targeted TAO. However, further extensive experiments will be necessary to unravel the above possibility, such as in vivo and in vitro efficacy experiments of SP in TAO therapy.

## Supplementary information

**Additional file 1: Table S1** The putative targets of SP.

**Additional file 2: Table S2** TAO related targets

**Additional file 3: Table S3** Information for TAO targets after PPI analysis.

**Additional file 4: Table S4** Information for overlapped targets after PPI analysis.

## Data Availability

All data generated or analyzed during this study are available from public databases, published articles and supplementary materials.

## Abbreviations

ALA: Alanine
ARG: Arginine
BP: Biological processes
CC: Cellular component
CNKI: China National Knowledge Infrastructure Database
DL: Drug-likeness
GLN: Glutamine
GLY: Glycine
GO: Gene Ontology
HPLC: High-performance liquid chromatography
KEGG: Kyoto Encyclopedia of Genes and Genomes
LEU: Leucine
MET: Methionine
MF: Molecular function
OB: Oral bioavailability
PDB: Protein Data Bank
PPI: Protein-protein interaction
PRO: Proline
SP: Spica Prunellae
TAO: Thyroid-associated ophthalmopathy
TCM: Traditional Chinese medicine
TSHR: Thyrotropin receptor
TYR: Tyrosine
VAL: Valine
