# Supplementary material for: Mechanisms of Spica Prunellae against thyroid-associated Ophthalmopathy based on network pharmacology and molecular docking
Source: BMC Complement Med Ther. 2020 Jul 20;20:229. doi: 10.1186/s12906-020-03022-2 (PMC7372882; doi:10.1186/s12906-020-03022-2)
Supplement: Supplementary file 1 — Additional file 1: Table S1 The putative targets of SP. [file 12906_2020_3022_MOESM1_ESM.docx]

**Table S1** The putative targets of SP.

| **Number** | **Gene name** | **Uniprot ID** | **Protein name** | **Database** |
| --- | --- | --- | --- | --- |
| 1 | ABCB1 | P08183 | ATP binding cassette subfamily B member 1 | SwissTargetPrediction/Pubchem/STITCH |
| 2 | ABCB11 | O95342 | Bile salt export pump | STITCH |
| 3 | ABCC1 | P33527 | Multidrug resistance-associated protein 1 | SwissTargetPrediction/Pubchem/STITCH |
| 4 | ABCG2 | Q9UNQ0 | ATP-binding cassette sub-family G member 2 | TCMSP/SwissTargetPrediction/Pubchem |
| 5 | ACACA | Q13085 | Acetyl-CoA carboxylase 1 | TCMSP/STITCH |
| 6 | ACHE | P22303 | Acetylcholinesterase | TCMSP/SwissTargetPrediction/Pubchem |
| 7 | ACP1 | P24666 | Low molecular weight phosphotyrosine protein phosphatase | SwissTargetPrediction/Pubchem |
| 8 | ACPP | P15309 | Prostatic acid phosphatase | TCMSP |
| 9 | ADAM10 | O14672 | ADAM metallopeptidase domain 10 | Pubchem |
| 10 | ADAM17 | P78536 | ADAM metallopeptidase domain 17 | Pubchem |
| 11 | ADH1C | P00326 | Alcohol dehydrogenase 1C | TCMSP |
| 12 | ADORA1 | P30542 | Adenosine A1 receptor (by homology) | SwissTargetPrediction |
| 13 | ADORA2A | P29274 | Adenosine A2a receptor (by homology) | SwissTargetPrediction/Pubchem |
| 14 | ADORA3 | P0DMS8 | Adenosine A3 receptor | SwissTargetPrediction/Pubchem |
| 15 | ADRA1A | P35348 | Alpha-1A adrenergic receptor | TCMSP |
| 16 | ADRA1B | P35368 | Alpha-1B adrenergic receptor | TCMSP |
| 17 | ADRA1D | P25100 | Alpha-1D adrenergic receptor | TCMSP |
| 18 | ADRA2A | P08913 | Alpha-2A adrenergic receptor | TCMSP/SwissTargetPrediction/Pubchem |
| 19 | ADRA2C | P18825 | Alpha-2C adrenergic receptor | TCMSP/SwissTargetPrediction/Pubchem |
| 20 | ADRB1 | P08588 | Beta-1 adrenergic receptor | TCMSP |
| 21 | ADRB2 | P07550 | Beta-2 adrenergic receptor | TCMSP |
| 22 | AGTR1 | P30556 | Type-1 angiotensin II receptor | SwissTargetPrediction |
| 23 | AHR | P35869 | Aryl hydrocarbon receptor | TCMSP/SwissTargetPrediction/Pubchem/STITCH |
| 24 | AHSA1 | O95433 | Activator of 90 kDa heat shock protein ATPase homolog 1 | TCMSP |
| 25 | AKR1A1 | P14550 | Aldehyde reductase (by homology) | SwissTargetPrediction |
| 26 | AKR1B1 | P15121 | Aldose reductase | TCMSP/SwissTargetPrediction/Pubchem |
| 27 | AKR1B10 | O60218 | Aldo-keto reductase family 1 member B10 | SwissTargetPrediction/Pubchem |
| 28 | AKR1C1 | Q04828 | Aldo-keto reductase family 1 member C1 (by homology) | SwissTargetPrediction |
| 29 | AKR1C2 | P52895 | Aldo-keto reductase family 1 member C2 | SwissTargetPrediction |
| 30 | AKR1C3 | P42330 | Aldo-keto-reductase family 1 member C3 | SwissTargetPrediction/STITCH |
| 31 | AKR1C4 | P17516 | Aldo-keto reductase family 1 member C4 | SwissTargetPrediction |
| 32 | AKT1 | P31749 | RAC-alpha serine/threonine-protein kinase | TCMSP/SwissTargetPrediction/Pubchem/STITCH |
| 33 | ALB | P02768 | albumin | Pubchem |
| 34 | ALDH1A1 | P00352 | aldehyde dehydrogenase 1 family member A1 | Pubchem |
| 35 | ALDH2 | P05091 | Aldehyde dehydrogenase | SwissTargetPrediction/STITCH |
| 36 | ALK | Q9UM73 | ALK tyrosine kinase receptor | SwissTargetPrediction/Pubchem |
| 37 | ALOX12 | P18054 | Arachidonate 12-lipoxygenase | SwissTargetPrediction/Pubchem |
| 38 | ALOX15 | P16050 | Arachidonate 15-lipoxygenase | SwissTargetPrediction/Pubchem |
| 39 | ALOX15B | O15296 | arachidonate 15-lipoxygenase type B | Pubchem |
| 40 | ALOX5 | P09917 | Arachidonate 5-lipoxygenase | TCMSP/SwissTargetPrediction/Pubchem |
| 41 | ALOX5AP | P20292 | 5-lipoxygenase activating protein | SwissTargetPrediction |
| 42 | ALPI | P09923 | alkaline phosphatase, intestinal | Pubchem |
| 43 | ALPL | P05186 | alkaline phosphatase, biomineralization associated | Pubchem |
| 44 | AMPD2 | Q01433 | AMP deaminase 2 | SwissTargetPrediction |
| 45 | AMY1A | P04745 | AMY1C | SwissTargetPrediction/Pubchem |
| 46 | AMY2A | P04746 | Pancreatic alpha-amylase | TCMSP |
| 47 | APEX1 | P27695 | DNA-(apurinic or apyrimidinic site) lyase | SwissTargetPrediction |
| 48 | APH1A | Q96BI3 | Gamma-secretase subunit APH-1A | SwissTargetPrediction |
| 49 | APH1B | Q8WW43 | Gamma-secretase subunit APH-1B | SwissTargetPrediction |
| 50 | APP | P05067 | Beta amyloid A4 protein | SwissTargetPrediction/Pubchem |
| 51 | AR | P10275 | Androgen receptor | TCMSP/SwissTargetPrediction/Pubchem |
| 52 | ARG1 | P05089 | Arginase-1 (by homology) | SwissTargetPrediction |
| 53 | ASNA1 | O43681 | ATPase ASNA1 | STITCH |
| 54 | ATAD5 | Q96QE3 | ATPase family AAA domain containing 5 | Pubchem |
| 55 | ATF2 | P15336 | Cyclic AMP-dependent transcription factor ATF-2 | TCMSP |
| 56 | ATP12A | P54707 | Potassium-transporting ATPase alpha chain 2 | SwissTargetPrediction |
| 57 | ATP5B | Q0QEN7 | ATP synthase subunit beta | STITCH |
| 58 | AURKB | Q96GD4 | Serine/threonine-protein kinase Aurora-B | SwissTargetPrediction/Pubchem |
| 59 | AVPR2 | P30518 | Vasopressin V2 receptor | SwissTargetPrediction/Pubchem |
| 60 | AXL | P30530 | Tyrosine-protein kinase receptor UFO | SwissTargetPrediction/Pubchem |
| 61 | BACE1 | P56817 | Beta-secretase 1 | SwissTargetPrediction/Pubchem |
| 62 | BAK1 | Q16611 | Bcl-2 homologous antagonist/killer | STITCH |
| 63 | BAP1 | Q92560 | BRCA1 associated protein 1 | Pubchem |
| 64 | BAX | Q07812 | Apoptosis regulator BAX | TCMSP |
| 65 | BAZ2B | Q9UIF8 | bromodomain adjacent to zinc finger domain 2B | Pubchem |
| 66 | BCHE | P06276 | Butyrylcholinesterase | SwissTargetPrediction/Pubchem |
| 67 | BCL2 | P10415 | Apoptosis regulator Bcl-2 | TCMSP |
| 68 | BCL2L1 | Q07817 | Bcl-2-like protein 1 | TCMSP |
| 69 | BIRC5 | O15392 | Baculoviral IAP repeat-containing protein 5 | TCMSP |
| 70 | BLM | P54132 | BLM RecQ like helicase | Pubchem |
| 71 | BTK | Q06187 | Tyrosine-protein kinase BTK | TCMSP |
| 72 | C3 | P01024 | Complement C3 | TCMSP |
| 73 | C5 | P01031 | Complement C5 | TCMSP |
| 74 | C5AR1 | P21730 | C5a anaphylatoxin chemotactic receptor | TCMSP |
| 75 | CA1 | P00915 | Carbonic anhydrase I | SwissTargetPrediction/Pubchem |
| 76 | CA12 | O43570 | Carbonic anhydrase XII | SwissTargetPrediction/Pubchem |
| 77 | CA13 | Q8N1Q1 | Carbonic anhydrase XIII | SwissTargetPrediction |
| 78 | CA14 | Q9ULX7 | Carbonic anhydrase XIV | SwissTargetPrediction/Pubchem |
| 79 | CA2 | P00918 | Carbonic anhydrase II | TCMSP/SwissTargetPrediction/Pubchem |
| 80 | CA3 | P07451 | Carbonic anhydrase III | SwissTargetPrediction/Pubchem |
| 81 | CA4 | P22748 | Carbonic anhydrase IV | SwissTargetPrediction/Pubchem |
| 82 | CA5A | P35218 | Carbonic anhydrase VA | SwissTargetPrediction/Pubchem |
| 83 | CA5B | Q9Y2D0 | Carbonic anhydrase VB | SwissTargetPrediction/Pubchem |
| 84 | CA6 | P23280 | Carbonic anhydrase VI | SwissTargetPrediction/Pubchem |
| 85 | CA7 | P43166 | Carbonic anhydrase VII | SwissTargetPrediction/Pubchem |
| 86 | CA9 | Q16790 | Carbonic anhydrase IX | SwissTargetPrediction/Pubchem |
| 87 | CALM1 | P0DP23 | calmodulin 1 | Pubchem |
| 88 | CAMK2B | Q13554 | CaM kinase II beta | SwissTargetPrediction/Pubchem |
| 89 | CASP1 | P29466 | Caspase-1 | TCMSP |
| 90 | CASP3 | P42574 | Caspase-3 | TCMSP/SwissTargetPrediction/STITCH |
| 91 | CASP7 | P55210 | Caspase-7 | STITCH |
| 92 | CASP8 | Q14790 | Caspase-8 | TCMSP/STITCH |
| 93 | CASP9 | P55211 | Caspase-9 | TCMSP/STITCH |
| 94 | CAT | P04040 | Catalase | TCMSP/STITCH |
| 95 | CAV1 | Q03135 | Caveolin-1 | TCMSP |
| 96 | CBR3 | O75828 | carbonyl reductase 3 | Pubchem |
| 97 | CBX1 | P83916 | chromobox 1 | Pubchem |
| 98 | CCL11 | P51671 | Eotaxin | TCMSP |
| 99 | CCL2 | P13500 | C-C motif chemokine 2 | TCMSP |
| 100 | CCL3 | P10147 | C-C motif chemokine 3 | TCMSP |
| 101 | CCN2 | P29279 | CCN family member 2 | STITCH |
| 102 | CCNB1 | P14635 | G2/mitotic-specific cyclin-B1 | TCMSP/SwissTargetPrediction/Pubchem |
| 103 | CCNB2 | O95067 | G2/mitotic-specific cyclin-B2 | SwissTargetPrediction/Pubchem |
| 104 | CCNB3 | Q8WWL7 | G2/mitotic-specific cyclin-B3 | SwissTargetPrediction/Pubchem |
| 105 | CCND1 | P24385 | G1/S-specific cyclin-D1 | TCMSP |
| 106 | CCND2 | P30279 | G1/S-specific cyclin-D2 | TCMSP |
| 107 | CCND3 | P30281 | G1/S-specific cyclin-D3 | TCMSP |
| 108 | CCR1 | P32246 | C-C chemokine receptor type 1 | SwissTargetPrediction |
| 109 | CCR3 | P51677 | C-C chemokine receptor type 3 | TCMSP |
| 110 | CCR4 | P51679 | C-C motif chemokine receptor 4 | Pubchem |
| 111 | CD247 | P20963 | T-cell surface glycoprotein CD3 zeta chain | TCMSP |
| 112 | CD38 | P28907 | Lymphocyte differentiation antigen CD38 | SwissTargetPrediction/Pubchem |
| 113 | CD40LG | P29965 | CD40 ligand | TCMSP |
| 114 | CD80 | P33681 | T-lymphocyte activation antigen CD80 | TCMSP |
| 115 | CD81 | P60033 | CD81 antigen | SwissTargetPrediction |
| 116 | CD86 | P42081 | T-lymphocyte activation antigen CD86 | TCMSP |
| 117 | CDC25A | P30304 | Dual specificity phosphatase Cdc25A | SwissTargetPrediction |
| 118 | CDC25B | P30305 | Dual specificity phosphatase Cdc25B | SwissTargetPrediction/Pubchem |
| 119 | CDC25C | P30307 | Dual specificity phosphatase Cdc25C | SwissTargetPrediction |
| 120 | CDH1 | P12830 | Cadherin-1 | STITCH |
| 121 | CDK1 | P06493 | Cell division control protein 2 homolog | TCMSP/SwissTargetPrediction/Pubchem/STITCH |
| 122 | CDK2 | P24941 | Cyclin-dependent kinase 2 | SwissTargetPrediction/Pubchem |
| 123 | CDK4 | P11802 | Cell division protein kinase 4 | TCMSP |
| 124 | CDK5 | Q00535 | cyclin dependent kinase 5 | Pubchem |
| 125 | CDK5R1 | Q15078 | Cyclin-dependent kinase 5/CDK5 activator 1 | SwissTargetPrediction/Pubchem |
| 126 | CDK6 | Q00534 | Cell division protein kinase 6 | TCMSP/SwissTargetPrediction/Pubchem |
| 127 | CDKN1A | P38936 | Cyclin-dependent kinase inhibitor 1 | TCMSP |
| 128 | CDKN2A | P42771 | Cyclin-dependent kinase inhibitor 2A, isoforms 1/2/3 | TCMSP |
| 129 | CES1 | P23141 | Acyl coenzyme A:cholesterol acyltransferase | SwissTargetPrediction |
| 130 | CES2 | O00748 | Carboxylesterase 2 | SwissTargetPrediction |
| 131 | CFTR | P13569 | Cystic fibrosis transmembrane conductance regulator | SwissTargetPrediction/Pubchem |
| 132 | CHEK2 | O96017 | Serine/threonine-protein kinase Chk2 | TCMSP |
| 133 | CHRM1 | P11229 | Muscarinic acetylcholine receptor M1 | TCMSP/Pubchem |
| 134 | CHRM2 | P08172 | Muscarinic acetylcholine receptor M2 | TCMSP/SwissTargetPrediction |
| 135 | CHRM3 | P20309 | Muscarinic acetylcholine receptor M3 | TCMSP |
| 136 | CHRM4 | [P08173](https://www.uniprot.org/uniprot/P08173) | Muscarinic acetylcholine receptor M4 | TCMSP |
| 137 | CHRNA2 | Q15822 | Neuronal acetylcholine receptor subunit alpha-2 | TCMSP |
| 138 | CHRNA7 | P36544 | Neuronal acetylcholine receptor protein, alpha-7 chain | TCMSP |
| 139 | CHUK | O15111 | Inhibitor of nuclear factor kappa-B kinase subunit alpha | TCMSP |
| 140 | CISD1 | Q9NZ45 | CDGSH iron sulfur domain 1 | Pubchem |
| 141 | CLDN4 | O14493 | Claudin-4 | TCMSP |
| 142 | CLK1 | P49759 | CDC like kinase 1 | Pubchem |
| 143 | CLK3 | P49761 | CDC like kinase 3 | Pubchem |
| 144 | CNR1 | P21554 | Cannabinoid receptor 1 | SwissTargetPrediction |
| 145 | COL1A1 | P02452 | Collagen alpha-1(I) chain | TCMSP |
| 146 | COL3A1 | P02461 | Collagen alpha-1(III) chain | TCMSP |
| 147 | COMT | P21964 | Catechol O-methyltransferase | STITCH |
| 148 | CREB1 | P16220 | Cyclic AMP-responsive element-binding protein 1 | TCMSP |
| 149 | CRP | P02741 | C-reactive protein | TCMSP |
| 150 | CSF2 | P04141 | Granulocyte-macrophage colony-stimulating factor | TCMSP |
| 151 | CSNK1G1 | Q9HCP0 | casein kinase 1 gamma 1 | Pubchem |
| 152 | CSNK1G2 | P78368 | casein kinase 1 gamma 2 | Pubchem |
| 153 | CSNK2A1 | P68400 | Casein kinase II alpha | SwissTargetPrediction/Pubchem |
| 154 | CSNK2A2 | P19784 | casein kinase 2 alpha 2 | Pubchem |
| 155 | CSNK2B | P67870 | casein kinase 2 beta | Pubchem |
| 156 | CTBP2 | P56545 | C-terminal-binding protein 2 | SwissTargetPrediction |
| 157 | CTDSP1 | Q9GZU7 | CTD small phosphatase 1 | Pubchem |
| 158 | CTNNB1 | P35222 | Catenin beta-1 | STITCH |
| 159 | CTRB1 | P17538 | Chymotrypsinogen B | TCMSP |
| 160 | CTSD | P07339 | Cathepsin D | TCMSP/Pubchem |
| 161 | CXCL10 | P02778 | C-X-C motif chemokine 10 | TCMSP |
| 162 | CXCL11 | O14625 | C-X-C motif chemokine 11 | TCMSP |
| 163 | CXCL2 | P19875 | C-X-C motif chemokine 2 | TCMSP |
| 164 | CXCL8 | P10145 | Interleukin-8 | TCMSP |
| 165 | CXCR1 | P25024 | Interleukin-8 receptor A | SwissTargetPrediction/Pubchem |
| 166 | CYBB | P04839 | cytochrome b-245 beta chain | Pubchem |
| 167 | CYP17A1 | P05093 | Cytochrome P450 17A1 | SwissTargetPrediction |
| 168 | CYP19A1 | P11511 | Cytochrome P450 19A1 | SwissTargetPrediction/Pubchem |
| 169 | CYP1A1 | P04798 | Cytochrome P450 1A1 | TCMSP/STITCH/Pubchem |
| 170 | CYP1A2 | P05177 | Cytochrome P450 1A2 | TCMSP/SwissTargetPrediction/Pubchem |
| 171 | CYP1B1 | Q16678 | Cytochrome P450 1B1 | SwissTargetPrediction/STITCH/Pubchem |
| 172 | CYP2C19 | P33261 | Cytochrome P450 2C19 | SwissTargetPrediction/Pubchem/STITCH |
| 173 | CYP2C8 | P10632 | cytochrome P450 family 2 subfamily C member 8 | Pubchem/STITCH |
| 174 | CYP2C9 | P11712 | Cytochrome P450 2C9 | SwissTargetPrediction/Pubchem |
| 175 | CYP2D6 | P10635 | cytochrome P450 family 2 subfamily D member 6 | Pubchem |
| 176 | CYP3A4 | P08684 | Cytochrome P450 3A4 | TCMSP/SwissTargetPrediction/Pubchem/STITCH |
| 177 | CYP51A1 | Q16850 | Cytochrome P450 51 (by homology) | SwissTargetPrediction |
| 178 | CYSLTR1 | Q9Y271 | Cysteinyl leukotriene receptor 1 | SwissTargetPrediction |
| 179 | DAPK1 | P53355 | Death-associated protein kinase 1 | SwissTargetPrediction/Pubchem |
| 180 | DCAF5 | Q96JK2 | DDB1- and CUL4-associated factor 5 | TCMSP |
| 181 | DHCR7 | Q9UBM7 | Anti-estrogen binding site (AEBS) (by homology) | SwissTargetPrediction |
| 182 | DHODH | Q02127 | Dihydroorotate dehydrogenase | SwissTargetPrediction |
| 183 | DIO1 | P49895 | Type I iodothyronine deiodinase | TCMSP |
| 184 | DIRAS1 | O95057 | DIRAS family GTPase 1 | Pubchem |
| 185 | DLD | P09622 | dihydrolipoamide dehydrogenase | Pubchem |
| 186 | DNMT1 | P26358 | DNA methyltransferase 1 | Pubchem |
| 187 | DPP4 | P27487 | Dipeptidyl peptidase IV | TCMSP/Pubchem |
| 188 | DRD1 | P21728 | Dopamine D1 receptor | TCMSP/Pubchem |
| 189 | DRD2 | P14416 | dopamine receptor D2 | Pubchem |
| 190 | DRD3 | P35462 | dopamine receptor D3 | Pubchem |
| 191 | DRD4 | P21917 | Dopamine D4 receptor | SwissTargetPrediction/Pubchem |
| 192 | DUOX2 | Q9NRD8 | Dual oxidase 2 | TCMSP |
| 193 | E2F1 | Q01094 | Transcription factor E2F1 | TCMSP |
| 194 | E2F2 | Q14209 | Transcription factor E2F2 | TCMSP |
| 195 | E6 | P03126 | protein E6*;transforming protein E6 | Pubchem |
| 196 | EDNRA | P25101 | Endothelin receptor ET-A | SwissTargetPrediction |
| 197 | EDNRB | P24530 | Endothelin receptor ET-B | SwissTargetPrediction |
| 198 | EGF | P01133 | Pro-epidermal growth factor | TCMSP |
| 199 | EGFR | P00533 | Epidermal growth factor receptor | TCMSP/SwissTargetPrediction/Pubchem/STITCH |
| 200 | EHMT2 | Q96KQ7 | Histone-lysine N-methyltransferase EHMT2 | Pubchem |
| 201 | EIF4H | Q15056 | eukaryotic translation initiation factor 4H | Pubchem |
| 202 | EIF6 | P56537 | Eukaryotic translation initiation factor 6 | TCMSP |
| 203 | ELANE | P08246 | Leukocyte elastase | SwissTargetPrediction |
| 204 | ELK1 | P19419 | ETS domain-containing protein Elk-1 | TCMSP |
| 205 | ENPP7 | Q6UWV6 | Ectonucleotide pyrophosphatase/phosphodiesterase family member 7 | TCMSP |
| 206 | EPHX2 | P34913 | epoxide hydrolase 2 | Pubchem |
| 207 | ERBB2 | P04626 | Receptor tyrosine-protein kinase erbB-2 | TCMSP/SwissTargetPrediction |
| 208 | ERBB3 | P21860 | Receptor tyrosine-protein kinase erbB-3 | TCMSP |
| 209 | ERN1 | O75460 | Serine/threonine-protein kinase/endoribonuclease IRE1 | STITCH |
| 210 | ESR1 | P03372 | Estrogen receptor alpha | SwissTargetPrediction/Pubchem |
| 211 | ESR2 | Q92731 | Estrogen receptor beta | SwissTargetPrediction/Pubchem |
| 212 | ESRRA | P11474 | Estrogen-related receptor alpha | SwissTargetPrediction/Pubchem |
| 213 | EYA2 | O00167 | EYA transcriptional coactivator and phosphatase 2 | Pubchem |
| 214 | EZH2 | Q15910 | enhancer of zeste 2 polycomb repressive complex 2 subunit | Pubchem |
| 215 | F10 | P00742 | Coagulation factor Xa | TCMSP |
| 216 | F2 | P00734 | Prothrombin | TCMSP/SwissTargetPrediction/Pubchem |
| 217 | F3 | P13726 | Tissue factor | TCMSP/SwissTargetPrediction |
| 218 | F7 | P08709 | Coagulation factor VII | TCMSP/SwissTargetPrediction |
| 219 | FAAH | O00519 | Anandamide amidohydrolase | SwissTargetPrediction |
| 220 | FABP1 | P07148 | Fatty acid-binding protein, liver | SwissTargetPrediction |
| 221 | FABP2 | P12104 | Fatty acid binding protein intestinal | SwissTargetPrediction |
| 222 | FABP3 | P05413 | Fatty acid binding protein muscle | SwissTargetPrediction |
| 223 | FABP4 | P15090 | Fatty acid binding protein adipocyte | SwissTargetPrediction |
| 224 | FABP5 | Q01469 | Fatty acid binding protein epidermal | SwissTargetPrediction |
| 225 | FASLG | P48023 | Tumor necrosis factor ligand superfamily member 6 | TCMSP |
| 226 | FASN | P49327 | Fatty acid synthase | TCMSP |
| 227 | FCER2 | P06734 | Low affinity immunoglobulin epsilon Fc receptor | TCMSP |
| 228 | FDFT1 | P37268 | Squalene synthetase | SwissTargetPrediction |
| 229 | FEN1 | P39748 | flap structure-specific endonuclease 1 | Pubchem |
| 230 | FFAR1 | O14842 | Free fatty acid receptor 1 | SwissTargetPrediction |
| 231 | FGF2 | P09038 | Heparin-binding growth factor 2 | TCMSP/STITCH |
| 232 | FLT1 | P17948 | Vascular endothelial growth factor receptor 1 | SwissTargetPrediction |
| 233 | FLT3 | P36888 | Tyrosine-protein kinase receptor FLT3 | SwissTargetPrediction/Pubchem |
| 234 | FNTA | P49354 | Protein farnesyltransferase/geranylgeranyltransferase type-1 subunit alpha | SwissTargetPrediction |
| 235 | FNTB | P49356 | Protein farnesyltransferase subunit beta, FTase-beta | SwissTargetPrediction |
| 236 | FOS | P01100 | Proto-oncogene c-Fos | TCMSP |
| 237 | FTO | Q9C0B1 | Alpha-ketoglutarate-dependent dioxygenase FTO | SwissTargetPrediction |
| 238 | FXN | Q16595 | frataxin | Pubchem |
| 239 | FYN | P06241 | Tyrosine-protein kinase FYN | SwissTargetPrediction/Pubchem/STITCH |
| 240 | G6PD | P11413 | Glucose-6-phosphate 1-dehydrogenase | SwissTargetPrediction |
| 241 | GAA | P10253 | glucosidase alpha, acid | Pubchem |
| 242 | GABBR1 | Q9UBS5 | GABA-B receptor (by homology) | SwissTargetPrediction |
| 243 | GABRA1 | P14867 | Gamma-aminobutyric acid receptor subunit alpha-1 | TCMSP |
| 244 | GABRA2 | P47869 | Gamma-aminobutyric-acid receptor alpha-2 subunit | TCMSP/SwissTargetPrediction |
| 245 | GABRA3 | P34903 | Gamma-aminobutyric-acid receptor alpha-3 subunit | TCMSP |
| 246 | GABRA5 | P31644 | Gamma-aminobutyric-acid receptor alpha-5 subunit | TCMSP |
| 247 | GABRB2 | P47870 | GABA A receptor beta-2 | SwissTargetPrediction |
| 248 | GABRG2 | P18507 | GABA A receptor gamma-2 | SwissTargetPrediction |
| 249 | GALK1 | P51570 | galactokinase 1 | Pubchem |
| 250 | GAP43 | P17677 | Neuromodulin | TCMSP |
| 251 | GAPDH | P04406 | glyceraldehyde-3-phosphate dehydrogenase | Pubchem |
| 252 | GCK | P35557 | glucokinase | Pubchem |
| 253 | GFAP | P14136 | Glial fibrillary acidic protein | TCMSP |
| 254 | GFER | P55789 | growth factor, augmenter of liver regeneration | Pubchem |
| 255 | GJA1 | P17302 | Gap junction alpha-1 protein | TCMSP |
| 256 | GLI1 | P08151 | GLI family zinc finger 1 | Pubchem |
| 257 | GLI3 | P10071 | GLI family zinc finger 3 | Pubchem |
| 258 | GLO1 | Q04760 | Glyoxalase I | SwissTargetPrediction/Pubchem |
| 259 | GLRA1 | P23415 | Glycine receptor subunit alpha-1 | SwissTargetPrediction |
| 260 | GLS | O94925 | glutaminase | Pubchem |
| 261 | GNAI1 | P63096 | G protein subunit alpha i1 | Pubchem |
| 262 | GPBAR1 | Q8TDU6 | G-protein coupled bile acid receptor 1 | SwissTargetPrediction/Pubchem |
| 263 | GPR35 | Q9HC97 | G-protein coupled receptor 35 | SwissTargetPrediction/Pubchem |
| 264 | GRIK1 | P39086 | Glutamate receptor ionotropic kainate 1 | SwissTargetPrediction |
| 265 | GRIK2 | Q13002 | Glutamate receptor ionotropic kainate 2 | SwissTargetPrediction |
| 266 | GRIN2A | Q12879 | Cathepsin B | TCMSP |
| 267 | GRK6 | P43250 | G protein-coupled receptor kinase 6 | SwissTargetPrediction |
| 268 | GRM2 | Q14416 | Metabotropic glutamate receptor 2 (by homology) | SwissTargetPrediction |
| 269 | GSK3A | P49840 | glycogen synthase kinase 3 alpha | Pubchem |
| 270 | GSK3B | P49841 | Glycogen synthase kinase-3 beta | SwissTargetPrediction/Pubchem |
| 271 | GSR | P00390 | Glutathione reductase, mitochondrial | STITCH |
| 272 | GSTM1 | P09488 | Glutathione S-transferase Mu 1 | TCMSP |
| 273 | GSTM2 | P28161 | Glutathione S-transferase Mu 2 | TCMSP |
| 274 | GSTO1 | P78417 | glutathione S-transferase omega 1 | Pubchem |
| 275 | GSTP1 | P09211 | Glutathione S-transferase P | TCMSP |
| 276 | HAS2 | Q92819 | Hyaluronan synthase 2 | TCMSP |
| 277 | HCAR2 | Q8TDS4 | Hydroxycarboxylic acid receptor 2 | SwissTargetPrediction |
| 278 | HCK | P08631 | Tyrosine-protein kinase HCK | STITCH |
| 279 | HDAC1 | Q13547 | histone deacetylase 1 | Pubchem |
| 280 | HDAC10 | Q969S8 | histone deacetylase 10 | Pubchem |
| 281 | HDAC11 | Q96DB2 | histone deacetylase 11 | Pubchem |
| 282 | HDAC2 | Q92769 | histone deacetylase 2 | Pubchem |
| 283 | HDAC3 | O15379 | histone deacetylase 3 | Pubchem |
| 284 | HDAC4 | P56524 | histone deacetylase 4 | Pubchem |
| 285 | HDAC5 | Q9UQL6 | histone deacetylase 5 | Pubchem |
| 286 | HDAC6 | Q9UBN7 | histone deacetylase 6 | Pubchem |
| 287 | HDAC7 | Q8WUI4 | histone deacetylase 7 | Pubchem |
| 288 | HDAC8 | Q9BY41 | histone deacetylase 8 | Pubchem |
| 289 | HDAC9 | Q9UKV0 | histone deacetylase 9 | Pubchem |
| 290 | HERC5 | Q9UII4 | Probable E3 ubiquitin-protein ligase HERC5 | TCMSP |
| 291 | HIBCH | Q6NVY1 | 3-hydroxyisobutyryl-CoA hydrolase, mitochondrial | STITCH |
| 292 | HIF1A | Q16665 | Hypoxia-inducible factor 1-alpha | TCMSP/Pubchem |
| 293 | HIF1AN | Q9NWT6 | hypoxia inducible factor 1 subunit alpha inhibitor | Pubchem |
| 294 | HK2 | P52789 | Hexokinase-2 | TCMSP |
| 295 | HMGCR | P04035 | 3-hydroxy-3-methylglutaryl-coenzyme A reductase | TCMSP/SwissTargetPrediction |
| 296 | HMOX1 | P09601 | Heme oxygenase 1 | TCMSP/STITCH |
| 297 | HPGD | P15428 | 15-hydroxyprostaglandin dehydrogenase [NAD+] | SwissTargetPrediction/Pubchem |
| 298 | HSD11B1 | P28845 | 11-beta-hydroxysteroid dehydrogenase 1 | SwissTargetPrediction/Pubchem |
| 299 | HSD11B2 | P80365 | 11-beta-hydroxysteroid dehydrogenase 2 | SwissTargetPrediction |
| 300 | HSD17B1 | P14061 | Estradiol 17-beta-dehydrogenase 1 | SwissTargetPrediction/Pubchem |
| 301 | HSD17B2 | P37059 | Estradiol 17-beta-dehydrogenase 2 | SwissTargetPrediction/Pubchem |
| 302 | HSD17B3 | P37058 | Estradiol 17-beta-dehydrogenase 3 | SwissTargetPrediction |
| 303 | HSF1 | Q00613 | Heat shock factor protein 1 | TCMSP/SwissTargetPrediction/Pubchem |
| 304 | HSP90AA1 | P07900 | Heat shock protein HSP 90-alpha | SwissTargetPrediction/Pubchem |
| 305 | HSP90AB1 | P08238 | Heat shock protein HSP 90-beta, HSP 90 | TCMSP/Pubchem |
| 306 | HSPA5 | P11021 | 78 kDa glucose-regulated protein | TCMSP |
| 307 | HSPB1 | P04792 | Heat shock protein beta-1 | TCMSP/Pubchem |
| 308 | HTR2A | P28223 | 5-hydroxytryptamine 2A receptor | TCMSP |
| 309 | HTRA1 | Q05DJ8 | HTRA1 protein | Pubchem |
| 310 | ICAM1 | P05362 | Intercellular adhesion molecule 1 | TCMSP |
| 311 | IDO1 | P14902 | Indoleamine 2,3-dioxygenase | SwissTargetPrediction |
| 312 | IDO2 | Q6ZQW0 | Indoleamine 2,3-dioxygenase 1 | TCMSP |
| 313 | IFNG | P01579 | Interferon gamma | TCMSP/Pubchem |
| 314 | IGF1R | P08069 | Insulin-like growth factor I receptor | SwissTargetPrediction/Pubchem |
| 315 | IGF2 | P01344 | Insulin-like growth factor II | TCMSP |
| 316 | IGFBP3 | P17936 | Insulin-like growth factor-binding protein 3 | TCMSP |
| 317 | IGHG1 | P01857 | Ig gamma-1 chain C region | TCMSP |
| 318 | IL10 | P22301 | Interleukin-10 | TCMSP |
| 319 | IL1A | P01583 | Interleukin-1 alpha | TCMSP |
| 320 | IL1B | P01584 | Interleukin-1 beta | TCMSP |
| 321 | IL2 | P60568 | Interleukin-2 | TCMSP/SwissTargetPrediction |
| 322 | IL4 | P05112 | Interleukin-4 | TCMSP |
| 323 | IL5 | P05113 | Interleukin-5 | TCMSP |
| 324 | IL6 | P05231 | Interleukin-6 | TCMSP |
| 325 | INPPL1 | O15357 | Phosphatidylinositol-3,4,5-trisphosphate 5-phosphatase 2 | TCMSP |
| 326 | INS | P01308 | Insulin | TCMSP |
| 327 | INSR | P06213 | Insulin receptor | TCMSP/SwissTargetPrediction |
| 328 | IRF1 | P10914 | Interferon regulatory factor 1 | TCMSP |
| 329 | ITGA4 | P13612 | Integrin alpha-4 | SwissTargetPrediction |
| 330 | ITGB1 | P05556 | Integrin beta-1 | SwissTargetPrediction |
| 331 | ITGB2 | P05107 | Integrin beta-2 | TCMSP |
| 332 | JUN | P05412 | Transcription factor AP-1 | TCMSP/Pubchem |
| 333 | KCNH2 | Q12809 | Potassium voltage-gated channel subfamily H member 2 | TCMSP/Pubchem |
| 334 | KCNQ1 | P51787 | potassium voltage-gated channel subfamily Q member 1 | Pubchem |
| 335 | KDM3A | Q9Y4C1 | Lysine-specific demethylase 3A | SwissTargetPrediction |
| 336 | KDM4A | O75164 | Lysine-specific demethylase 4A | SwissTargetPrediction |
| 337 | KDM4C | Q9H3R0 | Lysine-specific demethylase 4C | SwissTargetPrediction |
| 338 | KDM4E | B2RXH2 | Lysine-specific demethylase 4D-like | SwissTargetPrediction |
| 339 | KDM6B | O15054 | Lysine-specific demethylase 6B | SwissTargetPrediction |
| 340 | KDR | P35968 | Vascular endothelial growth factor receptor 2 | SwissTargetPrediction/Pubchem |
| 341 | LCK | P06239 | Tyrosine-protein kinase LCK | SwissTargetPrediction/Pubchem |
| 342 | LITAF | Q99732 | Lipopolysaccharide-induced tumor necrosis factor-alpha factor | TCMSP |
| 343 | LSS | P48449 | Lanosterol synthase | STITCH |
| 344 | LTA4H | P09960 | Leukotriene A-4 hydrolase | TCMSP |
| 345 | LTB4R | Q15722 | Leukotriene B4 receptor 1 | SwissTargetPrediction |
| 346 | LYZ | P61626 | Lysozyme | TCMSP |
| 347 | MAOA | P21397 | Amine oxidase [flavin-containing] A | TCMSP/SwissTargetPrediction/Pubchem |
| 348 | MAOB | P27338 | Amine oxidase [flavin-containing] B | TCMSP/SwissTargetPrediction |
| 349 | MAP2 | P11137 | Microtubule-associated protein 2 | TCMSP |
| 350 | MAP3K5 | Q99683 | mitogen-activated protein kinase kinase kinase 5 | Pubchem |
| 351 | MAP4K2 | Q12851 | mitogen-activated protein kinase kinase kinase kinase 2 | Pubchem |
| 352 | MAPK1 | P28482 | Mitogen-activated protein kinase 1 | TCMSP/SwissTargetPrediction |
| 353 | MAPK14 | Q16539 | MAP kinase p38 alpha | SwissTargetPrediction |
| 354 | MAPK3 | P27361 | MAP kinase ERK1 | SwissTargetPrediction |
| 355 | MAPK8 | P45983 | Mitogen-activated protein kinase 8 | TCMSP/SwissTargetPrediction/STITCH |
| 356 | MAPK8IP2 | Q13387 | C-Jun-amino-terminal kinase-interacting protein 2 | TCMSP |
| 357 | MAPT | P10636 | Microtubule-associated protein tau | SwissTargetPrediction/Pubchem |
| 358 | MCL1 | Q07820 | Induced myeloid leukemia cell differentiation protein Mcl-1 | TCMSP/Pubchem/STITCH |
| 359 | MDM2 | Q00987 | p53-binding protein Mdm-2 | SwissTargetPrediction/Pubchem |
| 360 | MDM4 | O15151 | MDM4 regulator of p53 | Pubchem |
| 361 | MET | P08581 | Hepatocyte growth factor receptor | SwissTargetPrediction/Pubchem |
| 362 | MGAM | O43451 | Maltase-glucoamylase, intestinal | TCMSP |
| 363 | MIF | P14174 | Macrophage migration inhibitory factor | SwissTargetPrediction |
| 364 | MME | P08473 | Neprilysin *(by homology)* | SwissTargetPrediction |
| 365 | MMP1 | P03956 | Interstitial collagenase | TCMSP/SwissTargetPrediction/Pubchem |
| 366 | MMP10 | P09238 | Stromelysin-2 | TCMSP |
| 367 | MMP12 | P39900 | Matrix metalloproteinase 12 | SwissTargetPrediction/Pubchem |
| 368 | MMP13 | P45452 | Matrix metalloproteinase 13 | SwissTargetPrediction/Pubchem |
| 369 | MMP2 | P08253 | 72 kDa type IV collagenase | TCMSP/SwissTargetPrediction/Pubchem |
| 370 | MMP3 | P08254 | Stromelysin-1 | TCMSP/SwissTargetPrediction/Pubchem |
| 371 | MMP8 | P22894 | Matrix metalloproteinase 8 | SwissTargetPrediction |
| 372 | MMP9 | P14780 | Matrix metalloproteinase-9 | TCMSP/SwissTargetPrediction/Pubchem |
| 373 | MPG | P29372 | DNA-3-methyladenine glycosylase | SwissTargetPrediction |
| 374 | MPHOSPH8 | Q99549 | M-phase phosphoprotein 8 | Pubchem |
| 375 | MPI | P34949 | mannose phosphate isomerase | Pubchem |
| 376 | MPO | P05164 | Myeloperoxidase | TCMSP/SwissTargetPrediction/Pubchem |
| 377 | MTOR | P42345 | Serine/threonine-protein kinase mTOR | STITCH |
| 378 | MYC | P01106 | Myc proto-oncogene protein | TCMSP |
| 379 | MYLK | Q15746 | Myosin light chain kinase, smooth muscle | SwissTargetPrediction |
| 380 | NAMPT | P43490 | Nicotinamide phosphoribosyltransferase | STITCH |
| 381 | NCF1 | P14598 | Neutrophil cytosol factor 1 | TCMSP |
| 382 | NCOA1 | Q15788 | Nuclear receptor coactivator 1 | TCMSP/Pubchem |
| 383 | NCOA2 | Q15596 | Nuclear receptor coactivator 2 | TCMSP |
| 384 | NCSTN | Q92542 | Nicastrin | SwissTargetPrediction |
| 385 | NEK2 | P51955 | Serine/threonine-protein kinase NEK2 | SwissTargetPrediction/Pubchem |
| 386 | NEK6 | Q9HC98 | Serine/threonine-protein kinase NEK6 | SwissTargetPrediction/Pubchem |
| 387 | NFAT5 | O94916 | Nuclear factor of activated T-cells 5 | STITCH |
| 388 | NFATC3 | Q12968 | Nuclear factor of activated T-cells, cytoplasmic 3 | TCMSP |
| 389 | NFE2L2 | Q16236 | Nuclear factor erythroid 2-related factor 2 | TCMSP/SwissTargetPrediction/Pubchem/STITCH |
| 390 | NFKB1 | P19838 | nuclear factor kappa B subunit 1 | Pubchem |
| 391 | NFKB2 | Q00653 | nuclear factor kappa B subunit 2 | Pubchem |
| 392 | NFKBIA | P25963 | NF-kappa-B inhibitor alpha | TCMSP |
| 393 | NGFR | P08138 | Low affinity neurotrophin receptor p75NTR | SwissTargetPrediction |
| 394 | NKX3-1 | Q99801 | Homeobox protein Nkx-3.1 | TCMSP |
| 395 | NMUR2 | Q9GZQ4 | Neuromedin-U receptor 2 | SwissTargetPrediction/Pubchem |
| 396 | NOS2 | P35228 | Nitric oxide synthase, inducible | TCMSP/SwissTargetPrediction |
| 397 | NOS3 | P29474 | Nitric oxide synthase, endothelial | TCMSP |
| 398 | NOX4 | Q9NPH5 | NADPH oxidase 4 | SwissTargetPrediction/Pubchem |
| 399 | NPC1L1 | Q9UHC9 | Niemann-Pick C1-like protein 1 | SwissTargetPrediction |
| 400 | NPEPPS | P55786 | Puromycin-sensitive aminopeptidase | TCMSP |
| 401 | NQO1 | P15559 | NAD(P)H dehydrogenase [quinone] 1 | TCMSP/STITCH |
| 402 | NQO2 | P16083 | Quinone reductase 2 | SwissTargetPrediction |
| 403 | NR0B2 | Q15466 | Nuclear receptor subfamily 0 group B member 2 | STITCH |
| 404 | NR1H2 | P55055 | LXR-beta | SwissTargetPrediction |
| 405 | NR1H3 | Q13133 | LXR-alpha | SwissTargetPrediction/Pubchem |
| 406 | NR1H4 | Q96RI1 | Bile acid receptor FXR | SwissTargetPrediction |
| 407 | NR1I2 | O75469 | Nuclear receptor subfamily 1 group I member 2 | TCMSP/Pubchem/STITCH |
| 408 | NR1I3 | Q14994 | Nuclear receptor subfamily 1 group I member 3 | TCMSP/SwissTargetPrediction/Pubchem |
| 409 | NR2E3 | Q9Y5X4 | nuclear receptor subfamily 2 group E member 3 | Pubchem |
| 410 | NR3C1 | P04150 | Glucocorticoid receptor | SwissTargetPrediction/Pubchem |
| 411 | NR3C2 | P08235 | Mineralocorticoid receptor | TCMSP/SwissTargetPrediction |
| 412 | NUAK1 | O60285 | NUAK family SNF1-like kinase 1 | SwissTargetPrediction/Pubchem |
| 413 | ODC1 | P11926 | Ornithine decarboxylase | TCMSP |
| 414 | OGFRL1 | Q5TC84 | Opioid growth factor receptor-like protein 1 | SwissTargetPrediction |
| 415 | OPRM1 | P35372 | Mu-type opioid receptor | TCMSP |
| 416 | P4HB | P07237 | Protein disulfide-isomerase | STITCH |
| 417 | PABPC1 | P11940 | poly | Pubchem |
| 418 | PADI1 | Q9ULC6 | Protein-arginine deiminase type-1 | SwissTargetPrediction |
| 419 | PADI2 | Q9Y2J8 | Protein-arginine deiminase type-2 | SwissTargetPrediction |
| 420 | PADI3 | Q9ULW8 | Protein-arginine deiminase type-3 | SwissTargetPrediction |
| 421 | PADI4 | Q9UM07 | Protein-arginine deiminase type-4 | SwissTargetPrediction |
| 422 | PAFAH1B2 | P68402 | platelet activating factor acetylhydrolase 1b catalytic subunit 2 | Pubchem |
| 423 | PAFAH1B3 | Q15102 | platelet activating factor acetylhydrolase 1b catalytic subunit 3 | Pubchem |
| 424 | PARP1 | P09874 | Poly [ADP-ribose] polymerase 1 | TCMSP/SwissTargetPrediction/Pubchem/STITCH |
| 425 | PBRM1 | Q86U86 | polybromo 1 | Pubchem |
| 426 | PCOLCE | Q15113 | Procollagen C-endopeptidase enhancer 1 | TCMSP |
| 427 | PCSK9 | Q8NBP7 | Proprotein convertase subtilisin/kexin type 9 | STITCH |
| 428 | PDE3A | Q14432 | CGMP-inhibited 3',5'-cyclic phosphodiesterase A | TCMSP |
| 429 | PDE4B | Q07343 | Phosphodiesterase 4B | SwissTargetPrediction |
| 430 | PDE4D | Q08499 | Phosphodiesterase 4D | SwissTargetPrediction |
| 431 | PDE5A | O76074 | Phosphodiesterase 5A | SwissTargetPrediction |
| 432 | PECAM1 | P16284 | Platelet endothelial cell adhesion molecule | TCMSP |
| 433 | PFKFB3 | Q16875 | 6-phosphofructo-2-kinase/fructose-2,6-bisphosphatase 3 | SwissTargetPrediction |
| 434 | PGD | P52209 | phosphogluconate dehydrogenase | Pubchem |
| 435 | PGR | P06401 | Progesterone receptor | TCMSP/SwissTargetPrediction/Pubchem |
| 436 | PI4K2A | Q9BTU6 | phosphatidylinositol 4-kinase type 2 alpha | Pubchem |
| 437 | PIK3CA | P42336 | PI3-kinase p110-alpha subunit | SwissTargetPrediction/Pubchem |
| 438 | PIK3CB | P42338 | PI3-kinase p110-beta subunit | SwissTargetPrediction |
| 439 | PIK3CG | P48736 | Phosphatidylinositol-4,5-bisphosphate 3-kinase catalytic subunit, gamma isoform | TCMSP/SwissTargetPrediction/Pubchem |
| 440 | PIK3R1 | P27986 | PI3-kinase p85-alpha subunit | SwissTargetPrediction/Pubchem |
| 441 | PIM1 | P11309 | Serine/threonine-protein kinase PIM1 | SwissTargetPrediction/Pubchem/STITCH |
| 442 | PIM2 | Q9P1W9 | Pim-2 proto-oncogene, serine/threonine kinase | Pubchem |
| 443 | PIP4K2A | P48426 | phosphatidylinositol-5-phosphate 4-kinase type 2 alpha | Pubchem |
| 444 | PKN1 | Q16512 | Protein kinase N1 | SwissTargetPrediction/Pubchem |
| 445 | PLA2G1B | P04054 | Phospholipase A2 group 1B | SwissTargetPrediction/Pubchem |
| 446 | PLAT | P00750 | Tissue-type plasminogen activator | TCMSP |
| 447 | PLAU | P00749 | Urokinase-type plasminogen activator | TCMSP/Pubchem |
| 448 | PLAUR | Q03405 | Urokinase plasminogen activator surface receptor | STITCH |
| 449 | PLG | P00747 | Plasminogen | SwissTargetPrediction |
| 450 | PLK1 | P53350 | Serine/threonine-protein kinase PLK1 | SwissTargetPrediction/Pubchem |
| 451 | POLB | P06746 | DNA polymerase beta | SwissTargetPrediction/Pubchem |
| 452 | POLH | Q9Y253 | DNA polymerase eta | Pubchem |
| 453 | POLI | Q9UNA4 | DNA polymerase iota | Pubchem |
| 454 | POLK | Q9UBT6 | DNA polymerase kappa | Pubchem |
| 455 | PON1 | P27169 | Serum paraoxonase/arylesterase 1 | TCMSP/Pubchem |
| 456 | POR | P16435 | NADPH--cytochrome P450 reductase | TCMSP |
| 457 | PPARA | Q07869 | Peroxisome proliferator-activated receptor alpha | TCMSP/SwissTargetPrediction/Pubchem/STITCH |
| 458 | PPARD | Q03181 | Peroxisome proliferator-activated receptor delta | TCMSP/SwissTargetPrediction |
| 459 | PPARG | P37231 | Peroxisome proliferator activated receptor gamma | TCMSP/SwissTargetPrediction/Pubchem |
| 460 | PPM1B | O75688 | Protein phosphatase 2C beta | SwissTargetPrediction |
| 461 | PPP1CC | P36873 | Serine/threonine protein phosphatase PP1-gamma catalytic subunit | SwissTargetPrediction |
| 462 | PPP2CA | P67775 | Serine/threonine protein phosphatase 2A, catalytic subunit, alpha isoform | SwissTargetPrediction |
| 463 | PPP2R5A | Q15172 | Serine/threonine protein phosphatase 2A, 56 kDa regulatory subunit, alpha isoform | SwissTargetPrediction |
| 464 | PREP | P48147 | Prolyl endopeptidase | SwissTargetPrediction/Pubchem |
| 465 | PRKACA | P17612 | mRNA of PKA Catalytic Subunit C-alpha | TCMSP |
| 466 | PRKCA | P17252 | Protein kinase C alpha type | TCMSP |
| 467 | PRKCB | P05771 | Protein kinase C beta type | TCMSP |
| 468 | PRKCG | P05129 | Protein kinase C gamma type | TCMSP |
| 469 | PRKCH | P24723 | Protein kinase C eta | SwissTargetPrediction |
| 470 | PRNP | F7VJQ1 | Alternative prion protein | STITCH |
| 471 | PRSS1 | P07477 | Trypsin-1 | TCMSP/Pubchem |
| 472 | PRSS2 | P07478 | serine protease 2 | Pubchem |
| 473 | PRSS3 | P35030 | serine protease 3 | Pubchem |
| 474 | PSEN1 | P49768 | Presenilin-1 | SwissTargetPrediction |
| 475 | PSEN2 | P49810 | Presenilin-2 | SwissTargetPrediction |
| 476 | PSENEN | Q9NZ42 | Gamma-secretase subunit PEN-2 | SwissTargetPrediction |
| 477 | PSIP1 | O75475 | PC4 and SFRS1 interacting protein 1 | Pubchem |
| 478 | PSMD3 | O43242 | 26S proteasome non-ATPase regulatory subunit 3 | TCMSP |
| 479 | PTAFR | P25105 | Platelet activating factor receptor | SwissTargetPrediction |
| 480 | PTEN | P60484 | Phosphatidylinositol-3,4,5-trisphosphate 3-phosphatase and dual-specificity protein phosphatase PTEN | TCMSP |
| 481 | PTGDR | Q13258 | Prostanoid DP receptor | SwissTargetPrediction |
| 482 | PTGDR2 | Q9Y5Y4 | G protein-coupled receptor 44 | SwissTargetPrediction |
| 483 | PTGER1 | P34995 | Prostanoid EP1 receptor | SwissTargetPrediction |
| 484 | PTGER2 | P43116 | Prostanoid EP2 receptor (by homology) | SwissTargetPrediction |
| 485 | PTGER3 | P43115 | Prostaglandin E2 receptor EP3 subtype | TCMSP/SwissTargetPrediction |
| 486 | PTGER4 | P35408 | Prostanoid EP4 receptor *(by homology)* | SwissTargetPrediction |
| 487 | PTGES | O14684 | Prostaglandin E synthase | SwissTargetPrediction |
| 488 | PTGFR | P43088 | Prostanoid FP receptor | SwissTargetPrediction |
| 489 | PTGIR | P43119 | Prostanoid IP receptor | SwissTargetPrediction/STITCH |
| 490 | PTGS1 | P23219 | Prostaglandin G/H synthase 1 | TCMSP/SwissTargetPrediction/Pubchem |
| 491 | PTGS2 | P35354 | Prostaglandin G/H synthase 2 | TCMSP/SwissTargetPrediction/STITCH/Pubchem |
| 492 | PTK2 | Q05397 | Focal adhesion kinase 1 | SwissTargetPrediction/Pubchem |
| 493 | PTPN1 | P18031 | Tyrosine-protein phosphatase non-receptor type 1 | TCMSP/SwissTargetPrediction/Pubchem |
| 494 | PTPN11 | Q06124 | Protein-tyrosine phosphatase 2C | SwissTargetPrediction/Pubchem |
| 495 | PTPN2 | P17706 | T-cell protein-tyrosine phosphatase | SwissTargetPrediction/Pubchem |
| 496 | PTPN6 | P29350 | Tyrosine-protein phosphatase non-receptor type 6 | TCMSP/SwissTargetPrediction/Pubchem |
| 497 | PTPRF | P10586 | Receptor-type tyrosine-protein phosphatase F (LAR) | SwissTargetPrediction/Pubchem |
| 498 | PTPRS | Q13332 | Receptor-type tyrosine-protein phosphatase S | SwissTargetPrediction/Pubchem |
| 499 | PYGL | P06737 | Liver glycogen phosphorylase | SwissTargetPrediction/Pubchem |
| 500 | PYGM | P11217 | glycogen phosphorylase, muscle associated | Pubchem |
| 501 | RAC1 | P63000 | Ras-related C3 botulinum toxin substrate 1 | TCMSP |
| 502 | RACGAP1 | Q9H0H5 | Rac GTPase activating protein 1 | Pubchem |
| 503 | RAF1 | P04049 | RAF proto-oncogene serine/threonine-protein kinase | TCMSP |
| 504 | RAPGEF3 | O95398 | Rap guanine nucleotide exchange factor 3 | Pubchem |
| 505 | RARA | P10276 | retinoic acid receptor alpha | Pubchem |
| 506 | RASA1 | P20936 | Ras GTPase-activating protein 1 | TCMSP |
| 507 | RASSF1 | Q9NS23 | Ras association domain-containing protein 1 | TCMSP |
| 508 | RB1 | P06400 | Retinoblastoma-associated protein | TCMSP |
| 509 | RBP4 | P02753 | Plasma retinol-binding protein | SwissTargetPrediction |
| 510 | RELA | Q04206 | Transcription factor p65 | TCMSP/Pubchem |
| 511 | RGS12 | O14924 | regulator of G protein signaling 12 | Pubchem |
| 512 | RGS4 | P49798 | regulator of G protein signaling 4 | Pubchem |
| 513 | RNASEH1 | O60930 | ribonuclease H1 | Pubchem |
| 514 | RORA | P35398 | Nuclear receptor ROR-alpha | SwissTargetPrediction |
| 515 | RORC | P51449 | Nuclear receptor ROR-gamma | SwissTargetPrediction/Pubchem |
| 516 | RPS6KA3 | P51812 | Ribosomal protein S6 kinase alpha 3 | SwissTargetPrediction/STITCH |
| 517 | RUNX1T1 | Q06455 | Protein CBFA2T1 | TCMSP |
| 518 | RUNX2 | Q13950 | Runt-related transcription factor 2 | TCMSP |
| 519 | RXRA | P19793 | Retinoic acid receptor RXR-alpha | TCMSP/Pubchem |
| 520 | S1PR1 | P21453 | Sphingosine 1-phosphate receptor Edg-1 | SwissTargetPrediction |
| 521 | S1PR2 | O95136 | Sphingosine 1-phosphate receptor Edg-5 | SwissTargetPrediction |
| 522 | S1PR3 | Q99500 | Sphingosine 1-phosphate receptor Edg-3 | SwissTargetPrediction |
| 523 | S1PR4 | O95977 | Sphingosine 1-phosphate receptor Edg-6 | SwissTargetPrediction |
| 524 | S1PR5 | Q9H228 | Sphingosine 1-phosphate receptor Edg-8 | SwissTargetPrediction |
| 525 | SAE1 | Q9UBE0 | SUMO-activating enzyme1 | SwissTargetPrediction/Pubchem |
| 526 | SCD | O00767 | Acyl-CoA desaturase | SwissTargetPrediction |
| 527 | SCN10A | Q9Y5Y9 | Sodium channel protein type X alpha subunit *(by homology)* | SwissTargetPrediction |
| 528 | SCN5A | Q14524 | Sodium channel protein type 5 subunit alpha | TCMSP/SwissTargetPrediction |
| 529 | SCN9A | Q15858 | Sodium channel protein type IX alpha subunit | SwissTargetPrediction |
| 530 | SELE | P16581 | E-selectin | TCMSP/SwissTargetPrediction |
| 531 | SELL | P14151 | Leukocyte adhesion molecule-1 | SwissTargetPrediction |
| 532 | SELP | P16109 | P-selectin | TCMSP/SwissTargetPrediction |
| 533 | SERPINA6 | P08185 | Corticosteroid binding globulin | SwissTargetPrediction |
| 534 | SERPINE1 | P05121 | Plasminogen activator inhibitor 1 | TCMSP |
| 535 | SHBG | P04278 | Testis-specific androgen-binding protein | SwissTargetPrediction |
| 536 | SIAE | Q9HAT2 | sialic acid acetylesterase | Pubchem |
| 537 | SIGMAR1 | Q99720 | Sigma opioid receptor | SwissTargetPrediction |
| 538 | SIRT1 | Q96EB6 | NAD-dependent deacetylase sirtuin 1 | SwissTargetPrediction |
| 539 | SLC22A12 | Q96S37 | Solute carrier family 22 member 12 | SwissTargetPrediction/Pubchem |
| 540 | SLC22A6 | Q4U2R8 | Solute carrier family 22 member 6 *(by homology)* | SwissTargetPrediction/STITCH |
| 541 | SLC22A7 | Q9Y694 | Solute carrier family 22 member 7 | STITCH |
| 542 | SLC22A8 | Q8TCC7 | Solute carrier family 22 member 8 | STITCH |
| 543 | SLC2A2 | P11168 | Solute carrier family 2, facilitated glucose transporter member 2 | STITCH |
| 544 | SLC2A4 | P14672 | Solute carrier family 2, facilitated glucose transporter member 4 | TCMSP |
| 545 | SLC6A2 | P23975 | Sodium-dependent noradrenaline transporter | TCMSP/SwissTargetPrediction |
| 546 | SLC6A3 | Q01959 | Sodium-dependent dopamine transporter | TCMSP/SwissTargetPrediction |
| 547 | SLC6A4 | P31645 | Sodium-dependent serotonin transporter | TCMSP/SwissTargetPrediction |
| 548 | SLCO1B1 | Q9Y6L6 | solute carrier organic anion transporter family member 1B1 | Pubchem |
| 549 | SLCO1B3 | Q9NPD5 | solute carrier organic anion transporter family member 1B3 | Pubchem |
| 550 | SLK | Q9H2G2 | STE20 like kinase | Pubchem |
| 551 | SMAD2 | Q15796 | SMAD family member 2 | Pubchem |
| 552 | SMAD3 | P84022 | SMAD family member 3 | Pubchem |
| 553 | SMN2 | Q16637 | survival of motor neuron 2, centromeric | Pubchem |
| 554 | SNCA | P37840 | Alpha-synuclein | TCMSP |
| 555 | SOD1 | P00441 | Superoxide dismutase [Cu-Zn] | TCMSP |
| 556 | SORD | Q00796 | sorbitol dehydrogenase | Pubchem |
| 557 | SPP1 | P10451 | Osteopontin | TCMSP |
| 558 | SQLE | Q14534 | Squalene monooxygenase | SwissTargetPrediction |
| 559 | SRC | P12931 | Tyrosine-protein kinase SRC | SwissTargetPrediction/Pubchem |
| 560 | SREBF1 | P36956 | Sterol regulatory element-binding protein 1 | STITCH |
| 561 | SREBF2 | Q12772 | Sterol regulatory element-binding protein 2 | SwissTargetPrediction |
| 562 | ST6GAL1 | P15907 | Beta-galactoside alpha-2,6-sialyltransferase 1 | SwissTargetPrediction |
| 563 | STAT1 | P42224 | Signal transducer and activator of transcription 1-alpha/beta | TCMSP |
| 564 | STAT3 | P40763 | Signal transducer and activator of transcription 3 | TCMSP/SwissTargetPrediction |
| 565 | STK16 | O75716 | serine/threonine kinase 16 | Pubchem |
| 566 | STK17B | O94768 | Serine/threonine-protein kinase 17B | STITCH |
| 567 | STK33 | Q9BYT3 | serine/threonine kinase 33 | Pubchem |
| 568 | SULT1E1 | P49888 | Estrogen sulfotransferase | TCMSP |
| 569 | SYK | P43405 | Tyrosine-protein kinase SYK | SwissTargetPrediction/Pubchem |
| 570 | TBXA2R | P21731 | Thromboxane A2 receptor | TCMSP |
| 571 | TBXAS1 | P24557 | Thromboxane-A synthase | SwissTargetPrediction |
| 572 | TDP1 | Q9NUW8 | tyrosyl-DNA phosphodiesterase 1 | Pubchem |
| 573 | TERT | O14746 | Telomerase reverse transcriptase | SwissTargetPrediction |
| 574 | TGFB1 | P01137 | Transforming growth factor beta-1 | TCMSP |
| 575 | THBD | P07204 | Thrombomodulin | TCMSP |
| 576 | THRA | P10827 | Thyroid hormone receptor alpha | SwissTargetPrediction |
| 577 | THRB | P10828 | Thyroid hormone receptor beta-1 | SwissTargetPrediction/Pubchem |
| 578 | TLR4 | O00206 | Toll-like receptor 4 *(by homology)* | SwissTargetPrediction |
| 579 | TLR9 | Q9NR96 | Toll-like receptor (TLR7/TLR9) | SwissTargetPrediction |
| 580 | TMPRSS11D | O60235 | Transmembrane protease serine 11D | STITCH |
| 581 | TNF | P01375 | Tumor necrosis factor | TCMSP/SwissTargetPrediction |
| 582 | TNFRSF10B | O14763 | TNF receptor superfamily member 10b | Pubchem |
| 583 | TNKS | O95271 | Tankyrase-1 | SwissTargetPrediction/Pubchem |
| 584 | TNKS2 | Q9H2K2 | Tankyrase-2 | SwissTargetPrediction/Pubchem |
| 585 | TOP1 | P11387 | DNA topoisomerase 1 | TCMSP/SwissTargetPrediction/Pubchem/STITCH |
| 586 | TOP2A | P11388 | DNA topoisomerase 2-alpha | TCMSP/SwissTargetPrediction/Pubchem/STITCH |
| 587 | TP53 | P04637 | Cellular tumor antigen p53 | TCMSP/Pubchem |
| 588 | TPMT | P51580 | Thiopurine S-methyltransferase | SwissTargetPrediction |
| 589 | TRA | P0DSE1 | T cell receptor alpha locus | Pubchem |
| 590 | TTR | P02766 | Transthyretin | SwissTargetPrediction/Pubchem |
| 591 | TYR | P14679 | Tyrosinase | SwissTargetPrediction/Pubchem/STITCH |
| 592 | UBA2 | Q9UBT2 | SUMO-activating enzyme2 | SwissTargetPrediction/Pubchem |
| 593 | UBE2I | P63279 | ubiquitin conjugating enzyme E2 I | Pubchem |
| 594 | UGT1A3 | P35503 | UDP-glucuronosyltransferase 1-3 | STITCH |
| 595 | UGT1A7 | Q9HAW7 | UDP-glucuronosyltransferase 1-7 | STITCH |
| 596 | UGT1A8 | Q9HAW9 | UDP-glucuronosyltransferase 1-8 | STITCH |
| 597 | UGT1A9 | O60656 | UDP-glucuronosyltransferase 1-9 | STITCH |
| 598 | UGT2B10 | P36537 | UDP-glucuronosyltransferase 2B10 | STITCH |
| 599 | UGT2B11 | O75310 | UDP-glucuronosyltransferase 2B11 | STITCH |
| 600 | UGT2B7 | P16662 | UDP-glucuronosyltransferase 2B7 | SwissTargetPrediction |
| 601 | UGT3A1 | Q6NUS8 | UDP-glucuronosyltransferase 3A1 | STITCH |
| 602 | USP1 | O94782 | ubiquitin specific peptidase 1 | Pubchem |
| 603 | VCAM1 | P19320 | Vascular cell adhesion protein 1 | TCMSP |
| 604 | VCP | P55072 | Transitional endoplasmic reticulum ATPase | SwissTargetPrediction/Pubchem |
| 605 | VDR | P11473 | Vitamin D receptor | SwissTargetPrediction |
| 606 | VEGFA | P15692 | Vascular endothelial growth factor A | TCMSP/STITCH |
| 607 | WRN | Q14191 | Werner syndrome RecQ like helicase | Pubchem |
| 608 | XDH | P47989 | Xanthine dehydrogenase/oxidase | TCMSP/SwissTargetPrediction/Pubchem |
| 609 | YES1 | P07947 | YES proto-oncogene 1, Src family tyrosine kinase | Pubchem |
| 610 | YWHAB | P31946 | tyrosine 3-monooxygenase/tryptophan 5-monooxygenase activation protein beta | Pubchem |
