# Supplementary material for: Mechanisms of Spica Prunellae against thyroid-associated Ophthalmopathy based on network pharmacology and molecular docking
Source: BMC Complement Med Ther. 2020 Jul 20;20:229. doi: 10.1186/s12906-020-03022-2 (PMC7372882; doi:10.1186/s12906-020-03022-2)
Supplement: Supplementary file 2 — Additional file 2: Table S2 TAO related targets [file 12906_2020_3022_MOESM2_ESM.docx]

**Table S2** TAO related targets

| **Number** | **Gene name** | **Uniprot ID** | **Protein name** | **Database** |
| --- | --- | --- | --- | --- |
| 1 | A2M | P01023 | Alpha-2-macroglobulin | CTD |
| 2 | ABCB1 | P08183 | ATP-dependent translocase ABCB1 | CTD/Drugbank |
| 3 | ABCB7 | O75027 | ATP Binding Cassette Subfamily B Member 7 | GeneCard |
| 4 | ABCF1 | Q8NE71 | ATP Binding Cassette Subfamily F Member 1 | GeneCard |
| 5 | ABCG2 | Q9UNQ0 | ATP-binding cassette sub-family G member 2 | CTD |
| 6 | ABO | P16442 | Histo-blood group ABO system transferase | CTD |
| 7 | ACAD10 | Q6JQN1 | Acyl-CoA dehydrogenase family member 10 | CTD |
| 8 | ACAD8 | Q9UKU7 | Isobutyryl-CoA dehydrogenase, mitochondrial | CTD |
| 9 | ACKR3 | P25106 | Atypical chemokine receptor 3 | CTD |
| 10 | ACOT12 | Q8WYK0 | Acetyl-coenzyme A thioesterase | CTD |
| 11 | ACP1 | P24666 | Acid Phosphatase 1 | GeneCard |
| 12 | ACSM2A | Q08AH3 | Acyl-coenzyme A synthetase ACSM2A, mitochondrial | CTD |
| 13 | ACSM5 | Q6NUN0 | Acyl-coenzyme A synthetase ACSM5, mitochondrial | CTD |
| 14 | ADIPOQ | Q15848 | Adiponectin | CTD/GeneCard |
| 15 | ADORA2A | P29274 | Adenosine receptor A2a | CTD |
| 16 | ADRB2 | P07550 | Adrenoceptor Beta 2 | GeneCard |
| 17 | ADSSL1 | Q8N142 | Adenylosuccinate synthetase isozyme 1 | CTD |
| 18 | AFM | P43652 | Afamin | CTD |
| 19 | AGMO | Q6ZNB7 | Alkylglycerol monooxygenase | CTD |
| 20 | AGRP | O00253 | Agouti-related protein | CTD |
| 21 | AGTR1 | P30556 | Type-1 angiotensin II receptor | CTD |
| 22 | AGXT2 | Q9BYV1 | Alanine--glyoxylate aminotransferase 2, mitochondrial | CTD |
| 23 | AIRE | O43918 | Autoimmune Regulator | GeneCard/OMIM |
| 24 | AJAP1 | Q9UKB5 | Adherens junction-associated protein 1 | CTD |
| 25 | AKT1 | P31749 | RAC-alpha serine/threonine-protein kinase | CTD |
| 26 | AKT2 | P31751 | AKT Serine/Threonine Kinase 2 | GeneCard |
| 27 | ALB | P02768 | Serum albumin | Drugbank/GeneCard |
| 28 | ALPL | P05186 | Alkaline phosphatase, tissue-nonspecific isozyme | CTD |
| 29 | ALPP | P05187 | Alkaline Phosphatase, Placental | GeneCard |
| 30 | AMHR2 | Q16671 | Anti-Muellerian hormone type-2 receptor | CTD |
| 31 | AMIGO2 | Q86SJ2 | Amphoterin-induced protein 2 | CTD |
| 32 | ANAPC10 | Q9UM13 | Anaphase-promoting complex subunit 10 | CTD |
| 33 | ANKRD1 | Q15327 | Ankyrin repeat domain-containing protein 1 | CTD |
| 34 | ANKRD2 | Q9GZV1 | Ankyrin repeat domain-containing protein 2 | CTD |
| 35 | ANTXR1 | Q9H6X2 | Anthrax toxin receptor 1 | CTD |
| 36 | ANXA10 | Q9UJ72 | Annexin A10 | CTD |
| 37 | AOX1 | Q06278 | Aldehyde oxidase | CTD |
| 38 | APC | P25054 | APC Regulator Of WNT Signaling Pathway | GeneCard |
| 39 | APC2 | O95996 | Adenomatous polyposis coli protein 2 | CTD |
| 40 | APOLD1 | Q96LR9 | Apolipoprotein L Domain Containing 1 | GeneCard |
| 41 | AR | P10275 | Androgen receptor | CTD |
| 42 | ARFGEF2 | Q9Y6D5 | Brefeldin A-inhibited guanine nucleotide-exchange protein 2 | CTD |
| 43 | ARRB1 | P49407 | Arrestin Beta 1 | GeneCard |
| 44 | ART4 | Q93070 | Ecto-ADP-ribosyltransferase 4 | CTD |
| 45 | ASIC3 | Q9UHC3 | Acid-sensing ion channel 3 | CTD |
| 46 | ASNS | P08243 | Asparagine synthetase [glutamine-hydrolyzing] | CTD |
| 47 | ASPDH | A6ND91 | Putative L-aspartate dehydrogenase | CTD |
| 48 | ATP5F1A | P25705 | ATP synthase subunit alpha, mitochondrial | CTD |
| 49 | ATP5F1C | P36542 | ATP synthase subunit gamma, mitochondrial | CTD |
| 50 | ATP5F1D | P30049 | ATP synthase subunit delta, mitochondrial | CTD |
| 51 | ATP5F1E | P56381 | ATP synthase subunit epsilon, mitochondrial, ATPase subunit epsilon | CTD |
| 52 | ATP5ME | P56385 | ATP synthase subunit e, mitochondrial, ATPase subunit e | CTD |
| 53 | ATP5MF | P56134 | ATP synthase subunit f, mitochondrial | CTD |
| 54 | ATP5MG | O75964 | ATP synthase subunit g, mitochondrial, ATPase subunit g | CTD |
| 55 | ATP5PB | P24539 | ATP synthase F | CTD |
| 56 | ATP5PF | P18859 | ATP synthase-coupling factor 6, mitochondrial | CTD |
| 57 | ATP5PO | P48047 | ATP synthase subunit O, mitochondrial | CTD/GeneCard |
| 58 | ATP6V1E1 | P36543 | V-type proton ATPase subunit E 1 | CTD |
| 59 | ATRX | P46100 | Transcriptional regulator ATRX | OMIM |
| 60 | AVPR1A | P37288 | Vasopressin V1a receptor | OMIM |
| 61 | AZGP1 | P25311 | Alpha-2-Glycoprotein 1, Zinc-Binding | GeneCard |
| 62 | AZU1 | P20160 | Azurocidin 1 | GeneCard |
| 63 | B3GNT4 | Q9C0J1 | N-acetyllactosaminide beta-1,3-N-acetylglucosaminyltransferase 4 | CTD |
| 64 | BAK1 | Q16611 | BCL2 Antagonist/Killer 1 | GeneCard |
| 65 | BANK1 | Q8NDB2 | B Cell Scaffold Protein With Ankyrin Repeats 1 | GeneCard |
| 66 | BAX | Q07812 | Apoptosis regulator BAX | CTD/GeneCard |
| 67 | BCL2 | P10415 | Apoptosis regulator Bcl-2 | CTD/GeneCard |
| 68 | BCL2A1 | Q16548 | Bcl-2-related protein A1 | CTD |
| 69 | BCL2L1 | Q07817 | BCL2 Like 1 | GeneCard |
| 70 | BCS1L | Q9Y276 | Mitochondrial chaperone BCS1 | CTD |
| 71 | BGLAP | P02818 | Osteocalcin | CTD |
| 72 | BMP4 | P12644 | Bone Morphogenetic Protein 4 | GeneCard |
| 73 | BMP6 | P22004 | Bone Morphogenetic Protein 6 | GeneCard |
| 74 | BPIFA1 | Q9NP55 | BPI fold-containing family A member 1 | OMIM |
| 75 | BRD4 | O60885 | Bromodomain Containing 4 | GeneCard |
| 76 | BRSK1 | Q8TDC3 | Serine/threonine-protein kinase BRSK1 | CTD |
| 77 | BST2 | Q10589 | Bone marrow stromal antigen 2 | CTD |
| 78 | BTBD16 | Q32M84 | BTB/POZ domain-containing protein 16 | CTD |
| 79 | BTG2 | P78543 | BTG Anti-Proliferation Factor 2 | GeneCard |
| 80 | C11ORF24 | Q96F05 | Uncharacterized protein C11orf24 | CTD |
| 81 | C16ORF46 | Q6P387 | Uncharacterized protein C16orf46 | CTD |
| 82 | C1QTNF5 | Q9BXJ0 | Complement C1q tumor necrosis factor-related protein 5 | OMIM |
| 83 | C4ORF3 | Q8WVX3 | Uncharacterized protein C4orf3 | CTD |
| 84 | C5AR2 | Q9P296 | C5a anaphylatoxin chemotactic receptor 2 | CTD |
| 85 | CA5A | P35218 | Carbonic anhydrase 5A, mitochondrial | CTD |
| 86 | CACNA1A | O00555 | Transcription factor SOX-9 | OMIM |
| 87 | CACNA1S | Q13698 | Calcium Voltage-Gated Channel Subunit Alpha1 S | GeneCard/OMIM |
| 88 | CAD | P27708 | Zinc finger Y-chromosomal protein | OMIM |
| 89 | CALCA | P06881 | Calcitonin gene-related peptide 1 | CTD |
| 90 | CALCB | P10092 | Calcitonin gene-related peptide 2 | CTD |
| 91 | CALHM6 | Q5R3K3 | Calcium homeostasis modulator protein 6 | CTD |
| 92 | CAPN2 | P17655 | Calpain-2 catalytic subunit | CTD |
| 93 | CARD14 | Q9BXL6 | Caspase recruitment domain-containing protein 14 | CTD |
| 94 | CASP3 | P42574 | Caspase-3 | CTD |
| 95 | CASP8 | Q14790 | Caspase 8 | GeneCard |
| 96 | CASQ1 | P31415 | Calsequestrin 1 | GeneCard |
| 97 | CASQ2 | O14958 | Calsequestrin 2 | GeneCard |
| 98 | CAT | P04040 | Catalase | CTD/GeneCard |
| 99 | CATSPERD | Q86XM0 | Cation channel sperm-associated protein subunit delta | CTD |
| 100 | CCDC153 | Q494R4 | Coiled-coil domain-containing protein 153 | CTD |
| 101 | CCL11 | P51671 | Eotaxin | CTD |
| 102 | CCL13 | Q99616 | C-C motif chemokine 13 | CTD |
| 103 | CCL2 | P13500 | C-C Motif Chemokine Ligand 2 | GeneCard |
| 104 | CCL20 | P78556 | C-C motif chemokine 20 | CTD |
| 105 | CCL5 | P13501 | C-C Motif Chemokine Ligand 5 | GeneCard |
| 106 | CCN1 | O00622 | Cellular Communication Network Factor 1 | GeneCard |
| 107 | CCN2 | P29279 | CCN family member 2 | CTD/GeneCard |
| 108 | CCND1 | P24385 | Cyclin D1 | GeneCard |
| 109 | CCNT1 | O60563 | Cyclin-T1 | CTD |
| 110 | CCR1 | P32246 | C-C Motif Chemokine Receptor 1 | GeneCard |
| 111 | CD1A | P06126 | T-cell surface glycoprotein CD1a | CTD |
| 112 | CD1D | P15813 | Antigen-presenting glycoprotein CD1d | CTD |
| 113 | CD28 | P10747 | CD28 Molecule | GeneCard |
| 114 | CD2AP | Q9Y5K6 | CD2-associated protein | CTD |
| 115 | CD300A | Q9UGN4 | CMRF35-like molecule 8 | CTD |
| 116 | CD300LB | A8K4G0 | CMRF35-like molecule 7 | CTD |
| 117 | CD300LF | Q8TDQ1 | CMRF35-like molecule 1 | CTD |
| 118 | CD40 | P25942 | CD40 Molecule | GeneCard |
| 119 | CD40LG | P29965 | CD40 Ligand | GeneCard |
| 120 | CD44 | P16070 | CD44 Molecule (Indian Blood Group) | GeneCard |
| 121 | CD5 | P06127 | CD5 Molecule | GeneCard |
| 122 | CD52 | P31358 | CD52 Molecule | GeneCard |
| 123 | CD58 | P19256 | CD58 Molecule | GeneCard |
| 124 | CD69 | Q07108 | CD69 Molecule | GeneCard |
| 125 | CD79A | P11912 | CD79a Molecule | GeneCard |
| 126 | CD80 | P33681 | CD80 Molecule | GeneCard |
| 127 | CD86 | P42081 | CD86 Molecule | GeneCard |
| 128 | CDC20B | Q86Y33 | Cell division cycle protein 20 homolog B | CTD |
| 129 | CDHR1 | Q96JP9 | Cadherin-related family member 1 | CTD |
| 130 | CDKN1A | P38936 | Cyclin-dependent kinase inhibitor 1 | CTD |
| 131 | CDKN1B | P46527 | Cyclin-dependent kinase inhibitor 1B | CTD |
| 132 | CDKN2B | P42772 | Cyclin-dependent kinase 4 inhibitor B | CTD |
| 133 | CDR2L | Q86X02 | Cerebellar degeneration-related protein 2-like | CTD |
| 134 | CHL1 | O00533 | Neural cell adhesion molecule L1-like protein | CTD |
| 135 | CHRNA6 | Q15825 | Neuronal acetylcholine receptor subunit alpha-6 | CTD |
| 136 | CHRNG | P07510 | Acetylcholine receptor subunit gamma | CTD |
| 137 | CLCN7 | P51798 | H(+)/Cl(-) exchange transporter 7 | OMIM |
| 138 | CLDN23 | Q96B33 | Claudin-23 | CTD |
| 139 | CLEC14A | Q86T13 | C-type lectin domain family 14 member A | CTD |
| 140 | CLEC3B | P05452 | Tetranectin | CTD |
| 141 | CLGN | O14967 | Calmegin | CTD |
| 142 | CLN3 | Q13286 | CLN3 Lysosomal/Endosomal Transmembrane Protein, Battenin | GeneCard |
| 143 | CLRN3 | Q8NCR9 | Clarin-3 | CTD |
| 144 | CLTC | Q00610 | Clathrin Heavy Chain | GeneCard |
| 145 | COG8 | Q96MW5 | Conserved oligomeric Golgi complex subunit 8 | CTD |
| 146 | COL11A1 | P12107 | Collagen alpha-1 | OMIM |
| 147 | COL11A2 | P13942 | Collagen Type XI Alpha 2 Chain | GeneCard |
| 148 | COL13A1 | Q5TAT6 | Collagen Type XIII Alpha 1 Chain | GeneCard |
| 149 | COL1A1 | P02452 | Collagen alpha-1 | CTD/GeneCard |
| 150 | COL1A2 | P08123 | Collagen Type I Alpha 2 Chain | GeneCard |
| 151 | COL4A1 | P02462 | Collagen alpha-1(IV) chain | CTD |
| 152 | COL4A5 | P29400 | Collagen Type IV Alpha 5 Chain | GeneCard |
| 153 | COL5A2 | P05997 | Collagen Type V Alpha 2 Chain | GeneCard |
| 154 | COL9A1 | P20849 | Collagen Type IX Alpha 1 Chain | GeneCard |
| 155 | COL9A3 | Q14050 | Collagen Type IX Alpha 3 Chain | GeneCard |
| 156 | COMMD5 | Q9GZQ3 | COMM domain-containing protein 5 | CTD |
| 157 | COQ9 | O75208 | Ubiquinone biosynthesis protein COQ9, mitochondrial | CTD |
| 158 | COX7A2L | O14548 | Cytochrome c oxidase subunit 7A-related protein, mitochondrial | CTD |
| 159 | CP | P00450 | Ceruloplasmin | GeneCard |
| 160 | CRABP1 | P29762 | Cellular Retinoic Acid Binding Protein 1 | GeneCard |
| 161 | CSF3 | P09919 | Colony Stimulating Factor 3 | GeneCard |
| 162 | CSN2 | P05814 | Beta-casein | CTD |
| 163 | CSNK1D | P48730 | Casein kinase I isoform delta | OMIM |
| 164 | CSRP3 | P50461 | Cysteine and glycine-rich protein 3 | CTD |
| 165 | CTLA4 | P16410 | Cytotoxic T-Lymphocyte Associated Protein 4 | GeneCard/OMIM |
| 166 | CTRL | P40313 | Chymotrypsin-like protease CTRL-1 | CTD |
| 167 | CTTNBP2 | Q8WZ74 | Cortactin-binding protein 2 | CTD |
| 168 | CXCL1 | P09341 | Growth-regulated alpha protein | CTD |
| 169 | CXCL10 | P02778 | C-X-C Motif Chemokine Ligand 10 | GeneCard |
| 170 | CXCL11 | O14625 | C-X-C Motif Chemokine Ligand 11 | GeneCard |
| 171 | CXCL12 | P48061 | C-X-C Motif Chemokine Ligand 12 | GeneCard |
| 172 | CXCL2 | P19875 | C-X-C motif chemokine 2 | CTD |
| 173 | CXCL3 | P19876 | C-X-C motif chemokine 3 | CTD |
| 174 | CXCL8 | P10145 | Interleukin-8 | CTD/GeneCard |
| 175 | CXCL9 | Q07325 | C-X-C Motif Chemokine Ligand 9 | GeneCard |
| 176 | CXCR3 | P49682 | C-X-C Motif Chemokine Receptor 3 | GeneCard |
| 177 | CXCR4 | P61073 | C-X-C chemokine receptor type 4 | CTD/GeneCard |
| 178 | CYP19A1 | P11511 | Aromatase | OMIM |
| 179 | CYP1A1 | P04798 | Cytochrome P450 Family 1 Subfamily A Member 1 | GeneCard |
| 180 | CYP1B1 | Q16678 | Cytochrome P450 1B1 | Drugbank |
| 181 | CYP27B1 | O15528 | Cytochrome P450 Family 27 Subfamily B Member 1 | GeneCard |
| 182 | CYP2A6 | P11509 | Cytochrome P450 2A6 | Drugbank |
| 183 | CYP2A7 | P20853 | Cytochrome P450 2A7 | CTD |
| 184 | CYP2B6 | P20813 | Cytochrome P450 2B6 | Drugbank |
| 185 | CYP2C19 | P33261 | Cytochrome P450 2C19 | Drugbank |
| 186 | CYP2C8 | P10632 | Cytochrome P450 2C8 | Drugbank |
| 187 | CYP2C9 | P11712 | Cytochrome P450 2C9 | Drugbank |
| 188 | CYP2D6 | P10635 | Cytochrome P450 Family 2 Subfamily D Member 6 | GeneCard |
| 189 | CYP2E1 | P05181 | Cytochrome P450 Family 2 Subfamily E Member 1 | GeneCard |
| 190 | CYP3A4 | P08684 | Cytochrome P450 3A4 | CTD/Drugbank/Drugbank |
| 191 | CYP3A43 | Q9HB55 | Cytochrome P450 3A43 | Drugbank |
| 192 | CYP3A5 | P20815 | Cytochrome P450 3A5 | Drugbank/Drugbank |
| 193 | CYP3A7 | P24462 | Cytochrome P450 3A7 | Drugbank |
| 194 | DBP | Q10586 | D-Box Binding PAR BZIP Transcription Factor | GeneCard |
| 195 | DCSTAMP | Q9H295 | Dendritic cell-specific transmembrane protein | CTD |
| 196 | DDIT4 | Q9NX09 | DNA damage-inducible transcript 4 protein | CTD |
| 197 | DDTL | A6NHG4 | D-dopachrome decarboxylase-like protein | CTD |
| 198 | DDX41 | Q9UJV9 | Probable ATP-dependent RNA helicase DDX41 | CTD |
| 199 | DDX58 | O95786 | Probable ATP-dependent RNA helicase DDX58 | CTD |
| 200 | DEFB4A | O15263 | Defensin Beta 4A | GeneCard |
| 201 | DHRSX | Q8N5I4 | Dehydrogenase/reductase SDR family member on chromosome X | OMIM |
| 202 | DICER1 | Q9UPY3 | Endoribonuclease Dicer | OMIM |
| 203 | DIO2 | Q92813 | Type II iodothyronine deiodinase | CTD/GeneCard/OMIM |
| 204 | DLGAP2 | Q9P1A6 | Disks large-associated protein 2 | CTD |
| 205 | DLK1 | P80370 | Delta Like Non-Canonical Notch Ligand 1 | GeneCard |
| 206 | DMC1 | Q14565 | Meiotic recombination protein DMC1/LIM15 homolog | CTD |
| 207 | DMD | P11532 | Dystrophin | CTD |
| 208 | DMP1 | Q13316 | Dentin matrix acidic phosphoprotein 1 | CTD |
| 209 | DMPK | Q09013 | Myotonin-protein kinase | CTD |
| 210 | DMRT1 | Q9Y5R6 | Doublesex- and mab-3-related transcription factor 1 | OMIM |
| 211 | DMWD | Q09019 | Dystrophia myotonica WD repeat-containing protein | CTD |
| 212 | DNAAF2 | Q9NVR5 | Protein kintoun | CTD |
| 213 | DNAJA3 | Q96EY1 | DnaJ homolog subfamily A member 3, mitochondrial | CTD |
| 214 | DNAJB7 | Q7Z6W7 | DnaJ homolog subfamily B member 7 | CTD |
| 215 | DNAJC5B | Q9UF47 | DnaJ homolog subfamily C member 5B | CTD |
| 216 | DNAJC5G | Q8N7S2 | DnaJ homolog subfamily C member 5G | CTD |
| 217 | DTX3L | Q8TDB6 | E3 ubiquitin-protein ligase DTX3L | OMIM |
| 218 | E2F4 | Q16254 | Transcription factor E2F4 | CTD |
| 219 | ECI2 | O75521 | Enoyl-CoA delta isomerase 2, mitochondrial, EC 5.3.3.8 | CTD |
| 220 | EDDM3B | P56851 | Epididymal secretory protein E3-beta | CTD |
| 221 | EDN1 | P05305 | Endothelin-1 | CTD |
| 222 | EDN2 | P20800 | Endothelin-2 | CTD |
| 223 | EDNRA | P25101 | Endothelin Receptor Type A | GeneCard |
| 224 | EDNRB | P24530 | Endothelin receptor type B | CTD |
| 225 | EFCAB1 | Q9HAE3 | EF-hand calcium-binding domain-containing protein 1 | CTD |
| 226 | EFEMP1 | Q12805 | EGF-containing fibulin-like extracellular matrix protein 1 | CTD |
| 227 | EGF | P01133 | Epidermal Growth Factor | GeneCard |
| 228 | EGLN2 | Q96KS0 | Egl nine homolog 2 | CTD |
| 229 | ELF1 | P32519 | ETS-related transcription factor Elf-1 | OMIM |
| 230 | ELMOD3 | Q96FG2 | ELMO domain-containing protein 3 | CTD |
| 231 | ELOVL4 | Q9GZR5 | Elongation of very long chain fatty acids protein 4 | CTD |
| 232 | ENTPD7 | Q9NQZ7 | Ectonucleoside triphosphate diphosphohydrolase 7 | CTD |
| 233 | ENTPD8 | Q5MY95 | Ectonucleoside triphosphate diphosphohydrolase 8 | CTD |
| 234 | EPN3 | Q9H201 | Epsin-3 | CTD |
| 235 | ERBB2 | P04626 | Erb-B2 Receptor Tyrosine Kinase 2 | GeneCard |
| 236 | ERICH6 | Q7L0X2 | Glutamate-rich protein 6 | CTD |
| 237 | ESR2 | Q92731 | Estrogen Receptor 2 | GeneCard |
| 238 | ETNK2 | Q9NVF9 | Ethanolamine kinase 2 | CTD |
| 239 | ETS1 | P14921 | Protein C-ets-1 | OMIM |
| 240 | EZH2 | Q15910 | Histone-lysine N-methyltransferase EZH2 | OMIM |
| 241 | F8 | P00451 | Coagulation factor VIII | CTD |
| 242 | FABP4 | P15090 | Fatty acid-binding protein, adipocyte | CTD |
| 243 | FAM166A | Q6J272 | Protein FAM166A | CTD |
| 244 | FAM167A | Q96KS9 | Family With Sequence Similarity 167 Member A | GeneCard |
| 245 | FAM210A | Q96ND0 | Protein FAM210A | CTD |
| 246 | FAM241A | Q8N8J7 | Uncharacterized protein FAM241A | CTD |
| 247 | FAR2 | Q96K12 | Fatty acyl-CoA reductase 2 | CTD |
| 248 | FAS | P25445 | Fas Cell Surface Death Receptor | GeneCard |
| 249 | FASLG | P48023 | Fas Ligand | GeneCard |
| 250 | FBLN1 | P23142 | Fibulin-1 | CTD |
| 251 | FBN1 | P35555 | Fibrillin 1 | GeneCard |
| 252 | FBXO15 | Q8NCQ5 | F-box only protein 15 | CTD |
| 253 | FBXO22 | Q8NEZ5 | F-box only protein 22 | CTD |
| 254 | FBXO32 | Q969P5 | F-box only protein 32 | CTD |
| 255 | FCER2 | P06734 | Low affinity immunoglobulin epsilon Fc receptor | CTD |
| 256 | FCGR2B | P31994 | Low affinity immunoglobulin gamma Fc region receptor II-b | OMIM |
| 257 | FCGR2C | P31995 | Low affinity immunoglobulin gamma Fc region receptor II-c | OMIM |
| 258 | FCGR3B | O75015 | Low affinity immunoglobulin gamma Fc region receptor III-B | OMIM |
| 259 | FCRL3 | Q96P31 | Fc Receptor Like 3 | GeneCard |
| 260 | FGF8 | P55075 | Fibroblast growth factor 8 | OMIM |
| 261 | FGFR1OP | O95684 | FGFR1 Oncogene Partner | GeneCard |
| 262 | FLNB | O75369 | Filamin B | GeneCard/OMIM |
| 263 | FN1 | P02751 | Fibronectin 1 | GeneCard |
| 264 | FOXE1 | O00358 | Forkhead Box E1 | GeneCard |
| 265 | FOXO1 | Q12778 | Forkhead box protein O1 | CTD |
| 266 | FOXP3 | Q9BZS1 | Forkhead Box P3 | GeneCard |
| 267 | FTMT | Q8N4E7 | Ferritin, mitochondrial | CTD |
| 268 | FUOM | A2VDF0 | Fucose mutarotase | CTD |
| 269 | FUT1 | P19526 | Galactoside 2-alpha-L-fucosyltransferase 1 | CTD |
| 270 | FUT7 | Q11130 | Alpha-(1,3)-fucosyltransferase 7 | CTD |
| 271 | GABPA | Q06546 | GA-binding protein alpha chain | OMIM |
| 272 | GABRG3 | Q99928 | Gamma-aminobutyric acid receptor subunit gamma-3 | CTD |
| 273 | GAD2 | Q05329 | Glutamate Decarboxylase 2 | GeneCard |
| 274 | GBA | P04062 | Zinc finger X-chromosomal protein | OMIM |
| 275 | GBA3 | Q9H227 | Cytosolic beta-glucosidase | CTD |
| 276 | GBP1 | P32455 | Guanylate-binding protein 1 | CTD |
| 277 | GC | P02774 | GC Vitamin D Binding Protein | GeneCard/OMIM |
| 278 | GCG | P01275 | Glucagon | CTD |
| 279 | GCM1 | Q9NP62 | Chorion-specific transcription factor GCMa | CTD |
| 280 | GDE1 | Q9NZC3 | Glycerophosphodiester phosphodiesterase 1 | CTD |
| 281 | GFAP | P14136 | Glial fibrillary acidic protein | CTD |
| 282 | GGT3P | A6NGU5 | Gamma-Glutamyltransferase 3 Pseudogene | GeneCard |
| 283 | GGTA1P | Q4G0N0 | Inactive N-acetyllactosaminide alpha-1,3-galactosyltransferase | CTD |
| 284 | GH1 | P01241 | Growth Hormone 1 | GeneCard |
| 285 | GLYAT | Q6IB77 | Glycine N-acyltransferase | CTD |
| 286 | GNAS | P84996 | GNAS Complex Locus | GeneCard |
| 287 | GNAT3 | A8MTJ3 | Guanine nucleotide-binding protein G | CTD |
| 288 | GNB3 | P16520 | Guanine nucleotide-binding protein G | CTD |
| 289 | GNL1 | P36915 | G Protein Nucleolar 1 (Putative) | GeneCard |
| 290 | GOLGA8A | A7E2F4 | Golgin subfamily A member 8A | CTD |
| 291 | GOT1L1 | Q8NHS2 | Putative aspartate aminotransferase, cytoplasmic 2 | CTD |
| 292 | GPM6B | Q13491 | Neuronal membrane glycoprotein M6-b | CTD |
| 293 | GPR1 | P46091 | G-protein coupled receptor 1 | CTD |
| 294 | GPR174 | Q9BXC1 | G Protein-Coupled Receptor 174 | GeneCard |
| 295 | GPR18 | Q14330 | N-arachidonyl glycine receptor | CTD |
| 296 | GPR183 | P32249 | G-protein coupled receptor 183 | CTD |
| 297 | GPT | P24298 | Glutamic--Pyruvic Transaminase | GeneCard |
| 298 | GPX1 | P07203 | Glutathione peroxidase 1 | CTD |
| 299 | GRSF1 | Q12849 | G-rich sequence factor 1 | CTD |
| 300 | GSDME | O60443 | Gasdermin-E | CTD |
| 301 | GSR | P00390 | Glutathione-Disulfide Reductase | GeneCard |
| 302 | GSTM1 | P09488 | Glutathione S-transferase Mu 1 | CTD/GeneCard |
| 303 | GSTP1 | P09211 | Glutathione S-Transferase Pi 1 | GeneCard |
| 304 | GSTT1 | P30711 | Glutathione S-Transferase Theta 1 | GeneCard |
| 305 | GYG1 | P46976 | Glycogenin-1 | CTD |
| 306 | HAPLN2 | Q9GZV7 | Hyaluronan and proteoglycan link protein 2 | CTD |
| 307 | HAS1 | Q92839 | Hyaluronan Synthase 1 | GeneCard |
| 308 | HAS2 | Q92819 | Hyaluronan Synthase 2 | GeneCard |
| 309 | HAS3 | O00219 | Hyaluronan Synthase 3 | GeneCard |
| 310 | HAVCR2 | Q8TDQ0 | Hepatitis A Virus Cellular Receptor 2 | GeneCard |
| 311 | HBB | P68871 | Hemoglobin subunit beta | OMIM |
| 312 | HERC5 | Q9UII4 | E3 ISG15--protein ligase HERC5 | CTD |
| 313 | HERC6 | Q8IVU3 | Probable E3 ubiquitin-protein ligase HERC6 | CTD |
| 314 | HEXA | P06865 | Hexosaminidase Subunit Alpha | GeneCard |
| 315 | HEXB | P07686 | Hexosaminidase Subunit Beta | GeneCard |
| 316 | HIF1A | Q16665 | Hypoxia-inducible factor 1-alpha | CTD |
| 317 | HILPDA | Q9Y5L2 | Hypoxia-inducible lipid droplet-associated protein | CTD |
| 318 | HLA-A | P30450 | Major Histocompatibility Complex, Class I, A | GeneCard |
| 319 | HLA-B | Q29836 | Major Histocompatibility Complex, Class I, B | GeneCard |
| 320 | HLA-DQA1 | P01909 | Major Histocompatibility Complex, Class II, DQ Alpha 1 | GeneCard |
| 321 | HLA-DQB1 | P01920 | Major Histocompatibility Complex, Class II, DQ Beta 1 | GeneCard |
| 322 | HLA-DRA | P01903 | HLA class II histocompatibility antigen, DR alpha chain | CTD |
| 323 | HLA-DRB1 | Q5Y7A7 | Major Histocompatibility Complex, Class II, DR Beta 1 | GeneCard |
| 324 | HLA-DRB3 | P79483 | Major Histocompatibility Complex, Class II, DR Beta 3 | GeneCard |
| 325 | HLX | Q14774 | H2.0-like homeobox protein | CTD |
| 326 | HMGA2 | P52926 | High Mobility Group AT-Hook 2 | GeneCard |
| 327 | HMOX1 | P09601 | Heme oxygenase 1 | CTD |
| 328 | HNF1A | P20823 | Hepatocyte nuclear factor 1-alpha | OMIM |
| 329 | HSD11B1 | P28845 | Corticosteroid 11-beta-dehydrogenase isozyme 1 | CTD/Drugbank/GeneCard |
| 330 | HSD11B2 | P80365 | Corticosteroid 11-beta-dehydrogenase isozyme 2 | CTD |
| 331 | HSD17B10 | Q99714 | Hydroxysteroid 17-Beta Dehydrogenase 10 | GeneCard |
| 332 | HSF4 | Q9ULV5 | Heat shock factor protein 4 | OMIM |
| 333 | HSPA1A | P0DMV8 | Heat Shock Protein Family A (Hsp70) Member 1A | GeneCard |
| 334 | HSPB2 | Q16082 | Heat Shock Protein Family B (Small) Member 2 | GeneCard |
| 335 | HTR3A | P46098 | 5-hydroxytryptamine receptor 3A | CTD |
| 336 | IAPP | P10997 | Islet amyloid polypeptide | CTD |
| 337 | ICAM1 | P05362 | Intercellular Adhesion Molecule 1 | GeneCard/OMIM |
| 338 | ICAM3 | P32942 | Intercellular adhesion molecule 3 | CTD |
| 339 | IFI27 | P40305 | Interferon alpha-inducible protein 27, mitochondrial | CTD |
| 340 | IFI35 | P80217 | Interferon-induced 35 kDa protein | CTD |
| 341 | IFI44 | Q8TCB0 | Interferon-induced protein 44 | CTD |
| 342 | IFI44L | Q53G44 | Interferon-induced protein 44-like | CTD |
| 343 | IFI6 | P09912 | Interferon alpha-inducible protein 6 | CTD |
| 344 | IFIH1 | Q9BYX4 | Interferon-induced helicase C domain-containing protein 1 | CTD/GeneCard/OMIM |
| 345 | IFIT1 | P09914 | Interferon-induced protein with tetratricopeptide repeats 1 | CTD |
| 346 | IFIT3 | O14879 | Interferon-induced protein with tetratricopeptide repeats 3 | CTD |
| 347 | IFNAR2 | P48551 | Interferon alpha/beta receptor 2 | OMIM |
| 348 | IFNB1 | P01574 | Interferon beta | OMIM |
| 349 | IFNG | P01579 | Interferon gamma | CTD/GeneCard |
| 350 | IGF1 | P05019 | Insulin Like Growth Factor 1 | GeneCard |
| 351 | IGF1R | P08069 | Insulin-like growth factor 1 receptor | Drugbank/GeneCard |
| 352 | IGF2 | P01344 | Insulin Like Growth Factor 2 | GeneCard |
| 353 | IGFBP1 | P08833 | Insulin Like Growth Factor Binding Protein 1 | GeneCard |
| 354 | IGFBP2 | P18065 | Insulin Like Growth Factor Binding Protein 2 | GeneCard |
| 355 | IGFBP3 | P17936 | Insulin Like Growth Factor Binding Protein 3 | GeneCard |
| 356 | IGHE | P01854 | Immunoglobulin Heavy Constant Epsilon | GeneCard |
| 357 | IGKC | P01834 | Immunoglobulin kappa constant | CTD |
| 358 | IL10 | P22301 | Interleukin-10 | CTD/GeneCard |
| 359 | IL10RB | Q08334 | Interleukin-10 receptor subunit beta | OMIM |
| 360 | IL12A | P29459 | Interleukin-12 subunit alpha | CTD/GeneCard |
| 361 | IL12B | P29460 | Interleukin-12 subunit beta | CTD/GeneCard |
| 362 | IL13 | P35225 | Interleukin-13 | CTD/GeneCard/OMIM |
| 363 | IL16 | Q14005 | Interleukin 16 | GeneCard |
| 364 | IL17A | Q16552 | Interleukin 17A | GeneCard |
| 365 | IL17D | Q8TAD2 | Interleukin-17D | CTD |
| 366 | IL17F | Q96PD4 | Interleukin 17F | GeneCard |
| 367 | IL18 | Q14116 | Interleukin 18 | GeneCard |
| 368 | IL18RAP | O95256 | Interleukin-18 receptor accessory protein | CTD |
| 369 | IL1A | P01583 | Interleukin 1 Alpha | GeneCard |
| 370 | IL1B | P01584 | Interleukin-1 beta | CTD/GeneCard |
| 371 | IL1R1 | P14778 | Interleukin 1 Receptor Type 1 | GeneCard |
| 372 | IL1RAPL2 | Q9NP60 | Interleukin 1 Receptor Accessory Protein Like 2 | GeneCard |
| 373 | IL1RN | P18510 | Interleukin-1 receptor antagonist protein | CTD/GeneCard |
| 374 | IL2 | P60568 | Interleukin 2 | GeneCard |
| 375 | IL21 | Q9HBE4 | Interleukin 21 | GeneCard |
| 376 | IL22RA1 | Q8N6P7 | Interleukin-22 receptor subunit alpha-1 | CTD |
| 377 | IL23A | Q9NPF7 | Interleukin-23 subunit alpha | CTD |
| 378 | IL23R | Q5VWK5 | Interleukin 23 Receptor | GeneCard |
| 379 | IL2RA | P01589 | Interleukin 2 Receptor Subunit Alpha | GeneCard |
| 380 | IL2RB | P14784 | Interleukin 2 Receptor Subunit Beta | GeneCard |
| 381 | IL3 | P08700 | Interleukin 3 | GeneCard |
| 382 | IL4 | P05112 | Interleukin-4 | CTD/GeneCard/OMIM |
| 383 | IL4R | P24394 | Interleukin-4 receptor subunit alpha | CTD |
| 384 | IL5 | P05113 | Interleukin-5 | CTD/GeneCard |
| 385 | IL6 | P05231 | Interleukin-6 | CTD/GeneCard |
| 386 | IL7 | P13232 | Interleukin 7 | GeneCard |
| 387 | IL9 | P15248 | Interleukin 9 | GeneCard |
| 388 | IMMT | Q16891 | MICOS complex subunit MIC60 | CTD |
| 389 | INHBC | P55103 | Inhibin beta C chain | CTD |
| 390 | INPP5F | Q9Y2H2 | Phosphatidylinositide phosphatase SAC2 | CTD |
| 391 | INS | P01308 | Insulin | CTD/GeneCard |
| 392 | IPCEF1 | Q8WWN9 | Interactor protein for cytohesin exchange factors 1 | CTD |
| 393 | IRF1 | P10914 | Interferon regulatory factor 1 | OMIM |
| 394 | IRF5 | Q13568 | Interferon Regulatory Factor 5 | GeneCard |
| 395 | IRF7 | Q92985 | Interferon regulatory factor 7 | CTD |
| 396 | ISG15 | P05161 | Ubiquitin-like protein ISG15 | CTD |
| 397 | ISG20 | Q96AZ6 | Interferon-stimulated gene 20 kDa protein | CTD |
| 398 | ITGA4 | P13612 | Integrin Subunit Alpha 4 | GeneCard |
| 399 | ITGAE | P38570 | Integrin Subunit Alpha E | GeneCard |
| 400 | ITGAL | P20701 | Integrin Subunit Alpha L | GeneCard |
| 401 | ITGAX | P20702 | Integrin Subunit Alpha X | GeneCard |
| 402 | ITGB2 | P05107 | Integrin Subunit Beta 2 | GeneCard |
| 403 | IYD | Q6PHW0 | Iodotyrosine deiodinase 1 | CTD |
| 404 | JAK1 | P23458 | Tyrosine-protein kinase JAK1 | CTD |
| 405 | JAK2 | O60674 | Janus Kinase 2 | GeneCard |
| 406 | JUN | P05412 | Transcription factor AP-1 | CTD |
| 407 | KCNE1 | P15382 | Potassium Voltage-Gated Channel Subfamily E Regulatory Subunit 1 | GeneCard |
| 408 | KCNE3 | Q9Y6H6 | Potassium Voltage-Gated Channel Subfamily E Regulatory Subunit 3 | GeneCard/OMIM |
| 409 | KCNG3 | Q8TAE7 | Potassium voltage-gated channel subfamily G member 3 | CTD |
| 410 | KCNJ18 | B7U540 | Potassium Inwardly Rectifying Channel Subfamily J Member 18 | GeneCard |
| 411 | KCNJ2 | P63252 | Potassium Inwardly Rectifying Channel Subfamily J Member 2 | GeneCard |
| 412 | KCNK3 | O14649 | Potassium channel subfamily K member 3 | CTD |
| 413 | KCNMB2 | Q9Y691 | Calcium-activated potassium channel subunit beta-2 | CTD |
| 414 | KIAA0040 | Q15053 | Uncharacterized protein KIAA0040 | CTD |
| 415 | KIF12 | Q96FN5 | Kinesin-like protein KIF12 | CTD |
| 416 | KIFC2 | Q96AC6 | Kinesin-like protein KIFC2 | CTD |
| 417 | KLRK1 | P26718 | Killer Cell Lectin Like Receptor K1 | GeneCard |
| 418 | KRT20 | P35900 | Keratin, type I cytoskeletal 20 | CTD |
| 419 | KRT79 | Q5XKE5 | Keratin, type II cytoskeletal 79 | CTD |
| 420 | LACRT | Q9GZZ8 | Lacritin | GeneCard |
| 421 | LAIR1 | Q6GTX8 | Leukocyte-associated immunoglobulin-like receptor 1 | CTD |
| 422 | LAMA3 | Q16787 | Laminin subunit alpha-3 | OMIM |
| 423 | LAMB1 | P07942 | Laminin subunit beta-1 | CTD |
| 424 | LAMB2 | P55268 | Laminin subunit beta-2 | CTD |
| 425 | LAMB3 | Q13751 | Laminin subunit beta-3 | CTD |
| 426 | LELP1 | Q5T871 | Late cornified envelope-like proline-rich protein 1 | CTD |
| 427 | LEP | P41159 | Leptin | GeneCard |
| 428 | LETMD1 | Q6P1Q0 | LETM1 domain-containing protein 1 | CTD |
| 429 | LGALS9 | O00182 | Galectin 9 | GeneCard |
| 430 | LILRA1 | O75019 | Leukocyte immunoglobulin-like receptor subfamily A member 1 | CTD |
| 431 | LILRB1 | Q8NHL6 | Leukocyte Immunoglobulin Like Receptor B1 | GeneCard |
| 432 | LILRB4 | Q8NHJ6 | Leukocyte Immunoglobulin Like Receptor B4 | GeneCard |
| 433 | LINGO4 | Q6UY18 | Leucine-rich repeat and immunoglobulin-like domain-containing nogo receptor-interacting protein 4 | CTD |
| 434 | LMOD1 | P29536 | Leiomodin 1 | GeneCard/OMIM |
| 435 | LMOD3 | Q0VAK6 | Leiomodin 3 | GeneCard |
| 436 | LONP2 | Q86WA8 | Lon protease homolog 2, peroxisomal | CTD |
| 437 | LPL | P06858 | Lipoprotein Lipase | GeneCard |
| 438 | LRP2 | P98164 | LDL Receptor Related Protein 2 | GeneCard/OMIM |
| 439 | LRRC8A | Q8IWT6 | Volume-regulated anion channel subunit LRRC8A | CTD |
| 440 | LTA | P01374 | Lymphotoxin Alpha | GeneCard |
| 441 | LY6E | Q16553 | Lymphocyte antigen 6E | CTD |
| 442 | LYRM1 | O43325 | LYR motif-containing protein 1 | CTD |
| 443 | LYZ | P61626 | Lysozyme | GeneCard |
| 444 | LYZL1 | Q6UWQ5 | Lysozyme-like protein 1 | CTD |
| 445 | MACROD2 | A1Z1Q3 | Mono-ADP Ribosylhydrolase 2 | GeneCard |
| 446 | MAN1C1 | Q9NR34 | Mannosyl-oligosaccharide 1,2-alpha-mannosidase IC | CTD |
| 447 | MAP2K1 | Q02750 | Dual specificity mitogen-activated protein kinase kinase 1 | OMIM |
| 448 | MAPK1 | P28482 | Mitogen-activated protein kinase 1 | CTD |
| 449 | MAPK3 | P27361 | Mitogen-activated protein kinase 3 | CTD/GeneCard |
| 450 | MAPK8IP2 | Q13387 | C-Jun-amino-terminal kinase-interacting protein 2 | CTD |
| 451 | MAPRE3 | Q9UPY8 | Microtubule-associated protein RP/EB family member 3 | CTD |
| 452 | MAPT | P10636 | Microtubule-associated protein tau | OMIM |
| 453 | MARCKS | P29966 | Myristoylated alanine-rich C-kinase substrate | CTD |
| 454 | MARK3 | P27448 | MAP/microtubule affinity-regulating kinase 3 | OMIM |
| 455 | MATN3 | O15232 | Matrilin 3 | GeneCard |
| 456 | MB21D2 | Q8IYB1 | Protein MB21D2 | CTD |
| 457 | MBP | P02686 | Myelin basic protein | CTD |
| 458 | MC4R | P32245 | Melanocortin receptor 4 | CTD |
| 459 | MCRIP2 | Q9BUT9 | MAPK regulated corepressor interacting protein 2 | CTD |
| 460 | MCUR1 | Q96AQ8 | Mitochondrial calcium uniporter regulator 1 | CTD |
| 461 | MGAT4C | Q9UBM8 | Alpha-1,3-mannosyl-glycoprotein 4-beta-N-acetylglucosaminyltransferase C | CTD |
| 462 | MID1 | O15344 | E3 ubiquitin-protein ligase Midline-1 | OMIM |
| 463 | MKRN1 | Q9UHC7 | E3 ubiquitin-protein ligase makorin-1 | OMIM |
| 464 | MLYCD | O95822 | Malonyl-CoA decarboxylase, mitochondrial | CTD |
| 465 | MME | P08473 | Membrane Metalloendopeptidase | GeneCard |
| 466 | MMP10 | P09238 | Stromelysin-2 | CTD |
| 467 | MMP12 | P39900 | Macrophage metalloelastase | CTD |
| 468 | MMP3 | P08254 | Matrix Metallopeptidase 3 | GeneCard |
| 469 | MMP9 | P14780 | Matrix Metallopeptidase 9 | GeneCard |
| 470 | MOG | Q16653 | Myelin-oligodendrocyte glycoprotein | CTD |
| 471 | MOGAT3 | Q86VF5 | 2-acylglycerol O-acyltransferase 3 | CTD |
| 472 | MPO | P05164 | Myeloperoxidase | CTD/GeneCard |
| 473 | MRPL14 | Q6P1L8 | 39S ribosomal protein L14, mitochondrial | CTD |
| 474 | MRPL15 | Q9P015 | 39S ribosomal protein L15, mitochondrial | CTD |
| 475 | MRPL16 | Q9NX20 | 39S ribosomal protein L16, mitochondrial | CTD |
| 476 | MRPL18 | Q9H0U6 | 39S ribosomal protein L18, mitochondrial | CTD |
| 477 | MRPL2 | Q5T653 | 39S ribosomal protein L2, mitochondrial | CTD |
| 478 | MRPL22 | Q9NWU5 | 39S ribosomal protein L22, mitochondrial | CTD |
| 479 | MRPL24 | Q96A35 | 39S ribosomal protein L24, mitochondrial | CTD |
| 480 | MRPL30 | Q8TCC3 | 39S ribosomal protein L30, mitochondrial | CTD |
| 481 | MRPL34 | Q9BQ48 | 39S ribosomal protein L34, mitochondrial | CTD |
| 482 | MRPL35 | Q9NZE8 | 39S ribosomal protein L35, mitochondrial | CTD |
| 483 | MRPL37 | Q9BZE1 | 39S ribosomal protein L37, mitochondrial | CTD |
| 484 | MRPL4 | Q9BYD3 | 39S ribosomal protein L4, mitochondrial | CTD |
| 485 | MRPL40 | Q9NQ50 | 39S ribosomal protein L40, mitochondrial | CTD |
| 486 | MRPL43 | Q8N983 | 39S ribosomal protein L43, mitochondrial | CTD |
| 487 | MRPL44 | Q9H9J2 | 39S ribosomal protein L44, mitochondrial | CTD |
| 488 | MRPL45 | Q9BRJ2 | 39S ribosomal protein L45, mitochondrial | CTD |
| 489 | MRPL46 | Q9H2W6 | 39S ribosomal protein L46, mitochondrial | CTD |
| 490 | MRPL47 | Q9HD33 | 39S ribosomal protein L47, mitochondrial | CTD |
| 491 | MRPL48 | Q96GC5 | 39S ribosomal protein L48, mitochondrial | CTD |
| 492 | MRPL49 | Q13405 | 39S ribosomal protein L49, mitochondrial | CTD |
| 493 | MRPL51 | Q4U2R6 | 39S ribosomal protein L51, mitochondrial | CTD |
| 494 | MRPL52 | Q86TS9 | 39S ribosomal protein L52, mitochondrial | CTD |
| 495 | MRPL53 | Q96EL3 | 39S ribosomal protein L53, mitochondrial | CTD |
| 496 | MRPL55 | Q7Z7F7 | 39S ribosomal protein L55, mitochondrial | CTD |
| 497 | MRPL57 | Q9BQC6 | Ribosomal protein 63, mitochondrial | CTD |
| 498 | MRPL9 | Q9BYD2 | 39S ribosomal protein L9, mitochondrial | CTD |
| 499 | MRPS11 | P82912 | 28S ribosomal protein S11, mitochondrial | CTD |
| 500 | MRPS14 | O60783 | 28S ribosomal protein S14, mitochondrial | CTD |
| 501 | MRPS15 | P82914 | 28S ribosomal protein S15, mitochondrial | CTD |
| 502 | MRPS16 | Q9Y3D3 | 28S ribosomal protein S16, mitochondrial | CTD |
| 503 | MRPS17 | Q9Y2R5 | 28S ribosomal protein S17, mitochondrial | CTD |
| 504 | MRPS18A | Q9NVS2 | 39S ribosomal protein S18a, mitochondrial | CTD |
| 505 | MRPS2 | Q9Y399 | 28S ribosomal protein S2, mitochondrial | CTD |
| 506 | MRPS21 | P82921 | 28S ribosomal protein S21, mitochondrial | CTD |
| 507 | MRPS23 | Q9Y3D9 | 28S ribosomal protein S23, mitochondrial | CTD |
| 508 | MRPS25 | P82663 | 28S ribosomal protein S25, mitochondrial | CTD |
| 509 | MRPS26 | Q9BYN8 | 28S ribosomal protein S26, mitochondrial | CTD |
| 510 | MRPS28 | Q9Y2Q9 | 28S ribosomal protein S28, mitochondrial | CTD |
| 511 | MRPS34 | P82930 | 28S ribosomal protein S34, mitochondrial | CTD |
| 512 | MRPS36 | P82909 | 28S ribosomal protein S36, mitochondrial | CTD |
| 513 | MRPS5 | P82675 | 28S ribosomal protein S5, mitochondrial | CTD |
| 514 | MRPS9 | P82933 | 28S ribosomal protein S9, mitochondrial | CTD |
| 515 | MS4A1 | P11836 | Membrane Spanning 4-Domains A1 | GeneCard |
| 516 | MS4A10 | Q96PG2 | Membrane-spanning 4-domains subfamily A member 10 | CTD |
| 517 | MSC | O60682 | Musculin | CTD |
| 518 | MT-ATP6 | P00846 | ATP synthase subunit a | OMIM |
| 519 | MTHFR | P42898 | Methylenetetrahydrofolate Reductase | GeneCard |
| 520 | MUC13 | Q9H3R2 | Mucin-13 | CTD |
| 521 | MUC5AC | P98088 | Mucin-5AC | CTD |
| 522 | MX1 | P20591 | Interferon-induced GTP-binding protein Mx1 | CTD |
| 523 | MYH2 | Q9UKX2 | Myosin-2 | CTD |
| 524 | MYH4 | Q9Y623 | Myosin-4 | CTD |
| 525 | MYOD1 | P15172 | Myoblast determination protein 1 | CTD |
| 526 | MYORG | Q6NSJ0 | Myogenesis-regulating glycosidase | CTD |
| 527 | NALCN | Q8IZF0 | Sodium leak channel non-selective protein | CTD |
| 528 | NAMPT | P43490 | Nicotinamide Phosphoribosyltransferase | GeneCard |
| 529 | NAPEPLD | Q6IQ20 | N-acyl-phosphatidylethanolamine-hydrolyzing phospholipase D | CTD |
| 530 | NCOA6 | Q14686 | Nuclear receptor coactivator 6 | CTD |
| 531 | NDN | Q99608 | Necdin | CTD |
| 532 | NDUFA10 | O95299 | NADH dehydrogenase [ubiquinone] 1 alpha subcomplex subunit 10, mitochondrial | CTD |
| 533 | NDUFA11 | Q86Y39 | NADH dehydrogenase [ubiquinone] 1 alpha subcomplex subunit 11 | CTD |
| 534 | NDUFA4L2 | Q9NRX3 | NADH dehydrogenase [ubiquinone] 1 alpha subcomplex subunit 4-like 2 | CTD |
| 535 | NDUFA6 | P56556 | NADH dehydrogenase [ubiquinone] 1 alpha subcomplex subunit 6 | CTD |
| 536 | NDUFAF2 | Q8N183 | NADH dehydrogenase [ubiquinone] 1 alpha subcomplex assembly factor 2 | CTD |
| 537 | NDUFB10 | O96000 | NADH dehydrogenase [ubiquinone] 1 beta subcomplex subunit 10 | CTD |
| 538 | NDUFB11 | Q9NX14 | NADH dehydrogenase [ubiquinone] 1 beta subcomplex subunit 11, mitochondrial | CTD |
| 539 | NDUFB4 | O95168 | NADH dehydrogenase [ubiquinone] 1 beta subcomplex subunit 4 | CTD |
| 540 | NDUFB7 | P17568 | NADH dehydrogenase [ubiquinone] 1 beta subcomplex subunit 7 | CTD |
| 541 | NDUFB9 | Q9Y6M9 | NADH dehydrogenase [ubiquinone] 1 beta subcomplex subunit 9 | CTD |
| 542 | NDUFC1 | O43677 | NADH dehydrogenase [ubiquinone] 1 subunit C1, mitochondrial | CTD |
| 543 | NEB | P20929 | Nebulin | GeneCard |
| 544 | NEU1 | Q99519 | Neuraminidase 1 | GeneCard |
| 545 | NEXN | Q0ZGT2 | Nexilin F-Actin Binding Protein | GeneCard |
| 546 | NFE2L2 | Q16236 | Nuclear factor erythroid 2-related factor 2 | CTD |
| 547 | NFKB1 | P19838 | Nuclear Factor Kappa B Subunit 1 | GeneCard |
| 548 | NGF | P01138 | Nerve Growth Factor | GeneCard |
| 549 | NID2 | Q14112 | Nidogen-2 | CTD |
| 550 | NKX2-1 | P43699 | NK2 Homeobox 1 | GeneCard |
| 551 | NMB | P08949 | Neuromedin-B | CTD |
| 552 | NOD1 | Q9Y239 | Nucleotide-binding oligomerization domain-containing protein 1 | OMIM |
| 553 | NOS2 | P35228 | Nitric oxide synthase, inducible | CTD |
| 554 | NOTCH1 | P46531 | Neurogenic locus notch homolog protein 1 | CTD |
| 555 | NOTCH2 | Q04721 | Notch Receptor 2 | GeneCard |
| 556 | NPHS1 | O60500 | Nephrin | CTD |
| 557 | NPHS2 | Q9NP85 | Podocin | CTD |
| 558 | NR3C1 | P04150 | Glucocorticoid receptor | CTD/Drugbank/GeneCard |
| 559 | NR4A3 | Q92570 | Nuclear receptor subfamily 4 group A member 3 | CTD |
| 560 | NR5A1 | Q13285 | Nuclear Receptor Subfamily 5 Group A Member 1 | GeneCard |
| 561 | OAS1 | P00973 | 2'-5'-oligoadenylate synthase 1 | CTD |
| 562 | OAS3 | Q9Y6K5 | 2'-5'-oligoadenylate synthase 3 | CTD |
| 563 | OASL | Q15646 | 2'-5'-oligoadenylate synthase-like protein | CTD |
| 564 | OIT3 | Q8WWZ8 | Oncoprotein-induced transcript 3 protein | CTD |
| 565 | OMA1 | Q96E52 | Metalloendopeptidase OMA1, mitochondrial | CTD |
| 566 | ORAI3 | Q9BRQ5 | Protein orai-3 | CTD |
| 567 | OSBPL5 | Q9H0X9 | Oxysterol-binding protein-related protein 5 | CTD |
| 568 | OSTN | P61366 | Osteocrin | CTD |
| 569 | PACC1 | Q9H813 | Proton-activated chloride channel | CTD |
| 570 | PALD1 | Q9ULE6 | Paladin | CTD |
| 571 | PARP1 | P09874 | Poly(ADP-Ribose) Polymerase 1 | GeneCard |
| 572 | PAX8 | Q06710 | Paired Box 8 | GeneCard |
| 573 | PBLD | P30039 | Phenazine biosynthesis-like domain-containing protein | CTD |
| 574 | PCBD1 | P61457 | Pterin-4-alpha-carbinolamine dehydratase | OMIM |
| 575 | PDCD4 | Q53EL6 | Programmed Cell Death 4 | GeneCard |
| 576 | PDGFA | P04085 | Platelet Derived Growth Factor Subunit A | GeneCard |
| 577 | PDGFB | P01127 | Platelet Derived Growth Factor Subunit B | GeneCard |
| 578 | PDGFRA | P16234 | Platelet Derived Growth Factor Receptor Alpha | GeneCard |
| 579 | PDHA1 | P08559 | Pyruvate dehydrogenase E1 component subunit alpha, somatic form, mitochondrial | OMIM |
| 580 | PHETA1 | Q8N4B1 | Sesquipedalian-1 | CTD |
| 581 | PHF6 | Q8IWS0 | PHD finger protein 6 | OMIM |
| 582 | PHGDH | O43175 | D-3-phosphoglycerate dehydrogenase | CTD |
| 583 | PLA1A | Q53H76 | Phospholipase A1 member A | CTD |
| 584 | PLA2G12B | Q9BX93 | Group XIIB secretory phospholipase A2-like protein | CTD |
| 585 | PLA2G4A | P47712 | Cytosolic phospholipase A2 | CTD |
| 586 | PLA2G6 | O60733 | Phospholipase A2 Group VI | GeneCard |
| 587 | PLAT | P00750 | Tissue-type plasminogen activator | CTD |
| 588 | PLCXD1 | Q9NUJ7 | PI-PLC X domain-containing protein 1 | CTD |
| 589 | PLEKHO1 | Q53GL0 | Pleckstrin homology domain-containing family O member 1 | CTD |
| 590 | PLPP3 | O14495 | Phospholipid phosphatase 3 | CTD |
| 591 | PLSCR2 | Q9NRY7 | Phospholipid scramblase 2 | CTD |
| 592 | POLA1 | P09884 | DNA polymerase alpha catalytic subunit | OMIM |
| 593 | POLR1E | Q9GZS1 | DNA-directed RNA polymerase I subunit RPA49 | CTD |
| 594 | POMC | P01189 | Proopiomelanocortin | GeneCard |
| 595 | PPARA | Q07869 | Peroxisome Proliferator Activated Receptor Alpha | GeneCard |
| 596 | PPARG | P37231 | Peroxisome Proliferator Activated Receptor Gamma | GeneCard/OMIM |
| 597 | PPARGC1A | Q9UBK2 | Peroxisome proliferator-activated receptor gamma coactivator 1-alpha | OMIM |
| 598 | PPIL6 | Q8IXY8 | Probable inactive peptidyl-prolyl cis-trans isomerase-like 6 | CTD |
| 599 | PRAM1 | Q96QH2 | PML-RARA-regulated adapter molecule 1 | CTD |
| 600 | PRELP | P51888 | Prolargin | CTD |
| 601 | PRM2 | P04554 | Protamine-2 | CTD |
| 602 | PROZ | P22891 | Vitamin K-dependent protein Z | CTD |
| 603 | PSMB9 | P28065 | Proteasome 20S Subunit Beta 9 | GeneCard |
| 604 | PTEN | P60484 | Phosphatase And Tensin Homolog | GeneCard |
| 605 | PTGER2 | P43116 | Prostaglandin E Receptor 2 | GeneCard |
| 606 | PTGER4 | P35408 | Prostaglandin E2 receptor EP4 subtype | CTD |
| 607 | PTGS2 | P35354 | Prostaglandin-Endoperoxide Synthase 2 | GeneCard/CTD |
| 608 | PTPN12 | Q05209 | Protein Tyrosine Phosphatase Non-Receptor Type 12 | GeneCard |
| 609 | PTPN22 | Q9Y2R2 | Protein Tyrosine Phosphatase Non-Receptor Type 22 | GeneCard/OMIM |
| 610 | PTPN3 | P26045 | Protein Tyrosine Phosphatase Non-Receptor Type 3 | GeneCard |
| 611 | PTPRC | P08575 | Wolframin | OMIM |
| 612 | PTS | Q03393 | 6-Pyruvoyltetrahydropterin Synthase | GeneCard |
| 613 | PTX3 | P26022 | Pentraxin 3 | GeneCard |
| 614 | PYGM | P11217 | Glycogen phosphorylase, muscle form | CTD |
| 615 | QRFPR | Q96P65 | Pyroglutamylated RF-amide peptide receptor | CTD |
| 616 | RAB15 | P59190 | Ras-related protein Rab-15 | CTD |
| 617 | RAB19 | A4D1S5 | Ras-related protein Rab-19 | CTD |
| 618 | RAB1B | Q9H0U4 | Ras-related protein Rab-1B | CTD |
| 619 | RANBP2 | P49792 | E3 SUMO-protein ligase RanBP2 | CTD |
| 620 | RARA | P10276 | Retinoic Acid Receptor Alpha | GeneCard |
| 621 | RB1 | P06400 | Retinoblastoma-associated protein | CTD |
| 622 | RBKS | Q9H477 | Ribokinase | CTD |
| 623 | RBM25 | P49756 | RNA Binding Motif Protein 25 | GeneCard |
| 624 | RBMX | P38159 | RNA-binding motif protein, X chromosome | OMIM |
| 625 | RBMY1A1 | P0DJD3 | RNA-binding motif protein, Y chromosome, family 1 member A1 | OMIM |
| 626 | RCOR2 | Q8IZ40 | REST corepressor 2 | CTD |
| 627 | REC8 | O95072 | Meiotic recombination protein REC8 homolog | CTD |
| 628 | REEP3 | Q6NUK4 | Receptor expression-enhancing protein 3 | CTD |
| 629 | REG4 | Q9BYZ8 | Regenerating islet-derived protein 4 | CTD |
| 630 | RELA | Q04206 | Transcription factor p65 | CTD |
| 631 | REPS2 | Q8NFH8 | RalBP1-associated Eps domain-containing protein 2 | CTD |
| 632 | RFXAP | O00287 | Regulatory factor X-associated protein | CTD |
| 633 | RGS4 | P49798 | Regulator of G-protein signaling 4 | CTD |
| 634 | RIMS2 | Q9UQ26 | Regulating synaptic membrane exocytosis protein 2 | CTD |
| 635 | RINL | Q6ZS11 | Ras and Rab interactor-like protein | CTD |
| 636 | RIOX1 | Q9H6W3 | Ribosomal oxygenase 1 | CTD |
| 637 | RIPK2 | O43353 | Receptor-interacting serine/threonine-protein kinase 2 | CTD |
| 638 | RNASE3 | P12724 | Eosinophil cationic protein | CTD |
| 639 | RNF19B | Q6ZMZ0 | E3 ubiquitin-protein ligase RNF19B | CTD |
| 640 | RPS6KA4 | O75676 | Ribosomal protein S6 kinase alpha-4 | CTD |
| 641 | RPUSD2 | Q8IZ73 | RNA pseudouridylate synthase domain-containing protein 2 | CTD |
| 642 | RSAD2 | Q8WXG1 | Radical S-adenosyl methionine domain-containing protein 2 | CTD |
| 643 | RXFP2 | Q8WXD0 | Relaxin receptor 2 | CTD |
| 644 | S1PR1 | P21453 | Sphingosine-1-Phosphate Receptor 1 | GeneCard |
| 645 | SCD | O00767 | Stearoyl-CoA Desaturase | GeneCard |
| 646 | SCN4A | P35499 | Sodium Voltage-Gated Channel Alpha Subunit 4 | GeneCard |
| 647 | SCRG1 | O75711 | Scrapie-responsive protein 1 | CTD |
| 648 | SDC1 | P18827 | Syndecan 1 | GeneCard |
| 649 | SDHA | P31040 | Succinate Dehydrogenase Complex Flavoprotein Subunit A | GeneCard |
| 650 | SDHB | P21912 | Succinate Dehydrogenase Complex Iron Sulfur Subunit B | GeneCard |
| 651 | SELE | P16581 | Selectin E | GeneCard |
| 652 | SELL | P14151 | Selectin L | GeneCard |
| 653 | SEMA3C | Q99985 | Semaphorin-3C | OMIM |
| 654 | SENP8 | Q96LD8 | Sentrin-specific protease 8 | CTD |
| 655 | SERPINA4 | P29622 | Kallistatin | CTD |
| 656 | SERPINA6 | P08185 | Corticosteroid-binding globulin | Drugbank |
| 657 | SERPINA7 | P05543 | Serpin Family A Member 7 | GeneCard/OMIM |
| 658 | SERPINC1 | P01008 | Antithrombin-III | CTD |
| 659 | SERPINE1 | P05121 | Serpin Family E Member 1 | GeneCard |
| 660 | SF1 | Q15637 | Splicing Factor 1 | GeneCard |
| 661 | SFRP1 | Q8N474 | Secreted Frizzled Related Protein 1 | GeneCard |
| 662 | SH3TC1 | Q8TE82 | SH3 domain and tetratricopeptide repeat-containing protein 1 | CTD |
| 663 | SHANK2 | Q9UPX8 | SH3 And Multiple Ankyrin Repeat Domains 2 | GeneCard |
| 664 | SHD | Q96IW2 | SH2 domain-containing adapter protein D | CTD |
| 665 | SIRPB1 | Q5TFQ8 | Signal-regulatory protein beta-1 isoform 3 | CTD |
| 666 | SIRT1 | Q96EB6 | NAD-dependent protein deacetylase sirtuin-1 | CTD |
| 667 | SIRT6 | Q8N6T7 | NAD-dependent protein deacetylase sirtuin-6 | CTD |
| 668 | SKAP1 | Q86WV1 | Src kinase-associated phosphoprotein 1 | CTD |
| 669 | SLAIN1 | Q8ND83 | SLAIN motif-containing protein 1 | CTD |
| 670 | SLC17A8 | Q8NDX2 | Vesicular glutamate transporter 3 | CTD |
| 671 | SLC22A10 | Q63ZE4 | Solute carrier family 22 member 10 | CTD |
| 672 | SLC22A18AS | Q8N1D0 | Beckwith-Wiedemann syndrome chromosomal region 1 candidate gene B protein | CTD |
| 673 | SLC24A2 | Q9UI40 | Sodium/potassium/calcium exchanger 2 | CTD |
| 674 | SLC25A16 | P16260 | Solute Carrier Family 25 Member 16 | GeneCard/OMIM |
| 675 | SLC25A24 | Q6NUK1 | Calcium-binding mitochondrial carrier protein SCaMC-1 | CTD |
| 676 | SLC25A26 | Q70HW3 | S-adenosylmethionine mitochondrial carrier protein | CTD |
| 677 | SLC25A34 | Q6PIV7 | Solute carrier family 25 member 34 | CTD |
| 678 | SLC25A42 | Q86VD7 | Mitochondrial coenzyme A transporter SLC25A42 | CTD |
| 679 | SLC26A11 | Q86WA9 | Sodium-independent sulfate anion transporter | CTD |
| 680 | SLC26A4 | O43511 | Pendrin | OMIM |
| 681 | SLC28A1 | O00337 | Sodium/nucleoside cotransporter 1 | CTD |
| 682 | SLC2A1 | P11166 | Solute carrier family 2, facilitated glucose transporter member 1 | OMIM |
| 683 | SLC2A14 | Q8TDB8 | Solute carrier family 2, facilitated glucose transporter member 14 | CTD |
| 684 | SLC43A3 | Q8NBI5 | Solute carrier family 43 member 3 | CTD |
| 685 | SLC5A5 | Q92911 | Complement C1q tumor necrosis factor-related protein 5 | OMIM |
| 686 | SLC6A18 | Q96N87 | Inactive sodium-dependent neutral amino acid transporter B | CTD |
| 687 | SLC7A10 | Q9NS82 | Asc-type amino acid transporter 1 | CTD |
| 688 | SLC7A11 | Q9UPY5 | Solute Carrier Family 7 Member 11 | GeneCard |
| 689 | SLCO1A2 | P46721 | Solute carrier organic anion transporter family member 1A2 | Drugbank |
| 690 | SLIT2 | O94813 | Slit Guidance Ligand 2 | GeneCard |
| 691 | SLITRK4 | Q8IW52 | SLIT and NTRK-like protein 4 | CTD |
| 692 | SMS | P52788 | Spermine synthase | CTD |
| 693 | SNAI3 | Q3KNW1 | Zinc finger protein SNAI3 | CTD |
| 694 | SNAP25 | P60880 | Synaptosomal-associated protein 25 | CTD |
| 695 | SNCA | P37840 | Alpha-synuclein | OMIM |
| 696 | SOCS3 | O14543 | Suppressor Of Cytokine Signaling 3 | GeneCard |
| 697 | SOD1 | P00441 | Superoxide Dismutase 1 | GeneCard |
| 698 | SOD2 | P04179 | Superoxide Dismutase 2 | GeneCard/CTD |
| 699 | SOHLH2 | Q9NX45 | Spermatogenesis- and oogenesis-specific basic helix-loop-helix-containing protein 2 | CTD |
| 700 | SOX3 | P41225 | Transcription factor SOX-3 | OMIM |
| 701 | SOX9 | P48436 | SRY-Box Transcription Factor 9 | GeneCard/OMIM |
| 702 | SPARC | P09486 | SPARC | OMIM |
| 703 | SPP2 | Q13103 | Secreted phosphoprotein 24 | CTD |
| 704 | SPRN | Q5BIV9 | Shadow of prion protein | OMIM |
| 705 | SPSB2 | Q99619 | SPRY domain-containing SOCS box protein 2 | CTD |
| 706 | SPTAN1 | Q13813 | Spectrin Alpha, Non-Erythrocytic 1 | GeneCard |
| 707 | SRGAP2 | O75044 | SLIT-ROBO Rho GTPase-activating protein 2 | OMIM |
| 708 | SRGAP2B | P0DMP2 | SLIT-ROBO Rho GTPase-activating protein 2B | OMIM |
| 709 | SRGAP2C | P0DJJ0 | SLIT-ROBO Rho GTPase-activating protein 2C | OMIM |
| 710 | SRL | Q86TD4 | Sarcalumenin | GeneCard |
| 711 | SRY | Q05066 | Sex-determining region Y protein | OMIM |
| 712 | SSMEM1 | Q8WWF3 | Serine-rich single-pass membrane protein 1 | CTD |
| 713 | SST | P61278 | Somatostatin | GeneCard |
| 714 | SSTR1 | P30872 | Somatostatin Receptor 1 | GeneCard |
| 715 | SSTR2 | P30874 | Somatostatin Receptor 2 | GeneCard |
| 716 | SSTR3 | P32745 | Somatostatin Receptor 3 | GeneCard |
| 717 | SSTR4 | P31391 | Somatostatin Receptor 4 | GeneCard |
| 718 | SSTR5 | P35346 | Somatostatin Receptor 5 | GeneCard |
| 719 | ST3GAL5 | Q9UNP4 | ST3 Beta-Galactoside Alpha-2,3-Sialyltransferase 5 | GeneCard |
| 720 | ST8SIA1 | Q92185 | ST8 Alpha-N-Acetyl-Neuraminide Alpha-2,8-Sialyltransferase 1 | GeneCard |
| 721 | STAMBP | O95630 | STAM-binding protein | CTD |
| 722 | STAT1 | P42224 | Signal transducer and activator of transcription 1-alpha/beta | CTD |
| 723 | STAT3 | P40763 | Signal Transducer And Activator Of Transcription 3 | GeneCard |
| 724 | STAT4 | Q14765 | Signal transducer and activator of transcription 4 | CTD |
| 725 | STAT6 | P42226 | Signal transducer and activator of transcription 6 | CTD |
| 726 | STS | P08842 | Steryl-sulfatase | OMIM/CTD |
| 727 | SURF1 | Q15526 | Surfeit locus protein 1 | CTD |
| 728 | SV2A | Q7L0J3 | Synaptic vesicle glycoprotein 2A | CTD |
| 729 | SV2C | Q496J9 | Synaptic vesicle glycoprotein 2C | CTD |
| 730 | TAC1 | P20366 | Protachykinin-1 | CTD |
| 731 | TACR3 | P29371 | Neuromedin-K receptor | CTD |
| 732 | TAZ | Q16635 | Tafazzin | CTD |
| 733 | TBC1D21 | Q8IYX1 | TBC1 domain family member 21 | CTD |
| 734 | TBL2 | Q9Y4P3 | Transducin beta-like protein 2 | CTD |
| 735 | TEK | Q02763 | TEK Receptor Tyrosine Kinase | GeneCard |
| 736 | TFB2M | Q9H5Q4 | Dimethyladenosine transferase 2, mitochondrial | CTD |
| 737 | TG | P01266 | Thyroglobulin | GeneCard/OMIM |
| 738 | TGFB1 | P01137 | Transforming Growth Factor Beta 1 | GeneCard/CTD |
| 739 | THBS1 | P07996 | Thrombospondin-1 | CTD |
| 740 | THBS3 | P49746 | Thrombospondin-3 | CTD |
| 741 | THRA | P10827 | Thyroid Hormone Receptor Alpha | GeneCard |
| 742 | THRB | P10828 | Thyroid Hormone Receptor Beta | GeneCard |
| 743 | THY1 | P04216 | Thy-1 Cell Surface Antigen | GeneCard |
| 744 | TIMM17B | O60830 | Mitochondrial import inner membrane translocase subunit Tim17-B | CTD |
| 745 | TIMM22 | Q9Y584 | Mitochondrial import inner membrane translocase subunit Tim22 | CTD |
| 746 | TIMM44 | O43615 | Mitochondrial import inner membrane translocase subunit TIM44 | CTD |
| 747 | TIMM8A | O60220 | Mitochondrial import inner membrane translocase subunit Tim8 A | CTD |
| 748 | TIMP1 | P01033 | TIMP Metallopeptidase Inhibitor 1 | GeneCard |
| 749 | TLR10 | Q9BXR5 | Toll Like Receptor 10 | GeneCard |
| 750 | TLR2 | O60603 | Toll-like receptor 2 | CTD |
| 751 | TLR4 | O00206 | Toll Like Receptor 4 | GeneCard |
| 752 | TLR9 | Q9NR96 | Toll Like Receptor 9 | GeneCard |
| 753 | TM6SF2 | Q9BZW4 | Transmembrane 6 superfamily member 2 | CTD |
| 754 | TMC4 | Q7Z404 | Transmembrane channel-like protein 4 | CTD |
| 755 | TMEM14C | Q9P0S9 | Transmembrane protein 14C | CTD |
| 756 | TMEM177 | Q53S58 | Transmembrane protein 177 | CTD |
| 757 | TMEM246 | Q9BRR3 | Transmembrane protein 246 | CTD |
| 758 | TMEM41A | Q96HV5 | Transmembrane protein 41A | CTD |
| 759 | TMEM86B | Q8N661 | Lysoplasmalogenase | CTD |
| 760 | TMOD1 | P28289 | Tropomodulin-1 | OMIM |
| 761 | TMPRSS6 | Q8IU80 | Transmembrane protease serine 6 | CTD |
| 762 | TNF | P01375 | Tumor Necrosis Factor | GeneCard/CTD |
| 763 | TNFAIP2 | Q03169 | Tumor necrosis factor alpha-induced protein 2 | CTD |
| 764 | TNFRSF12A | Q9NP84 | TNF Receptor Superfamily Member 12A | GeneCard |
| 765 | TNFSF12 | O43508 | TNF Superfamily Member 12 | GeneCard |
| 766 | TNFSF13B | Q9Y275 | TNF Superfamily Member 13b | GeneCard |
| 767 | TOMM70 | O94826 | Mitochondrial import receptor subunit TOM70 | CTD |
| 768 | TP53 | P04637 | Tumor Protein P53 | GeneCard |
| 769 | TPO | P07202 | Thyroid Peroxidase | GeneCard/OMIM |
| 770 | TPP1 | O14773 | Tripeptidyl Peptidase 1 | GeneCard |
| 771 | TRIM54 | Q9BYV2 | Tripartite motif-containing protein 54 | CTD |
| 772 | TRIM63 | Q969Q1 | E3 ubiquitin-protein ligase TRIM63 | CTD |
| 773 | TRPM6 | Q9BX84 | Transient receptor potential cation channel subfamily M member 6 | CTD |
| 774 | TSHB | P01222 | Thyroid Stimulating Hormone Subunit Beta | GeneCard |
| 775 | TSHR | P16473 | Thyroid Stimulating Hormone Receptor | GeneCard |
| 776 | TTN | Q8WZ42 | Titin | CTD |
| 777 | TUBB1 | Q9H4B7 | Tubulin beta-1 chain | CTD |
| 778 | TXNIP | Q9H3M7 | Thioredoxin-interacting protein | CTD |
| 779 | UACA | Q9BZF9 | Uveal Autoantigen With Coiled-Coil Domains And Ankyrin Repeats | GeneCard |
| 780 | UBA1 | P22314 | Ubiquitin-like modifier-activating enzyme 1 | OMIM |
| 781 | UBASH3A | P57075 | Ubiquitin Associated And SH3 Domain Containing A | GeneCard |
| 782 | UBE2G2 | P60604 | Ubiquitin-conjugating enzyme E2 G2 | CTD |
| 783 | UBE2L3 | P68036 | Ubiquitin Conjugating Enzyme E2 L3 | GeneCard |
| 784 | UCP1 | P25874 | Uncoupling Protein 1 | GeneCard |
| 785 | UGDH | O60701 | UDP-Glucose 6-Dehydrogenase | GeneCard |
| 786 | UQCR11 | O14957 | Cytochrome b-c1 complex subunit 10 | CTD |
| 787 | USH1C | Q9Y6N9 | Harmonin | CTD |
| 788 | USP43 | Q70EL4 | Ubiquitin carboxyl-terminal hydrolase 43 | CTD |
| 789 | VCAM1 | P19320 | Vascular Cell Adhesion Molecule 1 | GeneCard |
| 790 | VCAN | P13611 | Versican | GeneCard |
| 791 | VDR | P11473 | Vitamin D Receptor | GeneCard/OMIM/CTD |
| 792 | VEGFA | P15692 | Vascular Endothelial Growth Factor A | GeneCard/CTD |
| 793 | VPS37D | Q86XT2 | Vacuolar protein sorting-associated protein 37D | CTD |
| 794 | VWF | P04275 | Von Willebrand Factor | GeneCard/CTD |
| 795 | WDR7 | Q9Y4E6 | WD repeat-containing protein 7 | CTD |
| 796 | WDR72 | Q3MJ13 | WD repeat-containing protein 72 | CTD |
| 797 | WFS1 | O76024 | Wolframin | OMIM |
| 798 | WISP2 | O76076 | WNT1-inducible-signaling pathway protein 2 | CTD |
| 799 | WWC2 | Q6AWC2 | Protein WWC2 | CTD |
| 800 | XDH | P47989 | Xanthine Dehydrogenase | GeneCard |
| 801 | XIRP1 | Q702N8 | Xin actin-binding repeat-containing protein 1 | CTD |
| 802 | XRCC6 | P12956 | X-ray repair cross-complementing protein 6 | OMIM |
| 803 | YPEL4 | Q96NS1 | Protein yippee-like 4 | CTD |
| 804 | ZBTB7B | O15156 | Zinc finger and BTB domain-containing protein 7B | CTD |
| 805 | ZC3H11A | O75152 | Zinc finger CCCH domain-containing protein 11A | CTD |
| 806 | ZCWPW2 | Q504Y3 | Zinc finger CW-type PWWP domain protein 2 | CTD |
| 807 | ZDHHC15 | Q96MV8 | Palmitoyltransferase ZDHHC15 | CTD |
| 808 | ZFP36 | P26651 | ZFP36 Ring Finger Protein | GeneCard |
| 809 | ZFX | P17010 | Zinc finger X-chromosomal protein | OMIM |
| 810 | ZFY | P08048 | Zinc finger Y-chromosomal protein | OMIM |
| 811 | ZNF117 | Q03924 | Zinc finger protein 117 | CTD |
| 812 | ZNF681 | Q96N22 | Zinc finger protein 681 | CTD |
| 813 | ZSWIM2 | Q8NEG5 | E3 ubiquitin-protein ligase ZSWIM2 | CTD |
