# Supplementary material for: Mechanisms of Spica Prunellae against thyroid-associated Ophthalmopathy based on network pharmacology and molecular docking
Source: BMC Complement Med Ther. 2020 Jul 20;20:229. doi: 10.1186/s12906-020-03022-2 (PMC7372882; doi:10.1186/s12906-020-03022-2)
Supplement: Supplementary file 3 — Additional file 3: Table S3 Information for TAO targets after PPI analysis. [file 12906_2020_3022_MOESM3_ESM.docx]

**Table S3** Information for TAO targets after PPI analysis.

| **Number** | **Name** | **Protein name** | **Degree** | **Betweenness Centrality** | **Closeness Centrality** |
| --- | --- | --- | --- | --- | --- |
| 1 | IL6 | Interleukin-6 | 262 | 0.04091 | 0.55333 |
| 2 | INS | Insulin | 247 | 0.08593 | 0.56292 |
| 3 | TNF | Tumor Necrosis Factor | 242 | 0.02575 | 0.53857 |
| 4 | ALB | Serum albumin | 229 | 0.06610 | 0.55913 |
| 5 | AKT1 | RAC-alpha serine/threonine-protein kinase | 221 | 0.03718 | 0.54327 |
| 6 | TP53 | Tumor Protein P53 | 205 | 0.05116 | 0.53780 |
| 7 | CXCL8 | Interleukin-8 | 190 | 0.01333 | 0.50990 |
| 8 | IL10 | Interleukin-10 | 190 | 0.00963 | 0.51340 |
| 9 | STAT3 | Signal Transducer And Activator Of Transcription 3 | 190 | 0.01882 | 0.51376 |
| 10 | VEGFA | Vascular Endothelial Growth Factor A | 189 | 0.01428 | 0.51482 |
| 11 | MAPK3 | Mitogen-activated protein kinase 3 | 181 | 0.02384 | 0.51911 |
| 12 | IL1B | Interleukin-1 beta | 165 | 0.01082 | 0.49866 |
| 13 | EGF | Epidermal Growth Factor | 164 | 0.01492 | 0.49274 |
| 14 | IL4 | Interleukin-4 | 164 | 0.00623 | 0.48855 |
| 15 | TLR4 | Toll Like Receptor 4 | 163 | 0.00944 | 0.49767 |
| 16 | CCL2 | C-C Motif Chemokine Ligand 2 | 161 | 0.00574 | 0.49866 |
| 17 | FN1 | Fibronectin 1 | 160 | 0.02300 | 0.50033 |
| 18 | IL2 | Interleukin 2 | 159 | 0.00498 | 0.48664 |
| 19 | IFNG | Interferon gamma | 157 | 0.00586 | 0.48696 |
| 20 | CD44 | CD44 Molecule (Indian Blood Group) | 154 | 0.01261 | 0.48475 |
| 21 | PTPRC | Wolframin | 154 | 0.01071 | 0.48538 |
| 22 | JUN | Transcription factor AP-1 | 152 | 0.01119 | 0.50713 |
| 23 | MAPK1 | Mitogen-activated protein kinase 1 | 152 | 0.01962 | 0.50303 |
| 24 | ICAM1 | Intercellular Adhesion Molecule 1 | 151 | 0.00770 | 0.49372 |
| 25 | CASP3 | Caspase-3 | 150 | 0.01281 | 0.50541 |
| 26 | MMP9 | Matrix Metallopeptidase 9 | 147 | 0.00819 | 0.49437 |
| 27 | STAT1 | Signal transducer and activator of transcription 1-alpha/beta | 147 | 0.00842 | 0.47732 |
| 28 | CCL5 | C-C Motif Chemokine Ligand 5 | 145 | 0.00410 | 0.47946 |
| 29 | CXCL10 | C-X-C Motif Chemokine Ligand 10 | 145 | 0.00837 | 0.47946 |
| 30 | IGF1 | Insulin Like Growth Factor 1 | 144 | 0.00649 | 0.49470 |
| 31 | IL17A | Interleukin 17A | 144 | 0.00451 | 0.48069 |
| 32 | TLR2 | Toll-like receptor 2 | 143 | 0.00635 | 0.47854 |
| 33 | CXCR4 | C-X-C chemokine receptor type 4 | 138 | 0.01025 | 0.48444 |
| 34 | PTGS2 | Prostaglandin-Endoperoxide Synthase 2 | 135 | 0.01039 | 0.48855 |
| 35 | CD40 | CD40 Molecule | 132 | 0.00460 | 0.47219 |
| 36 | CD86 | CD86 Molecule | 131 | 0.00436 | 0.46426 |
| 37 | VCAM1 | Vascular Cell Adhesion Molecule 1 | 131 | 0.00376 | 0.47549 |
| 38 | FOXP3 | Forkhead Box P3 | 130 | 0.00295 | 0.47368 |
| 39 | LEP | Leptin | 126 | 0.00437 | 0.47549 |
| 40 | CTLA4 | Cytotoxic T-Lymphocyte Associated Protein 4 | 125 | 0.00344 | 0.46863 |
| 41 | IL13 | Interleukin-13 | 125 | 0.00174 | 0.46629 |
| 42 | NOTCH1 | Neurogenic locus notch homolog protein 1 | 124 | 0.00855 | 0.48760 |
| 43 | CCND1 | Cyclin D1 | 120 | 0.00493 | 0.48069 |
| 44 | CXCL12 | C-X-C Motif Chemokine Ligand 12 | 120 | 0.00284 | 0.47011 |
| 45 | IL18 | Interleukin 18 | 120 | 0.00129 | 0.46484 |
| 46 | PTEN | Phosphatase And Tensin Homolog | 118 | 0.01435 | 0.49372 |
| 47 | CXCL1 | Growth-regulated alpha protein | 117 | 0.00370 | 0.47429 |
| 48 | CD80 | CD80 Molecule | 113 | 0.00126 | 0.45493 |
| 49 | TLR9 | Toll Like Receptor 9 | 113 | 0.00117 | 0.46340 |
| 50 | IL5 | Interleukin-5 | 112 | 0.00142 | 0.46197 |
| 51 | ITGAX | Integrin Subunit Alpha X | 112 | 0.00245 | 0.45604 |
| 52 | CD40LG | CD40 Ligand | 111 | 0.00217 | 0.46197 |
| 53 | RELA | Transcription factor p65 | 109 | 0.01006 | 0.47580 |
| 54 | CSF3 | Colony Stimulating Factor 3 | 108 | 0.00097 | 0.46369 |
| 55 | ERBB2 | Erb-B2 Receptor Tyrosine Kinase 2 | 105 | 0.00539 | 0.47129 |
| 56 | PPARG | Peroxisome Proliferator Activated Receptor Gamma | 105 | 0.00368 | 0.47854 |
| 57 | JAK2 | Janus Kinase 2 | 104 | 0.00503 | 0.46600 |
| 58 | CD28 | CD28 Molecule | 103 | 0.00105 | 0.45466 |
| 59 | CXCL9 | C-X-C Motif Chemokine Ligand 9 | 103 | 0.00231 | 0.44811 |
| 60 | IL7 | Interleukin 7 | 100 | 0.00055 | 0.44973 |
| 61 | SELL | Selectin L | 100 | 0.00407 | 0.45000 |
| 62 | SOCS3 | Suppressor Of Cytokine Signaling 3 | 100 | 0.00730 | 0.46054 |
| 63 | SOD2 | Superoxide Dismutase 2 | 100 | 0.04759 | 0.48131 |
| 64 | NGF | Nerve Growth Factor | 97 | 0.00783 | 0.47762 |
| 65 | STAT6 | Signal transducer and activator of transcription 6 | 97 | 0.00115 | 0.45383 |
| 66 | TIMP1 | TIMP Metallopeptidase Inhibitor 1 | 97 | 0.00163 | 0.45744 |
| 67 | DDX41 | Probable ATP-dependent RNA helicase DDX41 | 95 | 0.00132 | 0.45604 |
| 68 | MPO | Myeloperoxidase | 95 | 0.00412 | 0.46863 |
| 69 | CXCR3 | C-X-C Motif Chemokine Receptor 3 | 94 | 0.00090 | 0.44071 |
| 70 | BCL2L1 | BCL2 Like 1 | 93 | 0.00271 | 0.47129 |
| 71 | SIRT1 | NAD-dependent protein deacetylase sirtuin-1 | 93 | 0.00939 | 0.47549 |
| 72 | IL2RA | Interleukin 2 Receptor Subunit Alpha | 92 | 0.00138 | 0.44438 |
| 73 | TGFB1 | Transforming Growth Factor Beta 1 | 92 | 0.00321 | 0.46225 |
| 74 | NFKB1 | Nuclear Factor Kappa B Subunit 1 | 91 | 0.00734 | 0.45828 |
| 75 | CAT | Catalase | 90 | 0.01137 | 0.47308 |
| 76 | EDN1 | Endothelin-1 | 90 | 0.00664 | 0.46484 |
| 77 | POMC | Proopiomelanocortin | 90 | 0.00487 | 0.45273 |
| 78 | TLR10 | Toll Like Receptor 10 | 90 | 0.00057 | 0.45549 |
| 79 | ADIPOQ | Adiponectin | 89 | 0.00607 | 0.46455 |
| 80 | IL3 | Interleukin 3 | 88 | 0.00180 | 0.45218 |
| 81 | IRF1 | Interferon regulatory factor 1 | 88 | 0.00196 | 0.43864 |
| 82 | CCL20 | C-C motif chemokine 20 | 87 | 0.00082 | 0.42710 |
| 83 | JAK1 | Tyrosine-protein kinase JAK1 | 86 | 0.00194 | 0.44946 |
| 84 | CASP8 | Caspase 8 | 85 | 0.00351 | 0.46340 |
| 85 | FASLG | Fas Ligand | 85 | 0.00505 | 0.45521 |
| 86 | CXCL2 | C-X-C motif chemokine 2 | 84 | 0.00094 | 0.44597 |
| 87 | HMOX1 | Heme oxygenase 1 | 84 | 0.00160 | 0.46369 |
| 88 | IFNB1 | Interferon beta | 84 | 0.00419 | 0.44123 |
| 89 | MMP3 | Matrix Metallopeptidase 3 | 83 | 0.00202 | 0.45081 |
| 90 | SELE | Selectin E | 83 | 0.00432 | 0.44838 |
| 91 | THY1 | Thy-1 Cell Surface Antigen | 83 | 0.01312 | 0.46717 |
| 92 | CD69 | CD69 Molecule | 82 | 0.00104 | 0.43659 |
| 93 | NOS2 | Nitric oxide synthase, inducible | 82 | 0.00321 | 0.46083 |
| 94 | SERPINE1 | Serpin Family E Member 1 | 82 | 0.00083 | 0.45300 |
| 95 | FOXO1 | Forkhead box protein O1 | 81 | 0.00263 | 0.46083 |
| 96 | HIF1A | Hypoxia-inducible factor 1-alpha | 81 | 0.00257 | 0.46600 |
| 97 | CCR1 | C-C Motif Chemokine Receptor 1 | 80 | 0.00093 | 0.43304 |
| 98 | IRF7 | Interferon regulatory factor 7 | 79 | 0.00192 | 0.43030 |
| 99 | FCGR2B | Low affinity immunoglobulin gamma Fc region receptor II-b | 78 | 0.00130 | 0.42882 |
| 100 | STAT4 | Signal transducer and activator of transcription 4 | 78 | 0.00319 | 0.43633 |
| 101 | CXCL11 | C-X-C Motif Chemokine Ligand 11 | 77 | 0.00096 | 0.42371 |
| 102 | IL1R1 | Interleukin 1 Receptor Type 1 | 76 | 0.00022 | 0.43941 |
| 103 | IL2RB | Interleukin 2 Receptor Subunit Beta | 76 | 0.00090 | 0.42857 |
| 104 | SDC1 | Syndecan 1 | 76 | 0.00364 | 0.44411 |
| 105 | AR | Androgen receptor | 75 | 0.00630 | 0.45744 |
| 106 | CCN2 | CCN family member 2 | 74 | 0.00328 | 0.45109 |
| 730 | IGF1R | Insulin-like growth factor 1 receptor | 74 | 0.00276 | 0.45913 |
| 107 | IL21 | Interleukin 21 | 73 | 0.00035 | 0.44280 |
| 108 | CCL11 | Eotaxin | 72 | 0.00029 | 0.43279 |
| 109 | FCGR2C | Low affinity immunoglobulin gamma Fc region receptor II-c | 72 | 0.00167 | 0.42203 |
| 110 | IGF2 | Insulin Like Growth Factor 2 | 72 | 0.00924 | 0.44704 |
| 111 | IL1A | Interleukin 1 Alpha | 72 | 0.00062 | 0.44919 |
| 731 | SOX9 | SRY-Box Transcription Factor 9 | 72 | 0.00677 | 0.46054 |
| 112 | GCG | Glucagon | 71 | 0.00580 | 0.44306 |
| 113 | TNFSF13B | TNF Superfamily Member 13b | 70 | 0.00048 | 0.42931 |
| 114 | BMP4 | Bone Morphogenetic Protein 4 | 69 | 0.00316 | 0.45109 |
| 115 | IL9 | Interleukin 9 | 68 | 0.00014 | 0.41271 |
| 116 | IRF5 | Interferon Regulatory Factor 5 | 68 | 0.00197 | 0.41943 |
| 117 | CDKN1A | Cyclin-dependent kinase inhibitor 1 | 67 | 0.00216 | 0.45273 |
| 118 | MAP2K1 | Dual specificity mitogen-activated protein kinase kinase 1 | 67 | 0.00168 | 0.45328 |
| 119 | GPT | Glutamic--Pyruvic Transaminase | 66 | 0.00719 | 0.45856 |
| 120 | ITGB2 | Integrin Subunit Beta 2 | 66 | 0.00296 | 0.42443 |
| 121 | NR3C1 | Glucocorticoid receptor | 66 | 0.00257 | 0.44973 |
| 122 | VWF | Von Willebrand Factor | 66 | 0.00703 | 0.44464 |
| 123 | ISG15 | Ubiquitin-like protein ISG15 | 65 | 0.01191 | 0.43967 |
| 124 | IFIH1 | Interferon-induced helicase C domain-containing protein 1 | 64 | 0.00128 | 0.41271 |
| 125 | SST | Somatostatin | 64 | 0.00372 | 0.43531 |
| 126 | THBS1 | Thrombospondin-1 | 64 | 0.00130 | 0.43967 |
| 127 | CD5 | CD5 Molecule | 63 | 0.00060 | 0.43129 |
| 128 | DDX58 | Probable ATP-dependent RNA helicase DDX58 | 63 | 0.00092 | 0.42227 |
| 129 | FAS | Fas Cell Surface Death Receptor | 62 | 0.00033 | 0.43229 |
| 130 | IL23R | Interleukin 23 Receptor | 62 | 0.00202 | 0.41134 |
| 131 | COL1A1 | Collagen alpha-1 | 61 | 0.00277 | 0.43967 |
| 132 | IL1RN | Interleukin-1 receptor antagonist protein | 61 | 0.00038 | 0.42857 |
| 133 | CD1D | Antigen-presenting glycoprotein CD1d | 60 | 0.00019 | 0.41616 |
| 134 | ITGAL | Integrin Subunit Alpha L | 60 | 0.00056 | 0.41966 |
| 135 | MX1 | Interferon-induced GTP-binding protein Mx1 | 60 | 0.00252 | 0.42661 |
| 136 | MRPS16 | 28S ribosomal protein S16, mitochondrial | 59 | 0.00476 | 0.36582 |
| 137 | PDGFRA | Platelet Derived Growth Factor Receptor Alpha | 59 | 0.00361 | 0.44784 |
| 138 | AGTR1 | Type-1 angiotensin II receptor | 58 | 0.00297 | 0.43787 |
| 139 | GNB3 | Guanine nucleotide-binding protein G | 58 | 0.00565 | 0.41454 |
| 140 | IL4R | Interleukin-4 receptor subunit alpha | 58 | 0.00015 | 0.41044 |
| 141 | ITGA4 | Integrin Subunit Alpha 4 | 58 | 0.00155 | 0.41202 |
| 142 | KLRK1 | Killer Cell Lectin Like Receptor K1 | 58 | 0.00021 | 0.41248 |
| 143 | SOD1 | Superoxide Dismutase 1 | 58 | 0.01005 | 0.45521 |
| 144 | MRPL2 | 39S ribosomal protein L2, mitochondrial | 57 | 0.00481 | 0.36907 |
| 145 | DICER1 | Endoribonuclease Dicer | 56 | 0.02003 | 0.45772 |
| 146 | EZH2 | Histone-lysine N-methyltransferase EZH2 | 56 | 0.00351 | 0.43582 |
| 147 | ITGAE | Integrin Subunit Alpha E | 56 | 0.00013 | 0.40357 |
| 148 | PDGFB | Platelet Derived Growth Factor Subunit B | 56 | 0.00164 | 0.43787 |
| 149 | HLA-A | Major Histocompatibility Complex, Class I, A | 55 | 0.00298 | 0.42735 |
| 150 | MRPS14 | 28S ribosomal protein S14, mitochondrial | 55 | 0.00379 | 0.36889 |
| 151 | TAC1 | Protachykinin-1 | 55 | 0.00686 | 0.43506 |
| 152 | MRPS5 | 28S ribosomal protein S5, mitochondrial | 54 | 0.00544 | 0.37481 |
| 153 | CXCL3 | C-X-C motif chemokine 3 | 53 | 0.00026 | 0.41044 |
| 154 | GFAP | Glial fibrillary acidic protein | 53 | 0.00676 | 0.45000 |
| 155 | HSPA1A | Heat Shock Protein Family A (Hsp70) Member 1A | 53 | 0.01110 | 0.45328 |
| 156 | IGFBP3 | Insulin Like Growth Factor Binding Protein 3 | 53 | 0.00058 | 0.43838 |
| 157 | IL23A | Interleukin-23 subunit alpha | 53 | 0.00011 | 0.40976 |
| 158 | MRPL15 | 39S ribosomal protein L15, mitochondrial | 53 | 0.00454 | 0.36708 |
| 159 | PPARGC1A | Peroxisome proliferator-activated receptor gamma coactivator 1-alpha | 53 | 0.00734 | 0.44973 |
| 160 | IL17F | Interleukin 17F | 52 | 0.00009 | 0.39798 |
| 161 | BGLAP | Osteocalcin | 51 | 0.00360 | 0.44149 |
| 162 | LTA | Lymphotoxin Alpha | 51 | 0.00034 | 0.40291 |
| 163 | S1PR1 | Sphingosine-1-Phosphate Receptor 1 | 50 | 0.00025 | 0.41943 |
| 164 | COL1A2 | Collagen Type I Alpha 2 Chain | 49 | 0.00134 | 0.42613 |
| 165 | MRPL24 | 39S ribosomal protein L24, mitochondrial | 49 | 0.00094 | 0.32046 |
| 166 | MRPL4 | 39S ribosomal protein L4, mitochondrial | 48 | 0.00081 | 0.35005 |
| 167 | MRPL44 | 39S ribosomal protein L44, mitochondrial | 48 | 0.00402 | 0.35690 |
| 168 | MRPS11 | 28S ribosomal protein S11, mitochondrial | 48 | 0.00099 | 0.34535 |
| 169 | MRPS15 | 28S ribosomal protein S15, mitochondrial | 48 | 0.00282 | 0.36492 |
| 170 | PTPN22 | Protein Tyrosine Phosphatase Non-Receptor Type 22 | 48 | 0.00665 | 0.41755 |
| 171 | ABCG2 | ATP-binding cassette sub-family G member 2 | 47 | 0.00849 | 0.44227 |
| 172 | HLA-DRB1 | Major Histocompatibility Complex, Class II, DR Beta 1 | 47 | 0.00090 | 0.42180 |
| 173 | IFNAR2 | Interferon alpha/beta receptor 2 | 47 | 0.00021 | 0.40400 |
| 174 | MRPL16 | 39S ribosomal protein L16, mitochondrial | 47 | 0.00173 | 0.35038 |
| 175 | MRPL55 | 39S ribosomal protein L55, mitochondrial | 47 | 0.00102 | 0.32663 |
| 176 | MRPS2 | 28S ribosomal protein S2, mitochondrial | 47 | 0.00218 | 0.35759 |
| 177 | MRPS34 | 28S ribosomal protein S34, mitochondrial | 47 | 0.00068 | 0.34282 |
| 178 | NFE2L2 | Nuclear factor erythroid 2-related factor 2 | 47 | 0.00122 | 0.44254 |
| 179 | OASL | 2'-5'-oligoadenylate synthase-like protein | 47 | 0.00538 | 0.39819 |
| 180 | PARP1 | Poly(ADP-Ribose) Polymerase 1 | 47 | 0.00575 | 0.44570 |
| 181 | SPARC | SPARC | 47 | 0.00332 | 0.43154 |
| 182 | VDR | Vitamin D Receptor | 47 | 0.00167 | 0.43812 |
| 183 | GBP1 | Guanylate-binding protein 1 | 46 | 0.00059 | 0.41385 |
| 184 | IL16 | Interleukin 16 | 46 | 0.00101 | 0.40313 |
| 185 | MRPL22 | 39S ribosomal protein L22, mitochondrial | 46 | 0.00063 | 0.34972 |
| 186 | MRPL34 | 39S ribosomal protein L34, mitochondrial | 46 | 0.00071 | 0.34988 |
| 187 | MRPL35 | 39S ribosomal protein L35, mitochondrial | 46 | 0.00028 | 0.31950 |
| 188 | MRPL43 | 39S ribosomal protein L43, mitochondrial | 46 | 0.00104 | 0.35605 |
| 189 | MRPL48 | 39S ribosomal protein L48, mitochondrial | 46 | 0.00032 | 0.32763 |
| 190 | MRPL53 | 39S ribosomal protein L53, mitochondrial | 46 | 0.00724 | 0.35319 |
| 191 | MRPL9 | 39S ribosomal protein L9, mitochondrial | 46 | 0.00026 | 0.31801 |
| 192 | MRPS18A | 39S ribosomal protein S18a, mitochondrial | 46 | 0.00190 | 0.35571 |
| 193 | OAS1 | 2'-5'-oligoadenylate synthase 1 | 46 | 0.00034 | 0.39947 |
| 194 | SLC2A1 | Solute carrier family 2, facilitated glucose transporter member 1 | 46 | 0.00185 | 0.44491 |
| 195 | AKT2 | AKT Serine/Threonine Kinase 2 | 45 | 0.00086 | 0.44280 |
| 196 | ATP5F1A | ATP synthase subunit alpha, mitochondrial | 45 | 0.00506 | 0.37613 |
| 197 | CALCA | Calcitonin gene-related peptide 1 | 45 | 0.00196 | 0.42516 |
| 198 | MRPL40 | 39S ribosomal protein L40, mitochondrial | 45 | 0.00100 | 0.35538 |
| 199 | MRPL45 | 39S ribosomal protein L45, mitochondrial | 45 | 0.00039 | 0.32720 |
| 200 | MRPL47 | 39S ribosomal protein L47, mitochondrial | 45 | 0.00281 | 0.35353 |
| 201 | MRPL57 | Ribosomal protein 63, mitochondrial | 45 | 0.00109 | 0.31801 |
| 732 | PSMB9 | Proteasome 20S Subunit Beta 9 | 45 | 0.00151 | 0.41616 |
| 202 | ADRB2 | Adrenoceptor Beta 2 | 44 | 0.00156 | 0.42833 |
| 203 | CDKN1B | Cyclin-dependent kinase inhibitor 1B | 44 | 0.00054 | 0.43254 |
| 204 | HLA-B | Major Histocompatibility Complex, Class I, B | 44 | 0.00263 | 0.40011 |
| 205 | MME | Membrane Metalloendopeptidase | 44 | 0.00144 | 0.43129 |
| 206 | MRPL30 | 39S ribosomal protein L30, mitochondrial | 44 | 0.00020 | 0.31666 |
| 207 | MRPL46 | 39S ribosomal protein L46, mitochondrial | 44 | 0.00092 | 0.35286 |
| 208 | MRPL49 | 39S ribosomal protein L49, mitochondrial | 44 | 0.01133 | 0.39357 |
| 209 | MRPL51 | 39S ribosomal protein L51, mitochondrial | 44 | 0.00111 | 0.31869 |
| 210 | MRPS28 | 28S ribosomal protein S28, mitochondrial | 44 | 0.00122 | 0.32706 |
| 211 | MRPS9 | 28S ribosomal protein S9, mitochondrial | 44 | 0.00090 | 0.35286 |
| 212 | ABCB1 | ATP-dependent translocase ABCB1 | 43 | 0.00375 | 0.43104 |
| 213 | ETS1 | Protein C-ets-1 | 43 | 0.00068 | 0.43229 |
| 214 | GNAS | GNAS Complex Locus | 43 | 0.00156 | 0.41662 |
| 215 | HAVCR2 | Hepatitis A Virus Cellular Receptor 2 | 43 | 0.00002 | 0.40118 |
| 216 | IFIT1 | Interferon-induced protein with tetratricopeptide repeats 1 | 43 | 0.00036 | 0.40140 |
| 217 | MBP | Myelin basic protein | 43 | 0.00047 | 0.42980 |
| 218 | MOG | Myelin-oligodendrocyte glycoprotein | 43 | 0.00339 | 0.41021 |
| 219 | MRPL14 | 39S ribosomal protein L14, mitochondrial | 43 | 0.00040 | 0.31492 |
| 220 | MRPL52 | 39S ribosomal protein L52, mitochondrial | 43 | 0.00011 | 0.31387 |
| 221 | MRPS26 | 28S ribosomal protein S26, mitochondrial | 43 | 0.00271 | 0.31321 |
| 222 | MRPS36 | 28S ribosomal protein S36, mitochondrial | 43 | 0.00045 | 0.31747 |
| 223 | PPARA | Peroxisome Proliferator Activated Receptor Alpha | 43 | 0.00152 | 0.43430 |
| 224 | VCAN | Versican | 43 | 0.00311 | 0.41546 |
| 225 | CYP19A1 | Aromatase | 42 | 0.00151 | 0.42956 |
| 226 | MRPL18 | 39S ribosomal protein L18, mitochondrial | 42 | 0.00011 | 0.31479 |
| 227 | MRPL37 | 39S ribosomal protein L37, mitochondrial | 42 | 0.00010 | 0.31308 |
| 228 | MRPS21 | 28S ribosomal protein S21, mitochondrial | 42 | 0.00010 | 0.31308 |
| 229 | MRPS23 | 28S ribosomal protein S23, mitochondrial | 42 | 0.00003 | 0.31281 |
| 230 | SDHB | Succinate Dehydrogenase Complex Iron Sulfur Subunit B | 42 | 0.02269 | 0.43104 |
| 231 | SERPINC1 | Antithrombin-III | 42 | 0.00245 | 0.41896 |
| 232 | TSHR | Thyroid Stimulating Hormone Receptor | 42 | 0.00455 | 0.41732 |
| 233 | CD1A | T-cell surface glycoprotein CD1a | 41 | 0.00011 | 0.40598 |
| 234 | CYP2E1 | Cytochrome P450 Family 2 Subfamily E Member 1 | 41 | 0.00215 | 0.43104 |
| 235 | HERC5 | E3 ISG15--protein ligase HERC5 | 41 | 0.00141 | 0.40248 |
| 236 | IGFBP1 | Insulin Like Growth Factor Binding Protein 1 | 41 | 0.00079 | 0.42395 |
| 237 | MRPS25 | 28S ribosomal protein S25, mitochondrial | 41 | 0.00000 | 0.31112 |
| 238 | MYOD1 | Myoblast determination protein 1 | 41 | 0.00893 | 0.43405 |
| 239 | CCL13 | C-C motif chemokine 13 | 40 | 0.00015 | 0.39840 |
| 240 | IL12B | Interleukin-12 subunit beta | 40 | 0.00005 | 0.40422 |
| 733 | MRPS17 | 28S ribosomal protein S17, mitochondrial | 40 | 0.00000 | 0.31099 |
| 241 | ACKR3 | Atypical chemokine receptor 3 | 39 | 0.00189 | 0.42180 |
| 242 | CCN1 | Cellular Communication Network Factor 1 | 39 | 0.00079 | 0.42980 |
| 243 | IL10RB | Interleukin-10 receptor subunit beta | 39 | 0.00006 | 0.39461 |
| 244 | LAMB1 | Laminin subunit beta-1 | 39 | 0.00138 | 0.41616 |
| 245 | PTGER4 | Prostaglandin E2 receptor EP4 subtype | 39 | 0.00262 | 0.41779 |
| 246 | TEK | TEK Receptor Tyrosine Kinase | 39 | 0.00007 | 0.43531 |
| 734 | TG | Thyroglobulin | 39 | 0.00465 | 0.43380 |
| 247 | ALPL | Alkaline phosphatase, tissue-nonspecific isozyme | 38 | 0.00506 | 0.43129 |
| 248 | ATP5F1C | ATP synthase subunit gamma, mitochondrial | 38 | 0.00205 | 0.35896 |
| 249 | BST2 | Bone marrow stromal antigen 2 | 38 | 0.00031 | 0.38347 |
| 250 | CD58 | CD58 Molecule | 38 | 0.00028 | 0.39069 |
| 251 | GSTP1 | Glutathione S-Transferase Pi 1 | 38 | 0.00102 | 0.42784 |
| 252 | HLA-DQA1 | Major Histocompatibility Complex, Class II, DQ Alpha 1 | 38 | 0.00052 | 0.41685 |
| 253 | SSTR2 | Somatostatin Receptor 2 | 38 | 0.00117 | 0.40731 |
| 254 | SSTR3 | Somatostatin Receptor 3 | 38 | 0.00287 | 0.41157 |
| 735 | UCP1 | Uncoupling Protein 1 | 38 | 0.00056 | 0.43104 |
| 255 | A2M | Alpha-2-macroglobulin | 37 | 0.00053 | 0.41709 |
| 256 | ARRB1 | Arrestin Beta 1 | 37 | 0.00295 | 0.43761 |
| 257 | ATP5F1D | ATP synthase subunit delta, mitochondrial | 37 | 0.00296 | 0.35087 |
| 258 | ATP5PO | ATP synthase subunit O, mitochondrial | 37 | 0.00342 | 0.36744 |
| 259 | CYP3A4 | Cytochrome P450 3A4 | 37 | 0.00411 | 0.41779 |
| 260 | HLA-DQB1 | Major Histocompatibility Complex, Class II, DQ Beta 1 | 37 | 0.00024 | 0.41112 |
| 261 | OAS3 | 2'-5'-oligoadenylate synthase 3 | 37 | 0.00016 | 0.37996 |
| 736 | RB1 | Retinoblastoma-associated protein | 37 | 0.00099 | 0.41943 |
| 737 | RSAD2 | Radical S-adenosyl methionine domain-containing protein 2 | 37 | 0.00023 | 0.38685 |
| 262 | BCL2A1 | Bcl-2-related protein A1 | 36 | 0.00021 | 0.41849 |
| 263 | ESR2 | Estrogen Receptor 2 | 36 | 0.00235 | 0.43710 |
| 264 | GPR183 | G-protein coupled receptor 183 | 36 | 0.00026 | 0.38249 |
| 265 | HERC6 | Probable E3 ubiquitin-protein ligase HERC6 | 36 | 0.00075 | 0.37090 |
| 266 | HSPB2 | Heat Shock Protein Family B (Small) Member 2 | 36 | 0.00124 | 0.43993 |
| 267 | NDUFB7 | NADH dehydrogenase [ubiquinone] 1 beta subcomplex subunit 7 | 36 | 0.00419 | 0.33878 |
| 268 | SSTR1 | Somatostatin Receptor 1 | 36 | 0.00033 | 0.40357 |
| 269 | CYP1A1 | Cytochrome P450 Family 1 Subfamily A Member 1 | 35 | 0.00336 | 0.41021 |
| 270 | CYP2B6 | Cytochrome P450 2B6 | 35 | 0.00154 | 0.40932 |
| 271 | GSR | Glutathione-Disulfide Reductase | 35 | 0.00221 | 0.43890 |
| 272 | IFIT3 | Interferon-induced protein with tetratricopeptide repeats 3 | 35 | 0.00029 | 0.39545 |
| 273 | ISG20 | Interferon-stimulated gene 20 kDa protein | 35 | 0.00011 | 0.37670 |
| 274 | LPL | Lipoprotein Lipase | 35 | 0.00269 | 0.41872 |
| 275 | NDUFB10 | NADH dehydrogenase [ubiquinone] 1 beta subcomplex subunit 10 | 35 | 0.00080 | 0.33259 |
| 276 | SSTR4 | Somatostatin Receptor 4 | 35 | 0.00041 | 0.40118 |
| 277 | SSTR5 | Somatostatin Receptor 5 | 35 | 0.00009 | 0.40140 |
| 278 | CD52 | CD52 Molecule | 34 | 0.00077 | 0.38485 |
| 279 | FBN1 | Fibrillin 1 | 34 | 0.00080 | 0.40753 |
| 280 | GBA | Zinc finger X-chromosomal protein | 34 | 0.00278 | 0.42323 |
| 281 | GH1 | Growth Hormone 1 | 34 | 0.00040 | 0.41662 |
| 282 | GNAT3 | Guanine nucleotide-binding protein G | 34 | 0.00043 | 0.40532 |
| 283 | HLA-DRA | HLA class II histocompatibility antigen, DR alpha chain | 34 | 0.00149 | 0.40664 |
| 284 | IL12A | Interleukin-12 subunit alpha | 34 | 0.00002 | 0.39233 |
| 285 | IL22RA1 | Interleukin-22 receptor subunit alpha-1 | 34 | 0.00004 | 0.38765 |
| 286 | NKX2-1 | NK2 Homeobox 1 | 34 | 0.00165 | 0.40999 |
| 287 | COL4A1 | Collagen alpha-1(IV) chain | 33 | 0.00067 | 0.39295 |
| 288 | CP | Ceruloplasmin | 33 | 0.00217 | 0.42180 |
| 289 | FBXO32 | F-box only protein 32 | 33 | 0.00364 | 0.41569 |
| 290 | SDHA | Succinate Dehydrogenase Complex Flavoprotein Subunit A | 33 | 0.00492 | 0.38308 |
| 291 | ATP5ME | ATP synthase subunit e, mitochondrial, ATPase subunit e | 32 | 0.00068 | 0.33259 |
| 292 | ATP5MG | ATP synthase subunit g, mitochondrial, ATPase subunit g | 32 | 0.00178 | 0.35269 |
| 293 | CLTC | Clathrin Heavy Chain | 32 | 0.00601 | 0.41454 |
| 294 | NAMPT | Nicotinamide Phosphoribosyltransferase | 32 | 0.00015 | 0.42857 |
| 738 | PLAT | Tissue-type plasminogen activator | 32 | 0.00094 | 0.42395 |
| 739 | TRIM63 | E3 ubiquitin-protein ligase TRIM63 | 32 | 0.00292 | 0.41546 |
| 295 | MMP12 | Macrophage metalloelastase | 31 | 0.00028 | 0.41616 |
| 296 | ATP5MF | ATP synthase subunit f, mitochondrial | 30 | 0.00083 | 0.33483 |
| 297 | CD79A | CD79a Molecule | 30 | 0.00179 | 0.40510 |
| 298 | ICAM3 | Intercellular adhesion molecule 3 | 30 | 0.00001 | 0.38846 |
| 299 | IFI27 | Interferon alpha-inducible protein 27, mitochondrial | 30 | 0.00045 | 0.36672 |
| 300 | MAPT | Microtubule-associated protein tau | 30 | 0.00533 | 0.42735 |
| 301 | NDUFB9 | NADH dehydrogenase [ubiquinone] 1 beta subcomplex subunit 9 | 30 | 0.00673 | 0.40183 |
| 302 | PTX3 | Pentraxin 3 | 30 | 0.00037 | 0.40999 |
| 740 | SIRT6 | NAD-dependent protein deacetylase sirtuin-6 | 30 | 0.00140 | 0.42564 |
| 303 | ATP5PF | ATP synthase-coupling factor 6, mitochondrial | 29 | 0.00344 | 0.37500 |
| 304 | FGF8 | Fibroblast growth factor 8 | 29 | 0.00086 | 0.42443 |
| 741 | IFI35 | Interferon-induced 35 kDa protein | 29 | 0.00019 | 0.36280 |
| 305 | ATP5PB | ATP synthase F | 28 | 0.00024 | 0.33230 |
| 306 | FABP4 | Fatty acid-binding protein, adipocyte | 28 | 0.00018 | 0.41872 |
| 307 | GPX1 | Glutathione peroxidase 1 | 28 | 0.00152 | 0.42759 |
| 308 | IFI6 | Interferon alpha-inducible protein 6 | 28 | 0.00002 | 0.36157 |
| 309 | LAMB2 | Laminin subunit beta-2 | 28 | 0.00324 | 0.41546 |
| 742 | NDUFA6 | NADH dehydrogenase [ubiquinone] 1 alpha subcomplex subunit 6 | 28 | 0.00200 | 0.34615 |
| 310 | GPR18 | N-arachidonyl glycine receptor | 27 | 0.00021 | 0.37146 |
| 311 | MMP10 | Stromelysin-2 | 27 | 0.00001 | 0.41779 |
| 312 | MUC5AC | Mucin-5AC | 27 | 0.00240 | 0.41225 |
| 313 | NDUFA11 | NADH dehydrogenase [ubiquinone] 1 alpha subcomplex subunit 11 | 27 | 0.00027 | 0.33009 |
| 314 | UQCR11 | Cytochrome b-c1 complex subunit 10 | 27 | 0.00028 | 0.33038 |
| 315 | BAX | Apoptosis regulator BAX | 26 | 0.00054 | 0.41802 |
| 316 | HMGA2 | High Mobility Group AT-Hook 2 | 26 | 0.00014 | 0.42833 |
| 317 | IFI44 | Interferon-induced protein 44 | 26 | 0.00001 | 0.35862 |
| 318 | LGALS9 | Galectin 9 | 26 | 0.00009 | 0.38846 |
| 319 | LY6E | Lymphocyte antigen 6E | 26 | 0.00117 | 0.36070 |
| 320 | NDUFB4 | NADH dehydrogenase [ubiquinone] 1 beta subcomplex subunit 4 | 26 | 0.00060 | 0.33649 |
| 321 | PLA2G4A | Cytosolic phospholipase A2 | 26 | 0.00448 | 0.40291 |
| 322 | PTS | 6-Pyruvoyltetrahydropterin Synthase | 26 | 0.00284 | 0.42013 |
| 323 | TPO | Thyroid Peroxidase | 26 | 0.00265 | 0.41225 |
| 324 | UBA1 | Ubiquitin-like modifier-activating enzyme 1 | 26 | 0.00275 | 0.39336 |
| 325 | ATP5F1E | ATP synthase subunit epsilon, mitochondrial, ATPase subunit epsilon | 25 | 0.00039 | 0.32937 |
| 326 | COL4A5 | Collagen Type IV Alpha 5 Chain | 25 | 0.00035 | 0.37481 |
| 327 | CYP2C9 | Cytochrome P450 2C9 | 25 | 0.00051 | 0.39336 |
| 328 | GAD2 | Glutamate Decarboxylase 2 | 25 | 0.00281 | 0.41021 |
| 329 | GSTM1 | Glutathione S-transferase Mu 1 | 25 | 0.00036 | 0.39650 |
| 330 | IFI44L | Interferon-induced protein 44-like | 25 | 0.00001 | 0.35845 |
| 331 | LRP2 | LDL Receptor Related Protein 2 | 25 | 0.00086 | 0.40708 |
| 332 | NOD1 | Nucleotide-binding oligomerization domain-containing protein 1 | 25 | 0.00002 | 0.39608 |
| 333 | PDGFA | Platelet Derived Growth Factor Subunit A | 25 | 0.00008 | 0.40598 |
| 334 | SNAP25 | Synaptosomal-associated protein 25 | 25 | 0.00719 | 0.40731 |
| 743 | SNCA | Alpha-synuclein | 25 | 0.00289 | 0.41685 |
| 335 | DEFB4A | Defensin Beta 4A | 24 | 0.00006 | 0.37996 |
| 336 | DMD | Dystrophin | 24 | 0.00726 | 0.41225 |
| 337 | DTX3L | E3 ubiquitin-protein ligase DTX3L | 24 | 0.00088 | 0.37350 |
| 338 | MATN3 | Matrilin 3 | 24 | 0.00147 | 0.39357 |
| 339 | MC4R | Melanocortin receptor 4 | 24 | 0.00110 | 0.39295 |
| 340 | MT-ATP6 | ATP synthase subunit a | 24 | 0.00064 | 0.34825 |
| 341 | NDUFA10 | NADH dehydrogenase [ubiquinone] 1 alpha subcomplex subunit 10, mitochondrial | 24 | 0.00016 | 0.32893 |
| 342 | NDUFC1 | NADH dehydrogenase [ubiquinone] 1 subunit C1, mitochondrial | 24 | 0.00021 | 0.32507 |
| 343 | RARA | Retinoic Acid Receptor Alpha | 24 | 0.00128 | 0.40864 |
| 344 | TTN | Titin | 24 | 0.00724 | 0.38308 |
| 345 | WFS1 | Wolframin | 24 | 0.00203 | 0.40909 |
| 346 | ANAPC10 | Anaphase-promoting complex subunit 10 | 23 | 0.00296 | 0.38846 |
| 347 | COL11A1 | Collagen alpha-1 | 23 | 0.00022 | 0.36798 |
| 348 | EDNRA | Endothelin Receptor Type A | 23 | 0.00088 | 0.40444 |
| 349 | EDNRB | Endothelin receptor type B | 23 | 0.00312 | 0.41112 |
| 350 | IAPP | Islet amyloid polypeptide | 23 | 0.00025 | 0.40510 |
| 351 | LYZ | Lysozyme | 23 | 0.00065 | 0.40248 |
| 352 | MTHFR | Methylenetetrahydrofolate Reductase | 23 | 0.00108 | 0.42132 |
| 353 | TSHB | Thyroid Stimulating Hormone Subunit Beta | 23 | 0.00040 | 0.35588 |
| 354 | AIRE | Autoimmune Regulator | 22 | 0.00104 | 0.39629 |
| 355 | CYP3A5 | Cytochrome P450 3A5 | 22 | 0.00034 | 0.37575 |
| 356 | LAMA3 | Laminin subunit alpha-3 | 22 | 0.00264 | 0.39028 |
| 357 | NDUFB11 | NADH dehydrogenase [ubiquinone] 1 beta subcomplex subunit 11, mitochondrial | 22 | 0.00038 | 0.34094 |
| 358 | PAX8 | Paired Box 8 | 22 | 0.00099 | 0.38846 |
| 359 | RIPK2 | Receptor-interacting serine/threonine-protein kinase 2 | 22 | 0.00069 | 0.38426 |
| 360 | SPP2 | Secreted phosphoprotein 24 | 22 | 0.00089 | 0.39336 |
| 361 | TIMM44 | Mitochondrial import inner membrane translocase subunit TIM44 | 22 | 0.00219 | 0.36070 |
| 362 | TNFSF12 | TNF Superfamily Member 12 | 22 | 0.00007 | 0.39883 |
| 363 | APC | APC Regulator Of WNT Signaling Pathway | 21 | 0.00231 | 0.40357 |
| 364 | CAPN2 | Calpain-2 catalytic subunit | 21 | 0.00056 | 0.40909 |
| 365 | F8 | Coagulation factor VIII | 21 | 0.00066 | 0.39734 |
| 366 | FCGR3B | Low affinity immunoglobulin gamma Fc region receptor III-B | 21 | 0.00041 | 0.37387 |
| 367 | NOTCH2 | Notch Receptor 2 | 21 | 0.00016 | 0.40097 |
| 368 | XRCC6 | X-ray repair cross-complementing protein 6 | 21 | 0.00696 | 0.40932 |
| 369 | ADORA2A | Adenosine receptor A2a | 20 | 0.00030 | 0.41477 |
| 370 | BCS1L | Mitochondrial chaperone BCS1 | 20 | 0.00316 | 0.37670 |
| 371 | BRD4 | Bromodomain Containing 4 | 20 | 0.00010 | 0.41523 |
| 372 | CD300A | CMRF35-like molecule 8 | 20 | 0.00052 | 0.36070 |
| 373 | CYP1B1 | Cytochrome P450 1B1 | 20 | 0.00031 | 0.40642 |
| 374 | CYP2C8 | Cytochrome P450 2C8 | 20 | 0.00018 | 0.36052 |
| 375 | HNF1A | Hepatocyte nuclear factor 1-alpha | 20 | 0.00241 | 0.41408 |
| 376 | LILRB1 | Leukocyte Immunoglobulin Like Receptor B1 | 20 | 0.00010 | 0.39049 |
| 377 | LILRB4 | Leukocyte Immunoglobulin Like Receptor B4 | 20 | 0.00007 | 0.36421 |
| 378 | NDUFAF2 | NADH dehydrogenase [ubiquinone] 1 alpha subcomplex assembly factor 2 | 20 | 0.00076 | 0.31612 |
| 379 | RNASE3 | Eosinophil cationic protein | 20 | 0.00211 | 0.39904 |
| 380 | SCD | Stearoyl-CoA Desaturase | 20 | 0.00471 | 0.40598 |
| 381 | TXNIP | Thioredoxin-interacting protein | 20 | 0.00016 | 0.41385 |
| 382 | AGRP | Agouti-related protein | 19 | 0.00010 | 0.39587 |
| 383 | BCL2 | Apoptosis regulator Bcl-2 | 19 | 0.00006 | 0.39629 |
| 384 | BMP6 | Bone Morphogenetic Protein 6 | 19 | 0.00012 | 0.40532 |
| 385 | COL5A2 | Collagen Type V Alpha 2 Chain | 19 | 0.00010 | 0.36035 |
| 386 | CYP2A6 | Cytochrome P450 2A6 | 19 | 0.00041 | 0.33259 |
| 387 | CYP2C19 | Cytochrome P450 2C19 | 19 | 0.00013 | 0.37257 |
| 388 | DIO2 | Type II iodothyronine deiodinase | 19 | 0.00042 | 0.38327 |
| 389 | DMP1 | Dentin matrix acidic phosphoprotein 1 | 19 | 0.00002 | 0.39461 |
| 390 | NCOA6 | Nuclear receptor coactivator 6 | 19 | 0.00053 | 0.38665 |
| 391 | NR5A1 | Nuclear Receptor Subfamily 5 Group A Member 1 | 19 | 0.00039 | 0.39545 |
| 392 | PDCD4 | Programmed Cell Death 4 | 19 | 0.00003 | 0.40248 |
| 393 | PTGER2 | Prostaglandin E Receptor 2 | 19 | 0.00014 | 0.38967 |
| 394 | COL11A2 | Collagen Type XI Alpha 2 Chain | 18 | 0.00039 | 0.35403 |
| 395 | DLK1 | Delta Like Non-Canonical Notch Ligand 1 | 18 | 0.00010 | 0.40797 |
| 396 | GC | GC Vitamin D Binding Protein | 18 | 0.00116 | 0.38465 |
| 397 | MID1 | E3 ubiquitin-protein ligase Midline-1 | 18 | 0.00004 | 0.37785 |
| 398 | MKRN1 | E3 ubiquitin-protein ligase makorin-1 | 18 | 0.00100 | 0.38465 |
| 399 | NPHS1 | Nephrin | 18 | 0.00124 | 0.41271 |
| 400 | RNF19B | E3 ubiquitin-protein ligase RNF19B | 18 | 0.00030 | 0.36762 |
| 401 | CDKN2B | Cyclin-dependent kinase 4 inhibitor B | 17 | 0.00004 | 0.38625 |
| 402 | CYP2D6 | Cytochrome P450 Family 2 Subfamily D Member 6 | 17 | 0.00022 | 0.37072 |
| 403 | FCER2 | Low affinity immunoglobulin epsilon Fc receptor | 17 | 0.00004 | 0.37331 |
| 404 | HSD11B1 | Corticosteroid 11-beta-dehydrogenase isozyme 1 | 17 | 0.00039 | 0.38947 |
| 405 | IGFBP2 | Insulin Like Growth Factor Binding Protein 2 | 17 | 0.00001 | 0.41089 |
| 406 | PLA2G6 | Phospholipase A2 Group VI | 17 | 0.00142 | 0.37481 |
| 407 | RXFP2 | Relaxin receptor 2 | 17 | 0.00016 | 0.38446 |
| 408 | SPTAN1 | Spectrin Alpha, Non-Erythrocytic 1 | 17 | 0.00345 | 0.39336 |
| 409 | UBE2L3 | Ubiquitin Conjugating Enzyme E2 L3 | 17 | 0.00070 | 0.38685 |
| 410 | AVPR1A | Vasopressin V1a receptor | 16 | 0.00015 | 0.36925 |
| 411 | COL9A1 | Collagen Type IX Alpha 1 Chain | 16 | 0.00005 | 0.35054 |
| 412 | COL9A3 | Collagen Type IX Alpha 3 Chain | 16 | 0.00007 | 0.35252 |
| 413 | CYP3A7 | Cytochrome P450 3A7 | 16 | 0.00017 | 0.37164 |
| 414 | FBXO22 | F-box only protein 22 | 16 | 0.00135 | 0.38035 |
| 415 | NMB | Neuromedin-B | 16 | 0.00083 | 0.36834 |
| 416 | SFRP1 | Secreted Frizzled Related Protein 1 | 16 | 0.00009 | 0.39336 |
| 417 | ZFP36 | ZFP36 Ring Finger Protein | 16 | 0.00002 | 0.40820 |
| 418 | ATP6V1E1 | V-type proton ATPase subunit E 1 | 15 | 0.00386 | 0.38446 |
| 419 | ATRX | Transcriptional regulator ATRX | 15 | 0.00413 | 0.37294 |
| 420 | BAK1 | BCL2 Antagonist/Killer 1 | 15 | 0.00409 | 0.39692 |
| 421 | COX7A2L | Cytochrome c oxidase subunit 7A-related protein, mitochondrial | 15 | 0.00002 | 0.30703 |
| 422 | CYP27B1 | Cytochrome P450 Family 27 Subfamily B Member 1 | 15 | 0.00051 | 0.40054 |
| 423 | DNAJA3 | DnaJ homolog subfamily A member 3, mitochondrial | 15 | 0.00279 | 0.40488 |
| 424 | E2F4 | Transcription factor E2F4 | 15 | 0.00004 | 0.38229 |
| 425 | FBXO15 | F-box only protein 15 | 15 | 0.00016 | 0.34345 |
| 426 | HAS2 | Hyaluronan Synthase 2 | 15 | 0.00028 | 0.38485 |
| 427 | IL17D | Interleukin-17D | 15 | 0.00001 | 0.37500 |
| 428 | KRT20 | Keratin, type I cytoskeletal 20 | 15 | 0.00294 | 0.38605 |
| 429 | MS4A1 | Membrane Spanning 4-Domains A1 | 15 | 0.00006 | 0.34988 |
| 430 | NEB | Nebulin | 15 | 0.00161 | 0.35369 |
| 431 | SERPINA4 | Kallistatin | 15 | 0.00371 | 0.40510 |
| 432 | SLC26A4 | Pendrin | 15 | 0.00269 | 0.35639 |
| 433 | SOX3 | Transcription factor SOX-3 | 15 | 0.00335 | 0.35571 |
| 434 | SPSB2 | SPRY domain-containing SOCS box protein 2 | 15 | 0.00006 | 0.34503 |
| 435 | THRB | Thyroid Hormone Receptor Beta | 15 | 0.00021 | 0.38565 |
| 436 | TNFRSF12A | TNF Receptor Superfamily Member 12A | 15 | 0.00002 | 0.37350 |
| 437 | CALCB | Calcitonin gene-related peptide 2 | 14 | 0.00001 | 0.34032 |
| 438 | CHL1 | Neural cell adhesion molecule L1-like protein | 14 | 0.00008 | 0.39629 |
| 439 | DDIT4 | DNA damage-inducible transcript 4 protein | 14 | 0.00002 | 0.39357 |
| 440 | NDN | Necdin | 14 | 0.00332 | 0.39192 |
| 441 | NID2 | Nidogen-2 | 14 | 0.00009 | 0.35302 |
| 442 | PDHA1 | Pyruvate dehydrogenase E1 component subunit alpha, somatic form, mitochondrial | 14 | 0.00135 | 0.34631 |
| 443 | PTPN12 | Protein Tyrosine Phosphatase Non-Receptor Type 12 | 14 | 0.00034 | 0.38347 |
| 444 | SERPINA7 | Serpin Family A Member 7 | 14 | 0.00040 | 0.37035 |
| 445 | SLCO1A2 | Solute carrier organic anion transporter family member 1A2 | 14 | 0.00781 | 0.36636 |
| 446 | SURF1 | Surfeit locus protein 1 | 14 | 0.00027 | 0.31653 |
| 447 | TACR3 | Neuromedin-K receptor | 14 | 0.00009 | 0.34188 |
| 448 | AMHR2 | Anti-Muellerian hormone type-2 receptor | 13 | 0.00061 | 0.37689 |
| 449 | CD300LF | CMRF35-like molecule 1 | 13 | 0.00005 | 0.35420 |
| 450 | EDN2 | Endothelin-2 | 13 | 0.00002 | 0.34392 |
| 451 | IL18RAP | Interleukin-18 receptor accessory protein | 13 | 0.00155 | 0.36192 |
| 452 | IMMT | MICOS complex subunit MIC60 | 13 | 0.00135 | 0.33955 |
| 453 | LAMB3 | Laminin subunit beta-3 | 13 | 0.00084 | 0.35504 |
| 454 | PLA2G12B | Group XIIB secretory phospholipase A2-like protein | 13 | 0.00046 | 0.36174 |
| 455 | POLA1 | DNA polymerase alpha catalytic subunit | 13 | 0.00161 | 0.36600 |
| 456 | QRFPR | Pyroglutamylated RF-amide peptide receptor | 13 | 0.00015 | 0.34392 |
| 457 | SLC25A16 | Solute Carrier Family 25 Member 16 | 13 | 0.00156 | 0.39566 |
| 458 | SLC2A14 | Solute carrier family 2, facilitated glucose transporter member 14 | 13 | 0.00028 | 0.39861 |
| 459 | SLC5A5 | Complement C1q tumor necrosis factor-related protein 5 | 13 | 0.00009 | 0.34047 |
| 460 | TFB2M | Dimethyladenosine transferase 2, mitochondrial | 13 | 0.00219 | 0.33053 |
| 461 | UBE2G2 | Ubiquitin-conjugating enzyme E2 G2 | 13 | 0.00000 | 0.33649 |
| 462 | XDH | Xanthine Dehydrogenase | 13 | 0.00081 | 0.40075 |
| 463 | AOX1 | Aldehyde oxidase | 12 | 0.00017 | 0.33955 |
| 464 | ASNS | Asparagine synthetase [glutamine-hydrolyzing] | 12 | 0.00187 | 0.37938 |
| 465 | BANK1 | B Cell Scaffold Protein With Ankyrin Repeats 1 | 12 | 0.00061 | 0.34744 |
| 466 | BTG2 | BTG Anti-Proliferation Factor 2 | 12 | 0.00003 | 0.40842 |
| 467 | CACNA1A | Transcription factor SOX-9 | 12 | 0.00459 | 0.38054 |
| 468 | CSNK1D | Casein kinase I isoform delta | 12 | 0.00298 | 0.37823 |
| 469 | CSRP3 | Cysteine and glycine-rich protein 3 | 12 | 0.00341 | 0.33259 |
| 470 | FBLN1 | Fibulin-1 | 12 | 0.00124 | 0.35913 |
| 471 | KCNJ2 | Potassium Inwardly Rectifying Channel Subfamily J Member 2 | 12 | 0.00486 | 0.35554 |
| 472 | MYH2 | Myosin-2 | 12 | 0.00026 | 0.38947 |
| 473 | RAB1B | Ras-related protein Rab-1B | 12 | 0.00627 | 0.37275 |
| 474 | STS | Steryl-sulfatase | 12 | 0.00043 | 0.36070 |
| 475 | THRA | Thyroid Hormone Receptor Alpha | 12 | 0.00043 | 0.38249 |
| 476 | TIMM17B | Mitochondrial import inner membrane translocase subunit Tim17-B | 12 | 0.00362 | 0.36510 |
| 477 | TIMM8A | Mitochondrial import inner membrane translocase subunit Tim8 A | 12 | 0.00036 | 0.32324 |
| 478 | TMPRSS6 | Transmembrane protease serine 6 | 12 | 0.00035 | 0.37127 |
| 479 | CACNA1S | Calcium Voltage-Gated Channel Subunit Alpha1 S | 11 | 0.00314 | 0.32366 |
| 480 | CCNT1 | Cyclin-T1 | 11 | 0.00023 | 0.37238 |
| 481 | COL13A1 | Collagen Type XIII Alpha 1 Chain | 11 | 0.00001 | 0.33801 |
| 482 | ECI2 | Enoyl-CoA delta isomerase 2, mitochondrial, EC 5.3.3.8 | 11 | 0.00484 | 0.35862 |
| 483 | EGLN2 | Egl nine homolog 2 | 11 | 0.00026 | 0.37613 |
| 484 | HTR3A | 5-hydroxytryptamine receptor 3A | 11 | 0.00277 | 0.34890 |
| 485 | IYD | Iodotyrosine deiodinase 1 | 11 | 0.00012 | 0.33097 |
| 486 | LILRA1 | Leukocyte immunoglobulin-like receptor subfamily A member 1 | 11 | 0.00004 | 0.34141 |
| 487 | NEU1 | Neuraminidase 1 | 11 | 0.00037 | 0.34988 |
| 744 | TOMM70 | Mitochondrial import receptor subunit TOM70 | 11 | 0.00049 | 0.33862 |
| 488 | CAD | Zinc finger Y-chromosomal protein | 10 | 0.00242 | 0.37519 |
| 489 | CD2AP | CD2-associated protein | 10 | 0.00020 | 0.38054 |
| 490 | COQ9 | Ubiquinone biosynthesis protein COQ9, mitochondrial | 10 | 0.00005 | 0.32074 |
| 491 | DMRT1 | Doublesex- and mab-3-related transcription factor 1 | 10 | 0.00021 | 0.34032 |
| 492 | HBB | Hemoglobin subunit beta | 10 | 0.00008 | 0.38927 |
| 493 | HEXB | Hexosaminidase Subunit Beta | 10 | 0.00031 | 0.34712 |
| 494 | MYH4 | Myosin-4 | 10 | 0.00065 | 0.37201 |
| 495 | NPHS2 | Podocin | 10 | 0.00143 | 0.37996 |
| 496 | SV2A | Synaptic vesicle glycoprotein 2A | 10 | 0.00248 | 0.37146 |
| 497 | TAZ | Tafazzin | 10 | 0.00172 | 0.33528 |
| 498 | TIMM22 | Mitochondrial import inner membrane translocase subunit Tim22 | 10 | 0.00057 | 0.31242 |
| 499 | ZBTB7B | Zinc finger and BTB domain-containing protein 7B | 10 | 0.00009 | 0.38406 |
| 500 | ALPP | Alkaline Phosphatase, Placental | 9 | 0.00014 | 0.38426 |
| 501 | AZU1 | Azurocidin 1 | 9 | 0.00011 | 0.35948 |
| 502 | CSN2 | Beta-casein | 9 | 0.00000 | 0.39545 |
| 503 | FLNB | Filamin B | 9 | 0.00053 | 0.37900 |
| 504 | FOXE1 | Forkhead Box E1 | 9 | 0.00006 | 0.34078 |
| 505 | FUT1 | Galactoside 2-alpha-L-fucosyltransferase 1 | 9 | 0.00106 | 0.35538 |
| 506 | LAIR1 | Leukocyte-associated immunoglobulin-like receptor 1 | 9 | 0.00006 | 0.34567 |
| 507 | MUC13 | Mucin-13 | 9 | 0.00212 | 0.37054 |
| 508 | ADSSL1 | Adenylosuccinate synthetase isozyme 1 | 8 | 0.00060 | 0.31747 |
| 509 | ANKRD1 | Ankyrin repeat domain-containing protein 1 | 8 | 0.00056 | 0.33755 |
| 510 | AZGP1 | Alpha-2-Glycoprotein 1, Zinc-Binding | 8 | 0.00034 | 0.38015 |
| 511 | CASQ2 | Calsequestrin 2 | 8 | 0.00053 | 0.29737 |
| 512 | CD300LB | CMRF35-like molecule 7 | 8 | 0.00020 | 0.33786 |
| 513 | COMMD5 | COMM domain-containing protein 5 | 8 | 0.00068 | 0.36017 |
| 514 | DCSTAMP | Dendritic cell-specific transmembrane protein | 8 | 0.00081 | 0.36070 |
| 515 | ENTPD8 | Ectonucleoside triphosphate diphosphohydrolase 8 | 8 | 0.00045 | 0.34907 |
| 516 | FCRL3 | Fc Receptor Like 3 | 8 | 0.00003 | 0.34472 |
| 517 | GABPA | GA-binding protein alpha chain | 8 | 0.00055 | 0.36087 |
| 518 | HAS1 | Hyaluronan Synthase 1 | 8 | 0.00012 | 0.37996 |
| 519 | HEXA | Hexosaminidase Subunit Alpha | 8 | 0.00027 | 0.31720 |
| 520 | KCNG3 | Potassium voltage-gated channel subfamily G member 3 | 8 | 0.00077 | 0.28051 |
| 521 | MAPRE3 | Microtubule-associated protein RP/EB family member 3 | 8 | 0.00141 | 0.32922 |
| 522 | MARK3 | MAP/microtubule affinity-regulating kinase 3 | 8 | 0.00001 | 0.37090 |
| 523 | MLYCD | Malonyl-CoA decarboxylase, mitochondrial | 8 | 0.00282 | 0.37313 |
| 524 | PHF6 | PHD finger protein 6 | 8 | 0.00009 | 0.35845 |
| 525 | RANBP2 | E3 SUMO-protein ligase RanBP2 | 8 | 0.00405 | 0.36708 |
| 526 | SERPINA6 | Corticosteroid-binding globulin | 8 | 0.00003 | 0.38327 |
| 527 | SLC7A11 | Solute Carrier Family 7 Member 11 | 8 | 0.00006 | 0.37746 |
| 528 | ST3GAL5 | ST3 Beta-Galactoside Alpha-2,3-Sialyltransferase 5 | 8 | 0.00007 | 0.32677 |
| 529 | ST8SIA1 | ST8 Alpha-N-Acetyl-Neuraminide Alpha-2,8-Sialyltransferase 1 | 8 | 0.00007 | 0.32677 |
| 530 | SV2C | Synaptic vesicle glycoprotein 2C | 8 | 0.00279 | 0.37294 |
| 531 | THBS3 | Thrombospondin-3 | 8 | 0.00010 | 0.33588 |
| 532 | TMEM177 | Transmembrane protein 177 | 8 | 0.00000 | 0.27770 |
| 533 | UGDH | UDP-Glucose 6-Dehydrogenase | 8 | 0.00080 | 0.36333 |
| 534 | WISP2 | WNT1-inducible-signaling pathway protein 2 | 8 | 0.00000 | 0.38886 |
| 535 | ABO | Histo-blood group ABO system transferase | 7 | 0.00035 | 0.34907 |
| 536 | AGXT2 | Alanine--glyoxylate aminotransferase 2, mitochondrial | 7 | 0.00295 | 0.33924 |
| 537 | ANKRD2 | Ankyrin repeat domain-containing protein 2 | 7 | 0.00021 | 0.37164 |
| 538 | ART4 | Ecto-ADP-ribosyltransferase 4 | 7 | 0.00006 | 0.33955 |
| 539 | BPIFA1 | BPI fold-containing family A member 1 | 7 | 0.00001 | 0.37313 |
| 540 | COG8 | Conserved oligomeric Golgi complex subunit 8 | 7 | 0.00161 | 0.36852 |
| 541 | GPR174 | G Protein-Coupled Receptor 174 | 7 | 0.00000 | 0.33832 |
| 542 | HSD17B10 | Hydroxysteroid 17-Beta Dehydrogenase 10 | 7 | 0.00161 | 0.33483 |
| 543 | KCNE3 | Potassium Voltage-Gated Channel Subfamily E Regulatory Subunit 3 | 7 | 0.00044 | 0.28665 |
| 544 | NDUFA4L2 | NADH dehydrogenase [ubiquinone] 1 alpha subcomplex subunit 4-like 2 | 7 | 0.00013 | 0.30341 |
| 545 | PLEKHO1 | Pleckstrin homology domain-containing family O member 1 | 7 | 0.00060 | 0.37880 |
| 546 | PLPP3 | Phospholipid phosphatase 3 | 7 | 0.00035 | 0.35759 |
| 547 | RBMX | RNA-binding motif protein, X chromosome | 7 | 0.00107 | 0.31733 |
| 548 | REG4 | Regenerating islet-derived protein 4 | 7 | 0.00124 | 0.34094 |
| 549 | RGS4 | Regulator of G-protein signaling 4 | 7 | 0.00032 | 0.36798 |
| 550 | SEMA3C | Semaphorin-3C | 7 | 0.00007 | 0.37519 |
| 551 | SF1 | Splicing Factor 1 | 7 | 0.00276 | 0.33694 |
| 552 | SPRN | Shadow of prion protein | 7 | 0.00268 | 0.33082 |
| 745 | STAMBP | STAM-binding protein | 7 | 0.00004 | 0.37594 |
| 553 | ABCB7 | ATP Binding Cassette Subfamily B Member 7 | 6 | 0.00036 | 0.32620 |
| 554 | CASQ1 | Calsequestrin 1 | 6 | 0.00036 | 0.31022 |
| 555 | DMPK | Myotonin-protein kinase | 6 | 0.00269 | 0.36816 |
| 556 | FUT7 | Alpha-(1,3)-fucosyltransferase 7 | 6 | 0.00002 | 0.33725 |
| 557 | GSDME | Gasdermin-E | 6 | 0.00014 | 0.35965 |
| 558 | LONP2 | Lon protease homolog 2, peroxisomal | 6 | 0.00117 | 0.34392 |
| 559 | NR4A3 | Nuclear receptor subfamily 4 group A member 3 | 6 | 0.00000 | 0.37785 |
| 560 | PRELP | Prolargin | 6 | 0.00031 | 0.33679 |
| 561 | PTPN3 | Protein Tyrosine Phosphatase Non-Receptor Type 3 | 6 | 0.00268 | 0.36017 |
| 562 | PYGM | Glycogen phosphorylase, muscle form | 6 | 0.00254 | 0.33878 |
| 563 | RBM25 | RNA Binding Motif Protein 25 | 6 | 0.00314 | 0.36439 |
| 564 | REPS2 | RalBP1-associated Eps domain-containing protein 2 | 6 | 0.00000 | 0.35453 |
| 565 | SCN4A | Sodium Voltage-Gated Channel Alpha Subunit 4 | 6 | 0.00067 | 0.29714 |
| 566 | SHANK2 | SH3 And Multiple Ankyrin Repeat Domains 2 | 6 | 0.00621 | 0.35948 |
| 567 | SMS | Spermine synthase | 6 | 0.00015 | 0.31964 |
| 568 | UBASH3A | Ubiquitin Associated And SH3 Domain Containing A | 6 | 0.00001 | 0.34203 |
| 569 | USH1C | Harmonin | 6 | 0.00290 | 0.29844 |
| 570 | USP43 | Ubiquitin carboxyl-terminal hydrolase 43 | 6 | 0.00011 | 0.31991 |
| 746 | ZFX | Zinc finger X-chromosomal protein | 6 | 0.00014 | 0.35538 |
| 571 | ACP1 | Acid Phosphatase 1 | 5 | 0.00007 | 0.32408 |
| 572 | CALHM6 | Calcium homeostasis modulator protein 6 | 5 | 0.00013 | 0.32980 |
| 573 | CHRNA6 | Neuronal acetylcholine receptor subunit alpha-6 | 5 | 0.00010 | 0.32778 |
| 574 | CLCN7 | H(+)/Cl(-) exchange transporter 7 | 5 | 0.00109 | 0.33244 |
| 575 | CLEC3B | Tetranectin | 5 | 0.00001 | 0.35690 |
| 576 | CRABP1 | Cellular Retinoic Acid Binding Protein 1 | 5 | 0.00269 | 0.32143 |
| 577 | CTRL | Chymotrypsin-like protease CTRL-1 | 5 | 0.00000 | 0.39336 |
| 578 | DBP | D-Box Binding PAR BZIP Transcription Factor | 5 | 0.00010 | 0.36907 |
| 579 | DNAJB7 | DnaJ homolog subfamily B member 7 | 5 | 0.00000 | 0.36708 |
| 580 | EFEMP1 | EGF-containing fibulin-like extracellular matrix protein 1 | 5 | 0.00033 | 0.34125 |
| 581 | FAM167A | Family With Sequence Similarity 167 Member A | 5 | 0.00000 | 0.32763 |
| 582 | FGFR1OP | FGFR1 Oncogene Partner | 5 | 0.00004 | 0.35005 |
| 583 | FTMT | Ferritin, mitochondrial | 5 | 0.00005 | 0.33378 |
| 584 | HSD11B2 | Corticosteroid 11-beta-dehydrogenase isozyme 2 | 5 | 0.00001 | 0.36871 |
| 585 | HSF4 | Heat shock factor protein 4 | 5 | 0.00000 | 0.36636 |
| 586 | KCNE1 | Potassium Voltage-Gated Channel Subfamily E Regulatory Subunit 1 | 5 | 0.00024 | 0.28490 |
| 587 | KCNK3 | Potassium channel subfamily K member 3 | 5 | 0.00067 | 0.30231 |
| 588 | LMOD3 | Leiomodin 3 | 5 | 0.00052 | 0.32115 |
| 589 | MAPK8IP2 | C-Jun-amino-terminal kinase-interacting protein 2 | 5 | 0.00051 | 0.36139 |
| 590 | PHGDH | D-3-phosphoglycerate dehydrogenase | 5 | 0.00085 | 0.35656 |
| 591 | PROZ | Vitamin K-dependent protein Z | 5 | 0.00000 | 0.30048 |
| 592 | RIMS2 | Regulating synaptic membrane exocytosis protein 2 | 5 | 0.00015 | 0.29940 |
| 593 | RPS6KA4 | Ribosomal protein S6 kinase alpha-4 | 5 | 0.00001 | 0.35571 |
| 594 | SLIT2 | Slit Guidance Ligand 2 | 5 | 0.00015 | 0.34047 |
| 595 | SRL | Sarcalumenin | 5 | 0.00013 | 0.28897 |
| 596 | TMEM14C | Transmembrane protein 14C | 5 | 0.00008 | 0.30779 |
| 597 | TMEM86B | Lysoplasmalogenase | 5 | 0.00008 | 0.29761 |
| 747 | TUBB1 | Tubulin beta-1 chain | 5 | 0.00020 | 0.33393 |
| 598 | AFM | Afamin | 4 | 0.00001 | 0.30477 |
| 599 | AGMO | Alkylglycerol monooxygenase | 4 | 0.00017 | 0.32720 |
| 600 | APC2 | Adenomatous polyposis coli protein 2 | 4 | 0.00003 | 0.31112 |
| 601 | ASPDH | Putative L-aspartate dehydrogenase | 4 | 0.00005 | 0.29306 |
| 602 | B3GNT4 | N-acetyllactosaminide beta-1,3-N-acetylglucosaminyltransferase 4 | 4 | 0.00006 | 0.30243 |
| 603 | CARD14 | Caspase recruitment domain-containing protein 14 | 4 | 0.00001 | 0.29892 |
| 604 | CLN3 | CLN3 Lysosomal/Endosomal Transmembrane Protein, Battenin | 4 | 0.00037 | 0.27946 |
| 605 | ELF1 | ETS-related transcription factor Elf-1 | 4 | 0.00001 | 0.33679 |
| 606 | GYG1 | Glycogenin-1 | 4 | 0.00050 | 0.26736 |
| 607 | HLX | H2.0-like homeobox protein | 4 | 0.00000 | 0.34907 |
| 608 | KCNJ18 | Potassium Inwardly Rectifying Channel Subfamily J Member 18 | 4 | 0.00011 | 0.25382 |
| 609 | LACRT | Lacritin | 4 | 0.00001 | 0.34047 |
| 610 | MARCKS | Myristoylated alanine-rich C-kinase substrate | 4 | 0.00000 | 0.38229 |
| 611 | MGAT4C | Alpha-1,3-mannosyl-glycoprotein 4-beta-N-acetylglucosaminyltransferase C | 4 | 0.00047 | 0.36690 |
| 612 | NAPEPLD | N-acyl-phosphatidylethanolamine-hydrolyzing phospholipase D | 4 | 0.00014 | 0.33816 |
| 613 | OMA1 | Metalloendopeptidase OMA1, mitochondrial | 4 | 0.00061 | 0.37127 |
| 614 | PCBD1 | Pterin-4-alpha-carbinolamine dehydratase | 4 | 0.00004 | 0.31532 |
| 615 | PRAM1 | PML-RARA-regulated adapter molecule 1 | 4 | 0.00012 | 0.35236 |
| 616 | REC8 | Meiotic recombination protein REC8 homolog | 4 | 0.00006 | 0.28468 |
| 617 | REEP3 | Receptor expression-enhancing protein 3 | 4 | 0.00049 | 0.31034 |
| 618 | SLC17A8 | Vesicular glutamate transporter 3 | 4 | 0.00008 | 0.31492 |
| 619 | SOHLH2 | Spermatogenesis- and oogenesis-specific basic helix-loop-helix-containing protein 2 | 4 | 0.00058 | 0.34424 |
| 620 | SRGAP2 | SLIT-ROBO Rho GTPase-activating protein 2 | 4 | 0.00269 | 0.31747 |
| 621 | TM6SF2 | Transmembrane 6 superfamily member 2 | 4 | 0.00326 | 0.36618 |
| 622 | TMOD1 | Tropomodulin-1 | 4 | 0.00007 | 0.30791 |
| 623 | TNFAIP2 | Tumor necrosis factor alpha-induced protein 2 | 4 | 0.00000 | 0.34841 |
| 624 | TPP1 | Tripeptidyl Peptidase 1 | 4 | 0.00021 | 0.29514 |
| 625 | XIRP1 | Xin actin-binding repeat-containing protein 1 | 4 | 0.00000 | 0.28242 |
| 626 | ABCF1 | ATP Binding Cassette Subfamily F Member 1 | 3 | 0.00116 | 0.31112 |
| 627 | ACAD10 | Acyl-CoA dehydrogenase family member 10 | 3 | 0.00001 | 0.26900 |
| 628 | ACAD8 | Isobutyryl-CoA dehydrogenase, mitochondrial | 3 | 0.00001 | 0.26900 |
| 629 | ASIC3 | Acid-sensing ion channel 3 | 3 | 0.00000 | 0.33171 |
| 630 | BRSK1 | Serine/threonine-protein kinase BRSK1 | 3 | 0.00023 | 0.30881 |
| 631 | C5AR2 | C5a anaphylatoxin chemotactic receptor 2 | 3 | 0.00000 | 0.35913 |
| 632 | CHRNG | Acetylcholine receptor subunit gamma | 3 | 0.00004 | 0.31034 |
| 633 | CLRN3 | Clarin-3 | 3 | 0.00001 | 0.27575 |
| 634 | DMC1 | Meiotic recombination protein DMC1/LIM15 homolog | 3 | 0.00004 | 0.29549 |
| 635 | ELOVL4 | Elongation of very long chain fatty acids protein 4 | 3 | 0.00003 | 0.29294 |
| 636 | EPN3 | Epsin-3 | 3 | 0.00000 | 0.33215 |
| 637 | GRSF1 | G-rich sequence factor 1 | 3 | 0.00000 | 0.26405 |
| 638 | HAS3 | Hyaluronan Synthase 3 | 3 | 0.00001 | 0.33244 |
| 639 | INPP5F | Phosphatidylinositide phosphatase SAC2 | 3 | 0.00000 | 0.35742 |
| 640 | KIF12 | Kinesin-like protein KIF12 | 3 | 0.00014 | 0.29725 |
| 641 | KIFC2 | Kinesin-like protein KIFC2 | 3 | 0.00011 | 0.28274 |
| 642 | LETMD1 | LETM1 domain-containing protein 1 | 3 | 0.00001 | 0.34956 |
| 643 | MCRIP2 | MAPK regulated corepressor interacting protein 2 | 3 | 0.00001 | 0.26987 |
| 644 | OSTN | Osteocrin | 3 | 0.00000 | 0.36297 |
| 645 | PLA1A | Phospholipase A1 member A | 3 | 0.00000 | 0.34890 |
| 646 | POLR1E | DNA-directed RNA polymerase I subunit RPA49 | 3 | 0.00010 | 0.33200 |
| 647 | RAB19 | Ras-related protein Rab-19 | 3 | 0.00019 | 0.30121 |
| 648 | RBKS | Ribokinase | 3 | 0.00003 | 0.28522 |
| 649 | RCOR2 | REST corepressor 2 | 3 | 0.00001 | 0.36192 |
| 650 | SIRPB1 | Signal-regulatory protein beta-1 isoform 3 | 3 | 0.00009 | 0.30881 |
| 651 | SLC22A10 | Solute carrier family 22 member 10 | 3 | 0.00268 | 0.26851 |
| 652 | SLC24A2 | Sodium/potassium/calcium exchanger 2 | 3 | 0.00156 | 0.30121 |
| 653 | SLC28A1 | Sodium/nucleoside cotransporter 1 | 3 | 0.00040 | 0.30766 |
| 654 | TRIM54 | Tripartite motif-containing protein 54 | 3 | 0.00000 | 0.30133 |
| 655 | TRPM6 | Transient receptor potential cation channel subfamily M member 6 | 3 | 0.00000 | 0.36762 |
| 656 | WDR7 | WD repeat-containing protein 7 | 3 | 0.00015 | 0.28146 |
| 657 | ZC3H11A | Zinc finger CCCH domain-containing protein 11A | 3 | 0.00044 | 0.29283 |
| 748 | ZDHHC15 | Palmitoyltransferase ZDHHC15 | 3 | 0.00116 | 0.30194 |
| 658 | AMIGO2 | Amphoterin-induced protein 2 | 2 | 0.00000 | 0.29168 |
| 659 | ARFGEF2 | Brefeldin A-inhibited guanine nucleotide-exchange protein 2 | 2 | 0.00002 | 0.30133 |
| 660 | CA5A | Carbonic anhydrase 5A, mitochondrial | 2 | 0.00000 | 0.32893 |
| 661 | CATSPERD | Cation channel sperm-associated protein subunit delta | 2 | 0.00000 | 0.27967 |
| 662 | CDC20B | Cell division cycle protein 20 homolog B | 2 | 0.00004 | 0.28285 |
| 663 | CLGN | Calmegin | 2 | 0.00003 | 0.25741 |
| 664 | CYP2A7 | Cytochrome P450 2A7 | 2 | 0.00000 | 0.29572 |
| 665 | DLGAP2 | Disks large-associated protein 2 | 2 | 0.00003 | 0.26574 |
| 666 | DNAJC5B | DnaJ homolog subfamily C member 5B | 2 | 0.00033 | 0.31268 |
| 667 | DNAJC5G | DnaJ homolog subfamily C member 5G | 2 | 0.00004 | 0.31546 |
| 668 | FAM210A | Protein FAM210A | 2 | 0.00000 | 0.30280 |
| 669 | FAR2 | Fatty acyl-CoA reductase 2 | 2 | 0.00000 | 0.29928 |
| 670 | GABRG3 | Gamma-aminobutyric acid receptor subunit gamma-3 | 2 | 0.00013 | 0.28295 |
| 671 | GDE1 | Glycerophosphodiester phosphodiesterase 1 | 2 | 0.00000 | 0.29549 |
| 672 | GLYAT | Glycine N-acyltransferase | 2 | 0.00001 | 0.31073 |
| 673 | GNL1 | G Protein Nucleolar 1 (Putative) | 2 | 0.00005 | 0.25732 |
| 674 | GOT1L1 | Putative aspartate aminotransferase, cytoplasmic 2 | 2 | 0.00000 | 0.32720 |
| 675 | GPM6B | Neuronal membrane glycoprotein M6-b | 2 | 0.00001 | 0.23996 |
| 676 | GPR1 | G-protein coupled receptor 1 | 2 | 0.00008 | 0.31814 |
| 677 | HAPLN2 | Hyaluronan and proteoglycan link protein 2 | 2 | 0.00001 | 0.31164 |
| 678 | IL1RAPL2 | Interleukin 1 Receptor Accessory Protein Like 2 | 2 | 0.00002 | 0.26765 |
| 679 | INHBC | Inhibin beta C chain | 2 | 0.00000 | 0.32033 |
| 680 | KCNMB2 | Calcium-activated potassium channel subunit beta-2 | 2 | 0.00003 | 0.29952 |
| 681 | LRRC8A | Volume-regulated anion channel subunit LRRC8A | 2 | 0.00000 | 0.30391 |
| 682 | MACROD2 | Mono-ADP Ribosylhydrolase 2 | 2 | 0.00000 | 0.31177 |
| 683 | MAN1C1 | Mannosyl-oligosaccharide 1,2-alpha-mannosidase IC | 2 | 0.00001 | 0.27811 |
| 684 | MOGAT3 | 2-acylglycerol O-acyltransferase 3 | 2 | 0.00000 | 0.29868 |
| 685 | MSC | Musculin | 2 | 0.00002 | 0.30304 |
| 686 | MYORG | Myogenesis-regulating glycosidase | 2 | 0.00003 | 0.30932 |
| 687 | NALCN | Sodium leak channel non-selective protein | 2 | 0.00005 | 0.30552 |
| 688 | PBLD | Phenazine biosynthesis-like domain-containing protein | 2 | 0.00000 | 0.29340 |
| 689 | PLCXD1 | PI-PLC X domain-containing protein 1 | 2 | 1.00000 | 1.00000 |
| 690 | PPIL6 | Probable inactive peptidyl-prolyl cis-trans isomerase-like 6 | 2 | 0.00000 | 0.25893 |
| 691 | RAB15 | Ras-related protein Rab-15 | 2 | 0.00000 | 0.27323 |
| 692 | RFXAP | Regulatory factor X-associated protein | 2 | 0.00000 | 0.30317 |
| 693 | SCRG1 | Scrapie-responsive protein 1 | 2 | 0.00000 | 0.31733 |
| 694 | SENP8 | Sentrin-specific protease 8 | 2 | 0.00000 | 0.31164 |
| 695 | SKAP1 | Src kinase-associated phosphoprotein 1 | 2 | 0.00000 | 0.32980 |
| 696 | TBL2 | Transducin beta-like protein 2 | 2 | 0.00012 | 0.26174 |
| 749 | TMEM246 | Transmembrane protein 246 | 2 | 0.00003 | 0.28875 |
| 750 | WDR72 | WD repeat-containing protein 72 | 2 | 0.00000 | 0.28125 |
| 697 | ACOT12 | Acetyl-coenzyme A thioesterase | 1 | 0.00000 | 0.27183 |
| 698 | AJAP1 | Adherens junction-associated protein 1 | 1 | 0.00000 | 0.24867 |
| 699 | ANTXR1 | Anthrax toxin receptor 1 | 1 | 0.00000 | 0.30791 |
| 700 | APOLD1 | Apolipoprotein L Domain Containing 1 | 1 | 0.00000 | 0.32835 |
| 701 | CDR2L | Cerebellar degeneration-related protein 2-like | 1 | 0.00000 | 0.22992 |
| 702 | CLEC14A | C-type lectin domain family 14 member A | 1 | 0.00000 | 0.30791 |
| 703 | CTTNBP2 | Cortactin-binding protein 2 | 1 | 0.00000 | 0.25875 |
| 704 | CYP3A43 | Cytochrome P450 3A43 | 1 | 0.00000 | 0.29100 |
| 705 | DHRSX | Dehydrogenase/reductase SDR family member on chromosome X | 1 | 0.00000 | 0.66667 |
| 706 | DMWD | Dystrophia myotonica WD repeat-containing protein | 1 | 0.00000 | 0.26919 |
| 707 | DNAAF2 | Protein kintoun | 1 | 0.00000 | 0.66667 |
| 708 | EDDM3B | Epididymal secretory protein E3-beta | 1 | 0.00000 | 0.26717 |
| 709 | ENTPD7 | Ectonucleoside triphosphate diphosphohydrolase 7 | 1 | 0.00000 | 1.00000 |
| 710 | IPCEF1 | Interactor protein for cytohesin exchange factors 1 | 1 | 0.00000 | 0.33024 |
| 711 | KRT79 | Keratin, type II cytoskeletal 79 | 1 | 0.00000 | 0.27863 |
| 712 | LMOD1 | Leiomodin 1 | 1 | 0.00000 | 1.00000 |
| 713 | MCUR1 | Mitochondrial calcium uniporter regulator 1 | 1 | 0.00000 | 0.28425 |
| 714 | NEXN | Nexilin F-Actin Binding Protein | 1 | 0.00000 | 0.24967 |
| 715 | OSBPL5 | Oxysterol-binding protein-related protein 5 | 1 | 0.00000 | 0.28842 |
| 716 | RPUSD2 | RNA pseudouridylate synthase domain-containing protein 2 | 1 | 0.00000 | 0.23858 |
| 717 | SHD | SH2 domain-containing adapter protein D | 1 | 0.00000 | 0.26822 |
| 718 | SLAIN1 | SLAIN motif-containing protein 1 | 1 | 0.00000 | 0.29146 |
| 719 | SLC22A18AS | Beckwith-Wiedemann syndrome chromosomal region 1 candidate gene B protein | 1 | 0.00000 | 0.30906 |
| 720 | SLC25A26 | S-adenosylmethionine mitochondrial carrier protein | 1 | 0.00000 | 0.26755 |
| 721 | SLC6A18 | Inactive sodium-dependent neutral amino acid transporter B | 1 | 0.00000 | 0.26861 |
| 722 | SLITRK4 | SLIT and NTRK-like protein 4 | 1 | 0.00000 | 0.26247 |
| 723 | SRGAP2B | SLIT-ROBO Rho GTPase-activating protein 2B | 1 | 0.00000 | 0.27174 |
| 724 | SRGAP2C | SLIT-ROBO Rho GTPase-activating protein 2C | 1 | 0.00000 | 0.24105 |
| 725 | SSMEM1 | Serine-rich single-pass membrane protein 1 | 1 | 0.00000 | 0.27453 |
| 726 | TMC4 | Transmembrane channel-like protein 4 | 1 | 0.00000 | 0.26813 |
| 727 | UACA | Uveal Autoantigen With Coiled-Coil Domains And Ankyrin Repeats | 1 | 0.00000 | 0.34988 |
| 728 | WWC2 | Protein WWC2 | 1 | 0.00000 | 0.26489 |
| 729 | ZNF117 | Zinc finger protein 117 | 1 | 0.00000 | 0.21173 |
| 751 | ACSM2A | Acyl-coenzyme A synthetase ACSM2A, mitochondrial | 0 | 0.00000 | 0.00000 |
| 752 | ACSM5 | Acyl-coenzyme A synthetase ACSM5, mitochondrial | 0 | 0.00000 | 0.00000 |
| 753 | ANXA10 | Annexin A10 | 0 | 0.00000 | 0.00000 |
| 754 | BTBD16 | BTB/POZ domain-containing protein 16 | 0 | 0.00000 | 0.00000 |
| 755 | C11ORF24 | Uncharacterized protein C11orf24 | 0 | 0.00000 | 0.00000 |
| 756 | C16ORF46 | Uncharacterized protein C16orf46 | 0 | 0.00000 | 0.00000 |
| 757 | C1QTNF5 | Complement C1q tumor necrosis factor-related protein 5 | 0 | 0.00000 | 0.00000 |
| 758 | C4ORF3 | Uncharacterized protein C4orf3 | 0 | 0.00000 | 0.00000 |
| 759 | CCDC153 | Coiled-coil domain-containing protein 153 | 0 | 0.00000 | 0.00000 |
| 760 | CDHR1 | Cadherin-related family member 1 | 0 | 0.00000 | 0.00000 |
| 761 | CLDN23 | Claudin-23 | 0 | 0.00000 | 0.00000 |
| 762 | DDTL | D-dopachrome decarboxylase-like protein | 0 | 0.00000 | 0.00000 |
| 763 | EFCAB1 | EF-hand calcium-binding domain-containing protein 1 | 0 | 0.00000 | 0.00000 |
| 764 | ELMOD3 | ELMO domain-containing protein 3 | 0 | 0.00000 | 0.00000 |
| 765 | ERICH6 | Glutamate-rich protein 6 | 0 | 0.00000 | 0.00000 |
| 766 | ETNK2 | Ethanolamine kinase 2 | 0 | 0.00000 | 0.00000 |
| 767 | FAM166A | Protein FAM166A | 0 | 0.00000 | 0.00000 |
| 768 | FAM241A | Uncharacterized protein FAM241A | 0 | 0.00000 | 0.00000 |
| 769 | FUOM | Fucose mutarotase | 0 | 0.00000 | 0.00000 |
| 770 | GBA3 | Cytosolic beta-glucosidase | 0 | 0.00000 | 0.00000 |
| 771 | GCM1 | Chorion-specific transcription factor GCMa | 0 | 0.00000 | 0.00000 |
| 772 | GGT3P | Gamma-Glutamyltransferase 3 Pseudogene | 0 | 0.00000 | 0.00000 |
| 773 | GGTA1P | Inactive N-acetyllactosaminide alpha-1,3-galactosyltransferase | 0 | 0.00000 | 0.00000 |
| 774 | GOLGA8A | Golgin subfamily A member 8A | 0 | 0.00000 | 0.00000 |
| 775 | GSTT1 | Glutathione S-Transferase Theta 1 | 0 | 0.00000 | 0.00000 |
| 776 | HILPDA | Hypoxia-inducible lipid droplet-associated protein | 0 | 0.00000 | 0.00000 |
| 777 | HLA-DRB3 | Major Histocompatibility Complex, Class II, DR Beta 3 | 0 | 0.00000 | 0.00000 |
| 778 | IGHE | Immunoglobulin Heavy Constant Epsilon | 0 | 0.00000 | 0.00000 |
| 779 | IGKC | Immunoglobulin kappa constant | 0 | 0.00000 | 0.00000 |
| 780 | KIAA0040 | Uncharacterized protein KIAA0040 | 0 | 0.00000 | 0.00000 |
| 781 | LELP1 | Late cornified envelope-like proline-rich protein 1 | 0 | 0.00000 | 0.00000 |
| 782 | LINGO4 | Leucine-rich repeat and immunoglobulin-like domain-containing nogo receptor-interacting protein 4 | 0 | 0.00000 | 0.00000 |
| 783 | LYRM1 | LYR motif-containing protein 1 | 0 | 0.00000 | 0.00000 |
| 784 | LYZL1 | Lysozyme-like protein 1 | 0 | 0.00000 | 0.00000 |
| 785 | MB21D2 | Protein MB21D2 | 0 | 0.00000 | 0.00000 |
| 786 | MS4A10 | Membrane-spanning 4-domains subfamily A member 10 | 0 | 0.00000 | 0.00000 |
| 787 | OIT3 | Oncoprotein-induced transcript 3 protein | 0 | 0.00000 | 0.00000 |
| 788 | ORAI3 | Protein orai-3 | 0 | 0.00000 | 0.00000 |
| 789 | PACC1 | Proton-activated chloride channel | 0 | 0.00000 | 0.00000 |
| 790 | PALD1 | Paladin | 0 | 0.00000 | 0.00000 |
| 791 | PHETA1 | Sesquipedalian-1 | 0 | 0.00000 | 0.00000 |
| 792 | PLSCR2 | Phospholipid scramblase 2 | 0 | 0.00000 | 0.00000 |
| 793 | PRM2 | Protamine-2 | 0 | 0.00000 | 0.00000 |
| 794 | RBMY1A1 | RNA-binding motif protein, Y chromosome, family 1 member A1 | 0 | 0.00000 | 0.00000 |
| 795 | RINL | Ras and Rab interactor-like protein | 0 | 0.00000 | 0.00000 |
| 796 | RIOX1 | Ribosomal oxygenase 1 | 0 | 0.00000 | 0.00000 |
| 797 | SH3TC1 | SH3 domain and tetratricopeptide repeat-containing protein 1 | 0 | 0.00000 | 0.00000 |
| 798 | SLC25A24 | Calcium-binding mitochondrial carrier protein SCaMC-1 | 0 | 0.00000 | 0.00000 |
| 799 | SLC25A34 | Solute carrier family 25 member 34 | 0 | 0.00000 | 0.00000 |
| 800 | SLC25A42 | Mitochondrial coenzyme A transporter SLC25A42 | 0 | 0.00000 | 0.00000 |
| 801 | SLC26A11 | Sodium-independent sulfate anion transporter | 0 | 0.00000 | 0.00000 |
| 802 | SLC43A3 | Solute carrier family 43 member 3 | 0 | 0.00000 | 0.00000 |
| 803 | SLC7A10 | Asc-type amino acid transporter 1 | 0 | 0.00000 | 0.00000 |
| 804 | SNAI3 | Zinc finger protein SNAI3 | 0 | 0.00000 | 0.00000 |
| 805 | SRY | Sex-determining region Y protein | 0 | 0.00000 | 0.00000 |
| 806 | TBC1D21 | TBC1 domain family member 21 | 0 | 0.00000 | 0.00000 |
| 807 | TMEM41A | Transmembrane protein 41A | 0 | 0.00000 | 0.00000 |
| 808 | VPS37D | Vacuolar protein sorting-associated protein 37D | 0 | 0.00000 | 0.00000 |
| 809 | YPEL4 | Protein yippee-like 4 | 0 | 0.00000 | 0.00000 |
| 810 | ZCWPW2 | Zinc finger CW-type PWWP domain protein 2 | 0 | 0.00000 | 0.00000 |
| 811 | ZFY | Zinc finger Y-chromosomal protein | 0 | 0.00000 | 0.00000 |
| 812 | ZNF681 | Zinc finger protein 681 | 0 | 0.00000 | 0.00000 |
| 813 | ZSWIM2 | E3 ubiquitin-protein ligase ZSWIM2 | 0 | 0.00000 | 0.00000 |
