# Supplementary material for: Mechanisms of Spica Prunellae against thyroid-associated Ophthalmopathy based on network pharmacology and molecular docking
Source: BMC Complement Med Ther. 2020 Jul 20;20:229. doi: 10.1186/s12906-020-03022-2 (PMC7372882; doi:10.1186/s12906-020-03022-2)
Supplement: Supplementary file 4 — Additional file 4: Table S4 Information for overlapped targets after PPI analysis. [file 12906_2020_3022_MOESM4_ESM.docx]

**Table S4** Information for overlapped targets after PPI analysis.

| **Number** | **Name** | **Protein name** | **Degree** | **Betweenness Centrality** | **Closeness Centrality** |
| --- | --- | --- | --- | --- | --- |
| 1 | IL6 | Interleukin-6 | 97 | 0.04494038 | 0.80769231 |
| 2 | ALB | Serum albumin | 95 | 0.05933185 | 0.79746835 |
| 3 | AKT1 | RAC-alpha serine/threonine-protein kinase | 93 | 0.04999615 | 0.7875 |
| 4 | INS | Insulin | 92 | 0.06290674 | 0.7875 |
| 5 | TNF | Tumor Necrosis Factor | 91 | 0.03182703 | 0.77777778 |
| 6 | VEGFA | Vascular Endothelial Growth Factor A | 90 | 0.03149661 | 0.76829268 |
| 7 | MAPK3 | Mitogen-activated protein kinase 3 | 88 | 0.0350344 | 0.76363636 |
| 8 | TP53 | Tumor Protein P53 | 82 | 0.0327392 | 0.73684211 |
| 9 | PTGS2 | Prostaglandin-Endoperoxide Synthase 2 | 79 | 0.03139558 | 0.72413793 |
| 10 | STAT3 | Signal Transducer And Activator Of Transcription 3 | 79 | 0.01703349 | 0.72 |
| 11 | CXCL8 | Interleukin-8 | 76 | 0.01166717 | 0.70786517 |
| 12 | IL1B | Interleukin-1 beta | 76 | 0.03492974 | 0.71186441 |
| 13 | JUN | Transcription factor AP-1 | 75 | 0.01155608 | 0.70786517 |
| 14 | MAPK1 | Mitogen-activated protein kinase 1 | 75 | 0.01869412 | 0.70786517 |
| 15 | CASP3 | Caspase-3 | 74 | 0.01370943 | 0.70391061 |
| 16 | EGF | Epidermal Growth Factor | 74 | 0.01575339 | 0.7 |
| 17 | IL10 | Interleukin-10 | 73 | 0.01187296 | 0.6961326 |
| 18 | MMP9 | Matrix Metallopeptidase 9 | 72 | 0.00938383 | 0.69230769 |
| 19 | CCL2 | C-C Motif Chemokine Ligand 2 | 70 | 0.00758357 | 0.68478261 |
| 20 | ICAM1 | Intercellular Adhesion Molecule 1 | 64 | 0.00816083 | 0.66315789 |
| 21 | IL4 | Interleukin-4 | 63 | 0.00565527 | 0.65968586 |
| 22 | TLR4 | Toll Like Receptor 4 | 63 | 0.00564658 | 0.65968586 |
| 23 | CCND1 | CCN family member 2 | 62 | 0.01663216 | 0.65625 |
| 24 | IL2 | Interleukin 2 | 60 | 0.00467063 | 0.64948454 |
| 25 | STAT1 | Signal transducer and activator of transcription 1-alpha/beta | 58 | 0.00950399 | 0.64285714 |
| 26 | IFNG | Interferon gamma | 57 | 0.00344925 | 0.63959391 |
| 27 | PPARG | Peroxisome Proliferator Activated Receptor Gamma | 57 | 0.00754576 | 0.63959391 |
| 28 | VCAM1 | Vascular Cell Adhesion Molecule 1 | 57 | 0.00364268 | 0.63959391 |
| 29 | PTEN | Phosphatase And Tensin Homolog | 54 | 0.00517307 | 0.62686567 |
| 30 | BCL2L1 | BCL2 Like 1 | 53 | 0.00443311 | 0.62686567 |
| 31 | CAT | Catalase | 53 | 0.00707408 | 0.62686567 |
| 32 | MPO | Myeloperoxidase | 53 | 0.0085159 | 0.62686567 |
| 33 | CXCL10 | C-X-C Motif Chemokine Ligand 10 | 52 | 0.0056741 | 0.62376238 |
| 34 | RELA | Transcription factor p65 | 52 | 0.01765877 | 0.62376238 |
| 35 | ERBB2 | Erb-B2 Receptor Tyrosine Kinase 2 | 50 | 0.00906854 | 0.61463415 |
| 36 | HIF1A | Hypoxia-inducible factor 1-alpha | 50 | 0.0032205 | 0.61764706 |
| 37 | HMOX1 | Heme oxygenase 1 | 50 | 0.00449965 | 0.61764706 |
| 38 | SIRT1 | NAD-dependent protein deacetylase sirtuin-1 | 50 | 0.00442793 | 0.61764706 |
| 39 | TGFB1 | Transforming Growth Factor Beta 1 | 50 | 0.00238769 | 0.61764706 |
| 40 | SERPINE1 | Serpin Family E Member 1 | 49 | 0.00252269 | 0.61165049 |
| 41 | CASP8 | Caspase 8 | 48 | 0.00358533 | 0.61165049 |
| 42 | CD40LG | C-C Motif Chemokine Receptor 1 | 47 | 0.00167856 | 0.60869565 |
| 43 | TLR9 | Toll Like Receptor 9 | 47 | 0.00092744 | 0.60869565 |
| 44 | NOS2 | Nitric oxide synthase, inducible | 46 | 0.00225057 | 0.60869565 |
| 45 | FASLG | Fas Ligand | 45 | 0.0011786 | 0.60287081 |
| 46 | CD86 | CD80 Molecule | 44 | 0.00315148 | 0.5971564 |
| 47 | IL5 | Interleukin-5 | 43 | 0.00111772 | 0.59433962 |
| 48 | NFKB1 | Nuclear Factor Kappa B Subunit 1 | 43 | 0.0018329 | 0.59433962 |
| 49 | NR3C1 | Glucocorticoid receptor | 43 | 0.00990803 | 0.5971564 |
| 50 | SELE | Selectin E | 43 | 0.00137971 | 0.5971564 |
| 51 | MMP3 | Matrix Metallopeptidase 3 | 42 | 0.00269206 | 0.5915493 |
| 52 | AR | Androgen receptor | 40 | 0.00207064 | 0.58604651 |
| 53 | CXCL2 | C-X-C motif chemokine 2 | 40 | 0.00089146 | 0.57534247 |
| 54 | CD80 | CD40 Ligand | 39 | 0.00103016 | 0.58333333 |
| 55 | CDKN1A | CD86 Molecule | 39 | 0.00107364 | 0.58333333 |
| 56 | CTGF | Collagen alpha-1 | 39 | 0.00128098 | 0.58333333 |
| 57 | IL1A | Interleukin 1 Alpha | 38 | 0.00062592 | 0.57798165 |
| 58 | SELL | Selectin L | 37 | 0.00136291 | 0.57534247 |
| 59 | SOD1 | Superoxide Dismutase 1 | 37 | 0.0032768 | 0.58064516 |
| 60 | CCL11 | Eotaxin | 35 | 0.00083227 | 0.5625 |
| 61 | NFE2L2 | Nuclear factor erythroid 2-related factor 2 | 35 | 0.0018656 | 0.57534247 |
| 62 | IGF1R | Insulin-like growth factor 1 receptor | 33 | 0.00081199 | 0.56756757 |
| 63 | IRF1 | Interferon regulatory factor 1 | 33 | 0.00033894 | 0.56756757 |
| 64 | PARP1 | Poly(ADP-Ribose) Polymerase 1 | 31 | 0.00082256 | 0.5625 |
| 65 | ABCB1 | ATP-dependent translocase ABCB1 | 30 | 0.00473294 | 0.55752212 |
| 66 | ABCG2 | ATP-binding cassette sub-family G member 2 | 30 | 0.00471777 | 0.55263158 |
| 67 | CCR1 | Cyclin D1 | 30 | 0.00027452 | 0.54077253 |
| 68 | VDR | Vitamin D Receptor | 30 | 0.00098361 | 0.56 |
| 69 | CXCL11 | C-X-C Motif Chemokine Ligand 11 | 29 | 0.00020759 | 0.53164557 |
| 70 | CYP19A1 | Aromatase | 29 | 0.00382248 | 0.55506608 |
| 71 | IGF2 | Insulin Like Growth Factor 2 | 29 | 0.0004945 | 0.55506608 |
| 72 | AGTR1 | Type-1 angiotensin II receptor | 28 | 0.00134538 | 0.55506608 |
| 73 | GSTP1 | Glutathione S-Transferase Pi 1 | 27 | 0.00368545 | 0.55021834 |
| 74 | IGFBP3 | Insulin Like Growth Factor Binding Protein 3 | 27 | 0.00028281 | 0.55021834 |
| 75 | COL1A1 | Cyclin-dependent kinase inhibitor 1 | 26 | 0.00223393 | 0.55021834 |
| 76 | GFAP | Glial fibrillary acidic protein | 26 | 0.00086444 | 0.55021834 |
| 77 | ITGB2 | Integrin Subunit Beta 2 | 25 | 0.00093086 | 0.51851852 |
| 78 | CYP1A1 | Cytochrome P450 Family 1 Subfamily A Member 1 | 24 | 0.00275754 | 0.53164557 |
| 79 | EZH2 | Histone-lysine N-methyltransferase EZH2 | 24 | 0.00072452 | 0.54310345 |
| 80 | CYP3A4 | Cytochrome P450 3A4 | 23 | 0.00322858 | 0.53389831 |
| 81 | ESR2 | Estrogen Receptor 2 | 23 | 0.00066865 | 0.54077253 |
| 82 | PPARA | Peroxisome Proliferator Activated Receptor Alpha | 23 | 0.00127855 | 0.54545455 |
| 83 | RB1 | Retinoblastoma-associated protein | 23 | 0.00033099 | 0.53164557 |
| 84 | GSR | Glutathione-Disulfide Reductase | 22 | 0.01653711 | 0.54310345 |
| 85 | BAX | Apoptosis regulator BAX | 20 | 0.00057345 | 0.52719665 |
| 86 | MMP10 | Stromelysin-2 | 20 | 0 | 0.53389831 |
| 87 | MMP12 | Macrophage metalloelastase | 19 | 0.00009268 | 0.52719665 |
| 88 | PLAT | Tissue-type plasminogen activator | 19 | 0.0001116 | 0.53164557 |
| 89 | PTGER4 | Prostaglandin E2 receptor EP4 subtype | 18 | 0.0013784 | 0.51851852 |
| 90 | S1PR1 | Sphingosine-1-Phosphate Receptor 1 | 18 | 0.00011516 | 0.51428571 |
| 91 | ADRB2 | Adrenoceptor Beta 2 | 17 | 0.00101965 | 0.52719665 |
| 92 | GSTM1 | Glutathione S-transferase Mu 1 | 17 | 0.00154963 | 0.50199203 |
| 93 | ITGA4 | Integrin Subunit Alpha 4 | 17 | 0.00033477 | 0.4921875 |
| 94 | NAMPT | Nicotinamide Phosphoribosyltransferase | 17 | 0.00014557 | 0.525 |
| 95 | RARA | Retinoic Acid Receptor Alpha | 17 | 0.00211733 | 0.51851852 |
| 96 | FABP4 | Fatty acid-binding protein, adipocyte | 15 | 0.00023574 | 0.52066116 |
| 97 | MAPT | Microtubule-associated protein tau | 15 | 0.00021244 | 0.51851852 |
| 98 | ALPL | Alkaline phosphatase, tissue-nonspecific isozyme | 14 | 0.00027081 | 0.51639344 |
| 99 | BCL2 | Apoptosis regulator Bcl-2 | 14 | 0.00021823 | 0.50199203 |
| 100 | MME | Membrane Metalloendopeptidase | 14 | 0.00074736 | 0.51219512 |
| 101 | SNCA | Alpha-synuclein | 14 | 0.00009749 | 0.50806452 |
| 102 | CYP1B1 | Cytochrome P450 1B1 | 13 | 0.00034288 | 0.504 |
| 103 | CYP2C9 | Cytochrome P450 2C9 | 13 | 0.00047711 | 0.5 |
| 104 | ADORA2A | Adenosine receptor A2a | 12 | 0.00001209 | 0.51639344 |
| 105 | EDNRB | Endothelin receptor type B | 12 | 0.00023565 | 0.51219512 |
| 106 | CYP2C19 | Cytochrome P450 2C19 | 10 | 0.00015099 | 0.47908745 |
| 107 | CYP2C8 | Cytochrome P450 2C8 | 10 | 0.00012 | 0.47191011 |
| 108 | CYP2D6 | Cytochrome P450 Family 2 Subfamily D Member 6 | 10 | 0.00026857 | 0.46666667 |
| 109 | LYZ | Lysozyme | 10 | 0.00011025 | 0.49606299 |
| 110 | SCD | Stearoyl-CoA Desaturase | 10 | 0.00024439 | 0.50199203 |
| 111 | HSD11B1 | Corticosteroid 11-beta-dehydrogenase isozyme 1 | 9 | 0.00061823 | 0.48837209 |
| 112 | BAK1 | BCL2 Antagonist/Killer 1 | 8 | 0.00002173 | 0.47908745 |
| 113 | EDNRA | Endothelin Receptor Type A | 8 | 0.00013447 | 0.48091603 |
| 114 | XDH | Xanthine Dehydrogenase | 8 | 0.00001337 | 0.48648649 |
| 115 | FCER2 | Low affinity immunoglobulin epsilon Fc receptor | 7 | 0.00001058 | 0.46494465 |
| 116 | HAS2 | Hyaluronan Synthase 2 | 6 | 0.00004329 | 0.47191011 |
| 117 | THRA | Thyroid Hormone Receptor Alpha | 6 | 0.0159753 | 0.46323529 |
| 118 | THRB | Thyroid Hormone Receptor Beta | 6 | 0.00029637 | 0.47014925 |
| 119 | PTGER2 | Prostaglandin E Receptor 2 | 5 | 0.00001051 | 0.46840149 |
| 120 | HERC5 | E3 ISG15--protein ligase HERC5 | 4 | 0 | 0.47014925 |
| 121 | HSD11B2 | Corticosteroid 11-beta-dehydrogenase isozyme 2 | 3 | 0 | 0.45323741 |
| 122 | SERPINA6 | Corticosteroid-binding globulin | 3 | 0 | 0.46323529 |
| 123 | ACP1 | Acid Phosphatase 1 | 1 | 0 | 0.35294118 |
| 124 | CA5A | Carbonic anhydrase 5A, mitochondrial | 1 | 0 | 0.31738035 |
| 125 | MAPK8IP2 | C-Jun-amino-terminal kinase-interacting protein 2 | 1 | 0 | 0.41721854 |
| 126 | PYGM | Glycogen phosphorylase, muscle form | 1 | 0 | 0.3853211 |
| 127 | RGS4 | Regulator of G-protein signaling 4 | 1 | 0 | 0.44210526 |
